# Disperse-reactive dyes for sustainable waterless textile dyeing: current status, challenges, and future perspectives

**DOI:** 10.1039/d6ra07833h

**Published:** 2026-10-07

**Authors:** Mousa Sadeghi-Kiakhani, Mohammad-Mahdi Norouzi, Elaheh Hashemi, Ali R. Tehrani-Bagha

**Affiliations:** a Department of Organic Colorants, Institute for Color Science and Technology Tehran Iran sadeghi-mo@icrc.ac.ir +98 21 22947537 +98 21 22944184; b School of Chemistry, College of Science, University of Tehran Tehran Iran mm.norouzi@ut.ac.ir; c Department of Chemistry, Faculty of Sciences, Shahid Rajaee Teacher Training University Tehran Iran e.hashemi@sru.ac.ir; d Department of Bioproducts and Biosystems, Aalto University Espoo Finland

## Abstract

Disperse-Reactive (DR) dyes represent a promising class of hybrid colorants that combine the hydrophobic characteristics of disperse dyes with reactive functional groups, offering a potential solution for sustainable dyeing of polyester/cotton blends. This review critically evaluates the reported experimental validation and theoretical potential of DR dyes across five waterless or low-water coloration platforms: supercritical carbon dioxide (SC-CO_2_), inkjet digital printing, solvent-assisted systems, reverse micelle methods, and ionic liquid/deep eutectic solvent (ILs/DES) systems. While SC-CO_2_ dyeing has been the most extensively investigated platform, with over twenty published studies demonstrating the application of various DR dyes on cotton, polyester, silk, wool, nylon, and blended fabrics, other platforms remain largely unexplored. Solvent-assisted dyeing using decamethylcyclopentasiloxane (D5) has shown preliminary promise in a single study, whereas inkjet digital printing and reverse micelle methods have only one and zero experimental reports, respectively. Notably, no experimental study has yet employed DR dyes in ILs/DES media, and their application in this context remains purely conceptual. Our comparative analysis indicates that Inkjet digital printing is mature for standard inks, but its application with DR dyes is at an early stage and the maturity of DR formulations is not established. SC-CO_2_ holds long-term potential, but its superiority is not established. This review identifies critical research gaps, particularly the absence of experimental studies in IL/DES and reverse micelle systems and limited validation in inkjet-related systems, and outlines a roadmap for future molecular engineering of DR dyes including optimized reactive groups, enhanced solubility in non-aqueous media, and improved fixation efficiency to bridge the gap between conceptual promise and practical industrial implementation. Collectively, these findings position DR dyes as an emerging and promising, yet experimentally under-validated, enabling chemistry for next-generation sustainable textile coloration.

## Abstract 2

Disperse-reactive dyes combine hydrophobic chromophores with reactive groups. This review assesses their synthesis, evidence for waterless textile coloration, and barriers to industrial application.

## Graphical abstract

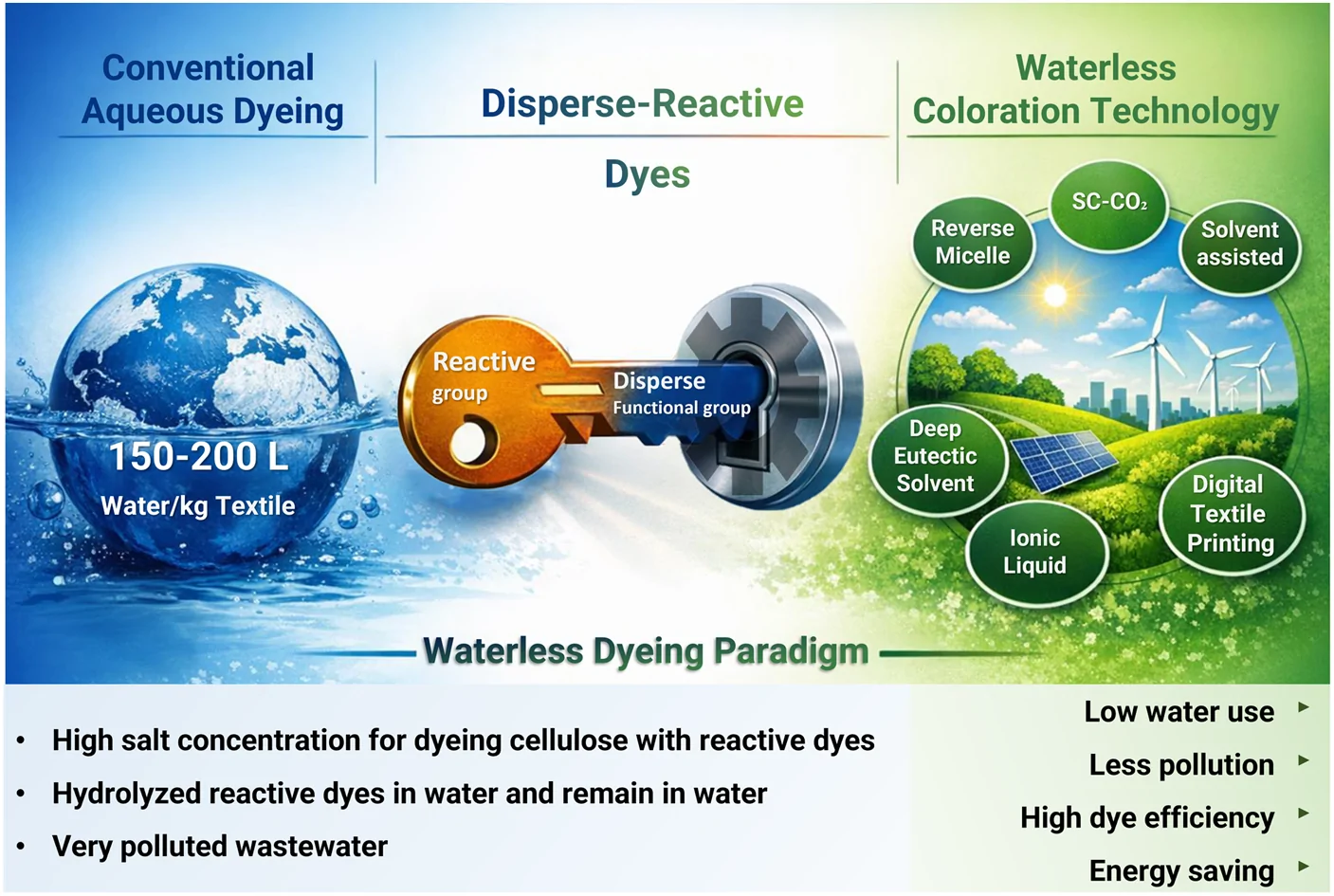

## 1. Introduction

The textile industry is among the most resource-intensive manufacturing sectors, consuming approximately 79–93 billion m^3^ of freshwater annually and generating substantial volumes of dye-contaminated wastewater.^1,2^ An estimated 20% of applied dyes may enter wastewater streams, with 40 000–50 000 tons of dyes released into aquatic environments each year.^3–5^ This intensive resource consumption and wastewater generation make conventional textile coloration a major challenge for sustainable manufacturing.^6^ As illustrated in Fig. 1, conventional dyeing operations are characterized by excessive water usage, substantial wastewater production, and a considerable ecological footprint.

**Fig. 1:**
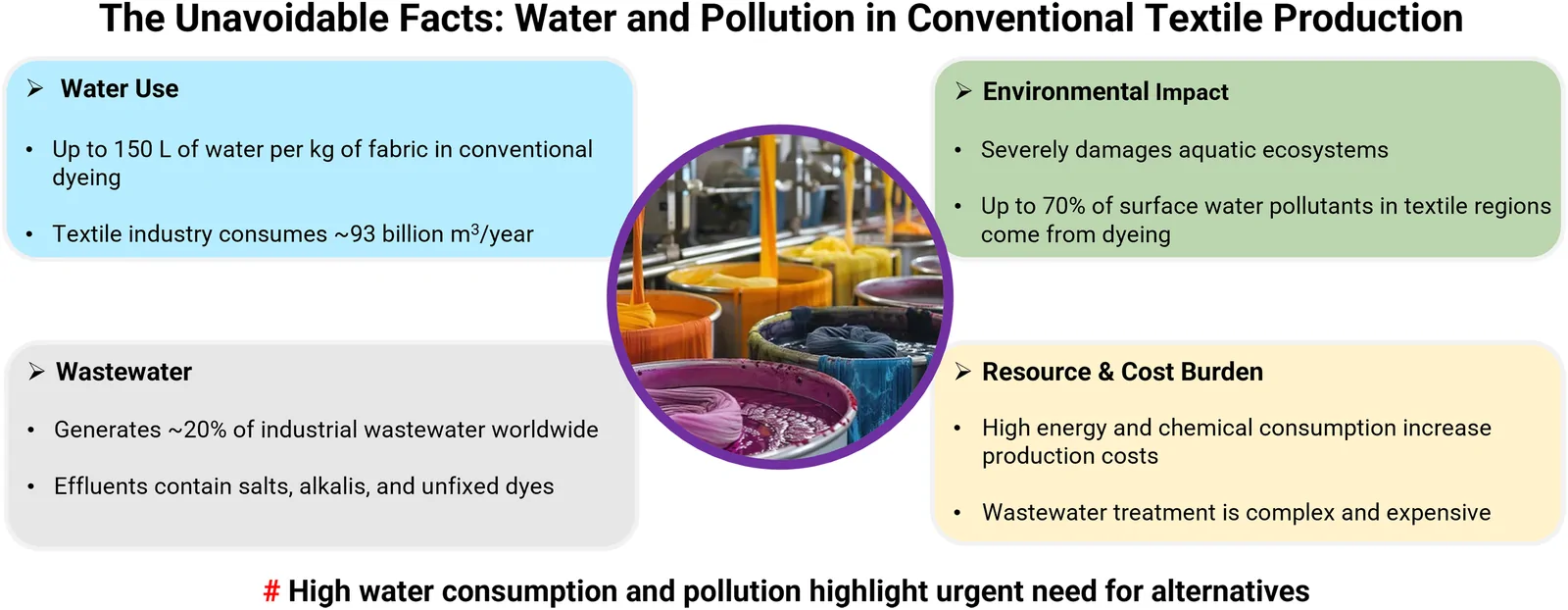
Environmental burden of conventional textile coloration: a synthesized overview presenting key quantitative indicators.

Recent market and production data indicate that global fiber output reached a historic high of 132 million metric tons (Mt) in 2024, continuing a decade-long upward trajectory. Synthetic fibers dominate the current landscape, with polyester alone accounting for roughly 60% of worldwide fiber production, solidifying its status as the most prevalent textile material. Cotton remains the leading natural fiber, contributing approximately 20% to global output, whereas man-made cellulosic fibers (MMCFs), including viscose, lyocell, and modal, represent around 6% of total production. Collectively, polyester and cellulosic fibers (cotton plus MMCFs) constitute nearly 85% of the global textile market, highlighting their overwhelming dominance in manufacturing and downstream processing.

Due to its hydrophobic and highly crystalline architecture, polyester is conventionally dyed with disperse dyes under high-temperature aqueous conditions (typically 120–130 °C) to facilitate polymer penetration. Cotton and other cellulosic materials, in contrast, are primarily dyed with reactive dyes that form covalent bonds with cellulose hydroxyl groups in alkaline aqueous baths, frequently requiring substantial electrolyte additions (commonly NaCl and Na_2_SO_4_) to drive dye exhaustion. Polyester/cotton (PET/CO) blends are among the most extensively used textile substrates worldwide; therefore, they often require sequential or two-bath dyeing sequences, which escalate water consumption, energy input, chemical usage, and processing duration.^7^ Owing to the fundamentally disparate chemical affinities of synthetic *versus* natural fibers, conventional coloration relies on an two-stage approach: disperse dyes for polyester and reactive dyes for cotton.^8^ As schematically depicted in Fig. 2 (path A), this methodology leads to excessive water demand, elevated energy requirements, intensive chemical utilization, and the generation of complex wastewater streams laden with salts, alkalis, and unfixed colorants.^9^ These factors not only raise production costs but also impose severe burdens on wastewater treatment infrastructure, thereby limiting the sustainability and scalability of blended-fabric dyeing.^10^

**Fig. 2:**
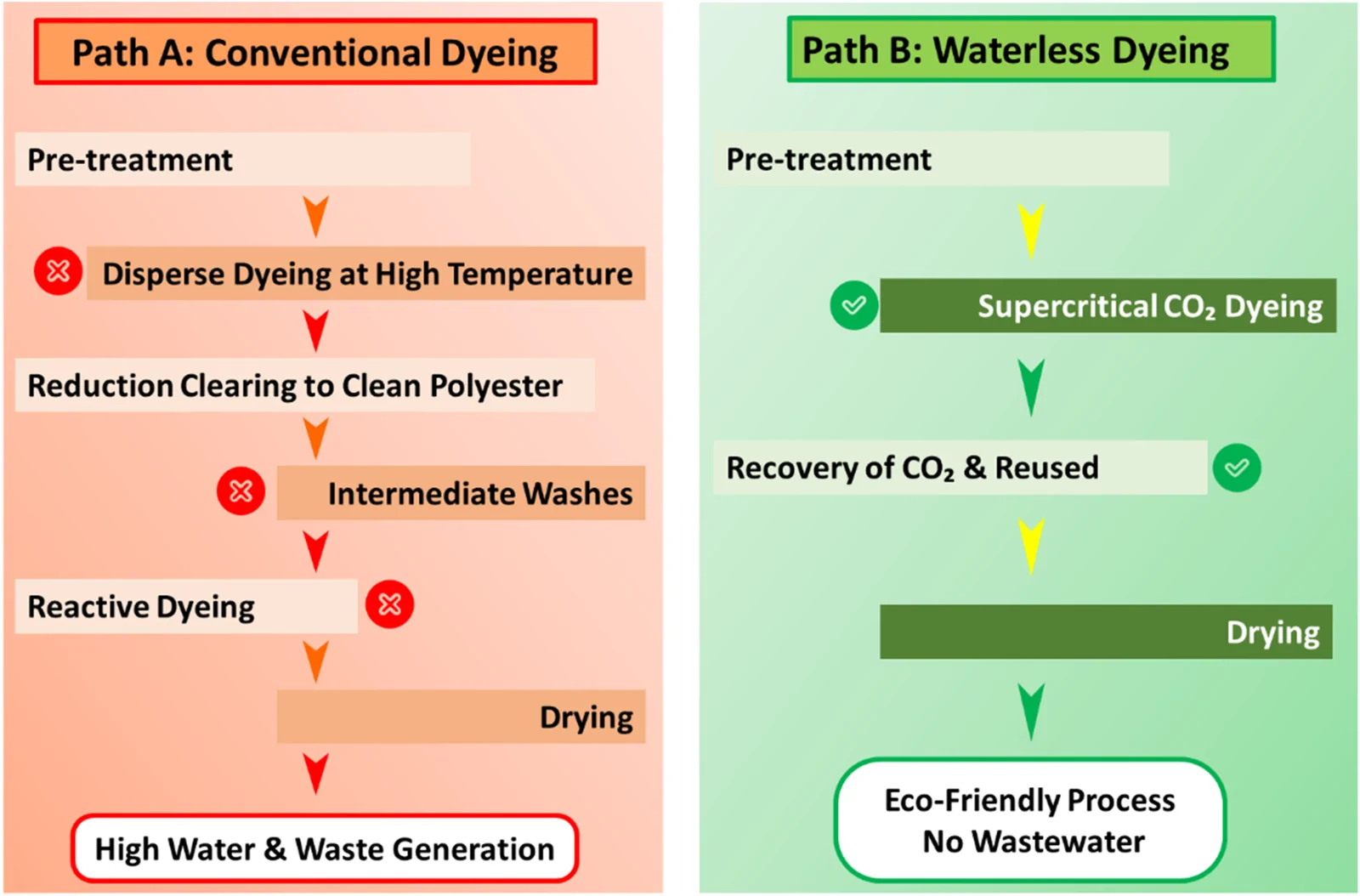
Schematic workflow: conventional dyeing (path A) *vs.* innovative waterless dyeing using SC-CO_2_ dyeing (path B) for polyester/cotton blends.

Mounting pressure from stricter environmental regulations, intensifying global water scarcity, and rising consumer preference for sustainable products is compelling the textile industry to fundamentally reconsider conventional wet-processing paradigms.^11^ In response, waterless and low-water coloration technologies, including supercritical carbon dioxide (SC-CO_2_) dyeing, inkjet digital printing, ionic liquid/deep eutectic solvent (IL/DES)-assisted dyeing, reverse micelle systems, and solvent-assisted dyeing, have emerged as promising alternatives capable of drastically reducing water abstraction and effluent discharge.^12^ Nevertheless, the industrial uptake of these technologies has been severely constrained by a critical materials bottleneck: many conventional water-soluble dye formulations are poorly compatible with non-aqueous media.^13^ Without the development of compatible colorants, the full environmental and economic potential of waterless dyeing technologies cannot be realized.^14^

DR dyes offer a strategically important resolution to this limitation. These hybrid chromophores are molecularly engineered to combine the hydrophobic segments characteristic of disperse dyes with the covalently reactive functional groups analogous to those found in reactive dyes.^15^ This dual architecture enables simultaneous interaction with both polyester and cotton fibers, thereby allowing effective single-step coloration of blended fabrics.^16^

Supercritical carbon dioxide (SC-CO_2_) dyeing exemplifies a waterless technology in which DR dyes could play a transformative role. In its supercritical state, CO_2_ behaves as a non-polar, hydrophobic medium, rendering it highly suitable for dyeing hydrophobic fibers such as polyester using conventional disperse dyes. However, this environment is inherently incompatible with the conventional dyeing of cellulosic fibers, which typically requires water-soluble reactive dyes and aqueous alkaline conditions to promote fiber swelling and covalent fixation. Consequently, conventional reactive dyes display poor solubility, limited diffusion, and inefficient fixation within SC-CO_2_ systems.

To achieve effective single-step coloration of polyester/cotton blends in SC-CO_2_, dyes must possess both adequate affinity for the hydrophobic supercritical medium and the ability to chemically react with cellulose. DR dyes that are designed with an appropriate hydrophobic–reactive balance and controlled solubility in supercritical or solvent-based media can fulfill this requirement. The development of such solvent-compatible DR dyes is therefore essential for extending waterless dyeing technologies to blended textiles, as illustrated in Fig. 2 (path B). This strategy provides a potential route toward industrial implementation to dramatically reduce water consumption, minimize wastewater generation, simplify dyeing sequences, and lower the overall environmental footprint of textile manufacturing while preserving color performance and process efficiency.^17^

The present review examines recent progress in the design, synthesis, and application of DR dyes for waterless dyeing technologies. Section 2 describes DR dye fundamentals, Section 3 reviews their synthesis and classification, Section 4 evaluates coloration platforms, Section 5 compares challenges and future directions, and Section 6 presents the conclusions.

## 2. Fundamentals of disperse-reactive (DR) dyes

### 2.1. Definition and hybrid molecular design

DR dyes represent a new class of hybrid colorants that combine the key structural features of traditional disperse dyes with chemically reactive functional groups.^18^ This dual-functional molecular design enables the dyes to exhibit both physical affinity toward hydrophobic fibers and the ability to form covalent bonds with specific functional groups on the fiber surface. As a result, DR dyes aim to overcome the inherent limitations of conventional disperse dyes, particularly their moderate wet fastness and limited fixation efficiency.^19^

Conventional disperse dyes are non-ionic, sparingly water-soluble colorants designed primarily for hydrophobic fibers such as polyester.^20^ Their fixation relies mainly on physical diffusion into the amorphous regions of the fiber at elevated temperatures, followed by entrapment within the polymer matrix.^21^ Although this mechanism provides good sublimation fastness, it does not involve chemical bonding, which can lead to dye migration, bleeding, or reduced wash fastness under certain conditions.^22^ Disperse dyes are commonly classified according to their chemical structure, application behavior, and thermal energy level. A summary of these classifications is presented in Table 1.

**Table 1:** Various classification schemes for disperse dyes^15,23^^a^

Classification basis	Category		Key characteristics
Chemical structure	Azo	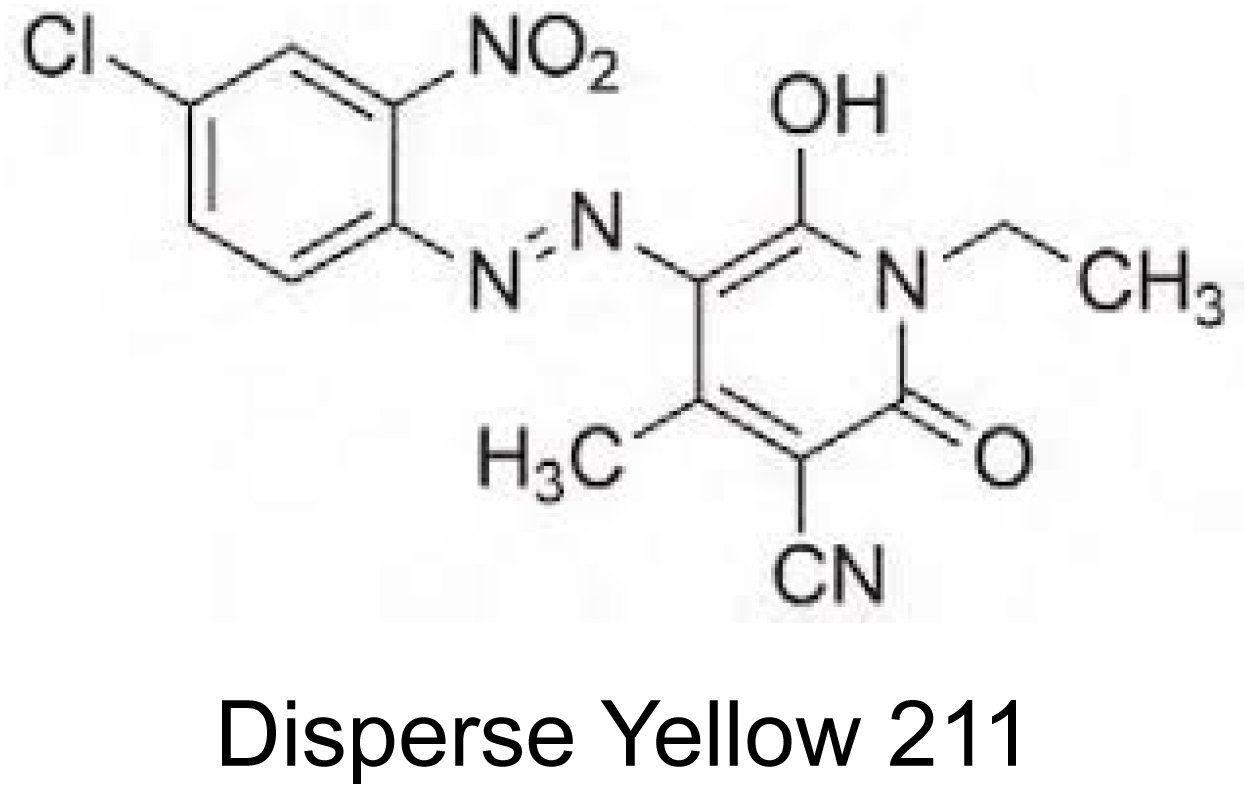	Largest class; includes monoazo and diazo dyes; good stability and low cost
	Anthraquinone	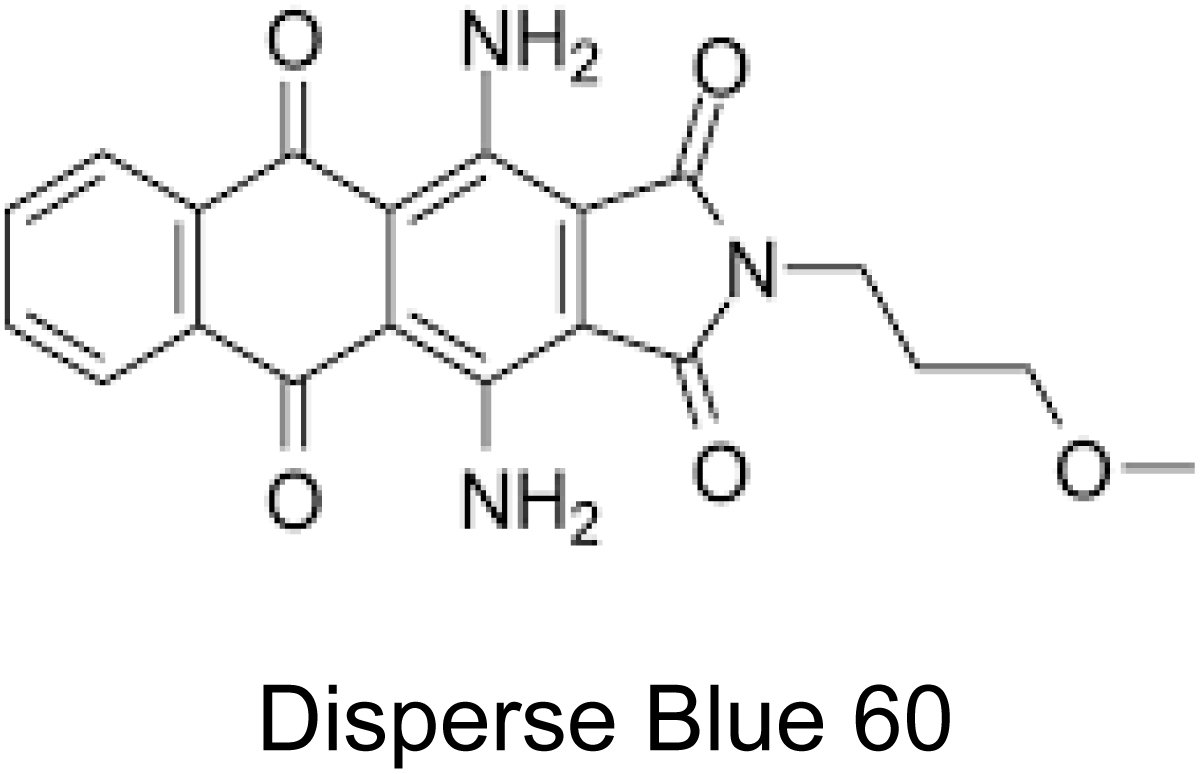	Bright blue and green shades; excellent light fastness
	Nitro	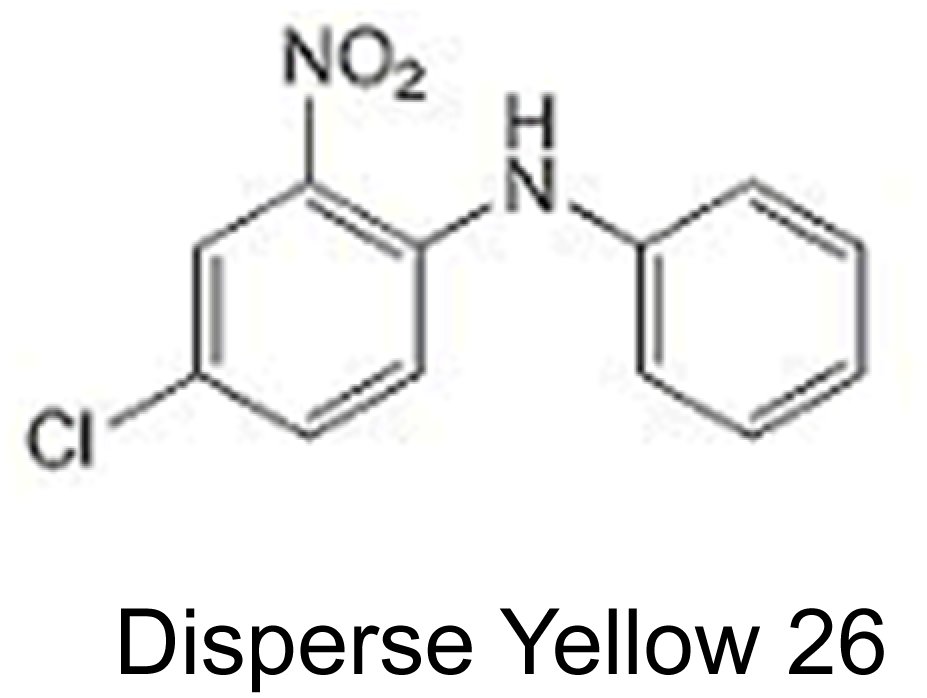	Mainly yellow shades; good fastness but limited applications
	Benzodifuran/benzodioxazole	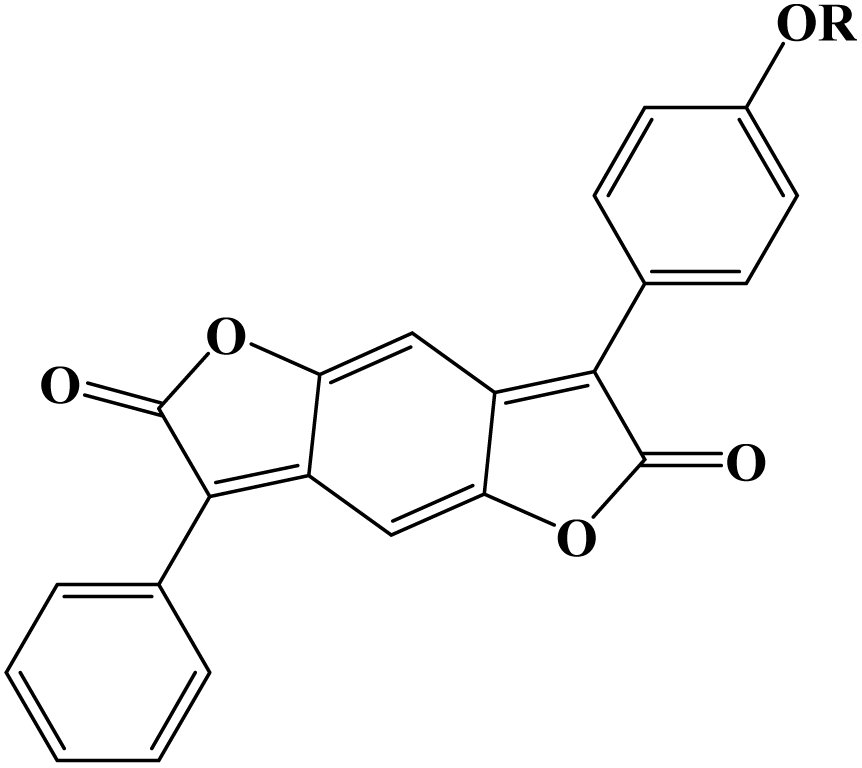	Strong color strength; used for special polyester applications
Application type	Type A		Low molecular weight; high dyeing rate; very poor sublimation fastness; mainly for acetate and nylon
	Type B		Medium sublimation fastness; good leveling; widely used for polyester, acetate, and nylon
	Type C		Good sublimation fastness; suitable for most polyester dyeing methods (carrier, HTHP, thermosol)
	Type D		Highest sublimation fastness; high molecular weight; mainly for HTHP and thermosol polyester dyeing
Thermal stability/energy level	Low energy (E)		Small, low-polarity molecules; fast dyeing and good leveling; poor heat and sublimation fastness; suitable for carrier dyeing
	Medium energy (SE)		Balanced leveling and sublimation resistance; suitable for polyester dyeing at medium temperatures
	High energy (S)		Higher molecular weight and polarity; slow dyeing rate; excellent thermal and sublimation fastness; used at high temperatures

a The “Type P” (discharge printing) and “Type RD” (rapid dyeing) are special-use modifiers, not primary classifications.

Reactive dyes are characterized by an electrophilic functional group capable of forming covalent bonds with nucleophilic sites on fibers, such as hydroxyl (–OH) groups in cellulosic fibers (*e.g.*, cotton) or amino (–NH_2_) groups in polyamide fibers.^24^ Common reactive groups include monochlorotriazine, vinyl sulfone, and difluorochloropyrimidine.^25^ The molecular architecture of a typical reactive dye consists of four key structural features, summarized in Fig. 3.

**Fig. 3:**
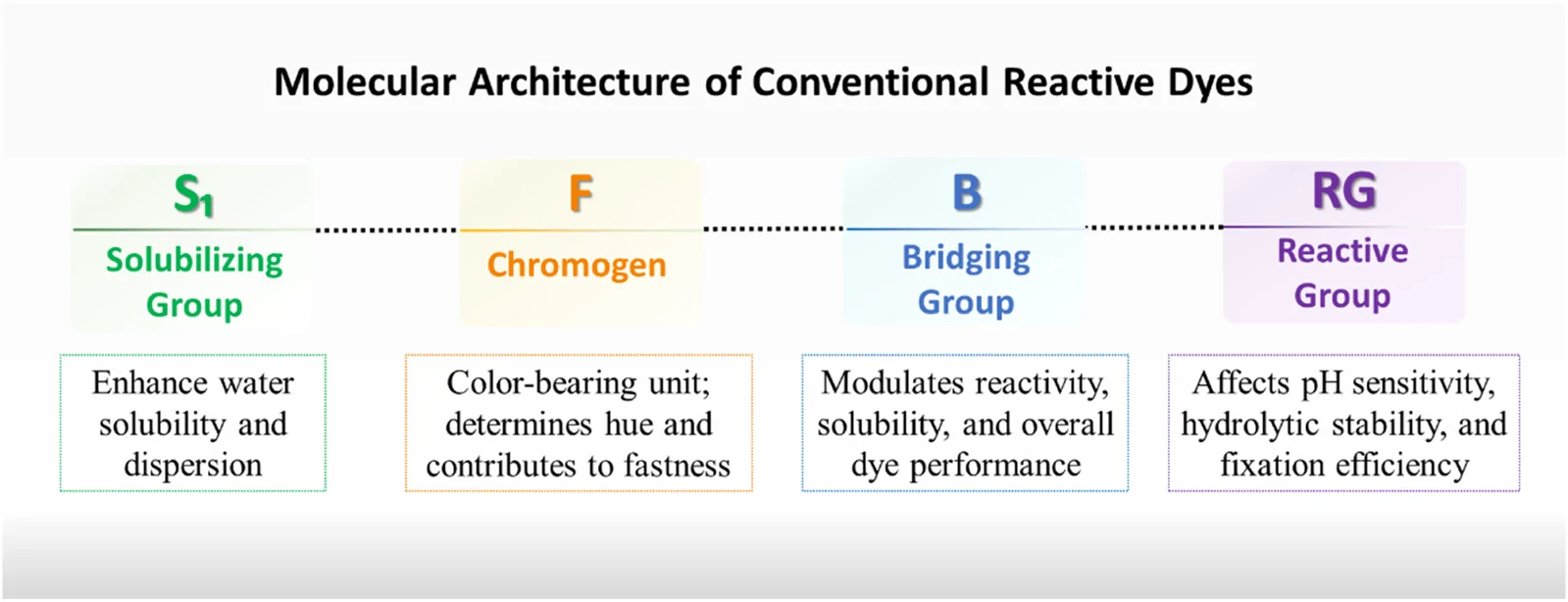
Molecular architecture of conventional reactive dyes.^25^

Reactive dyes contain various electrophilic reactive groups, which are responsible for covalent bonding with nucleophilic sites on fibers. Table 2 summarizes the major reactive moieties used in conventional reactive dyes, along with their typical subtypes.

**Table 2:** Classification of reactive groups in conventional reactive dyes

Reactive system	Subtypes/examples	
Cyanuric chloride (triazine group)	Dichlorotriazine-based reactive dyes	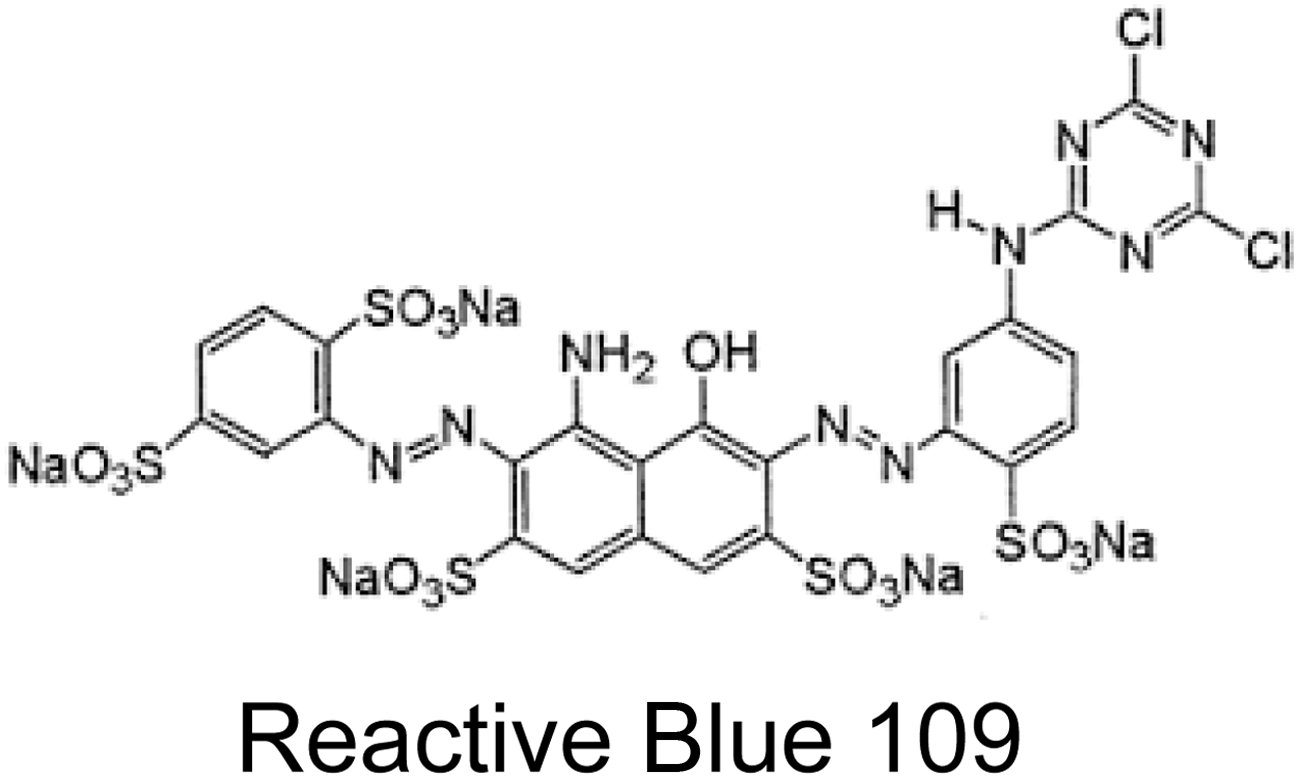
	Monochlorotriazine-based reactive dyes	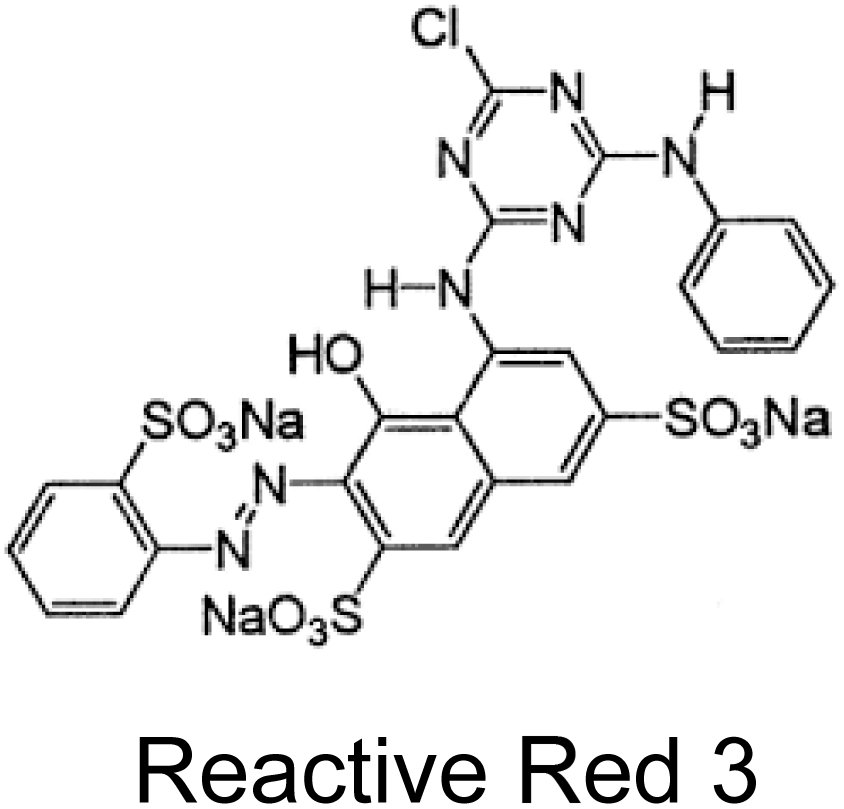
Pyrimidine	Trichloropyrimidinyl-based reactive dyes	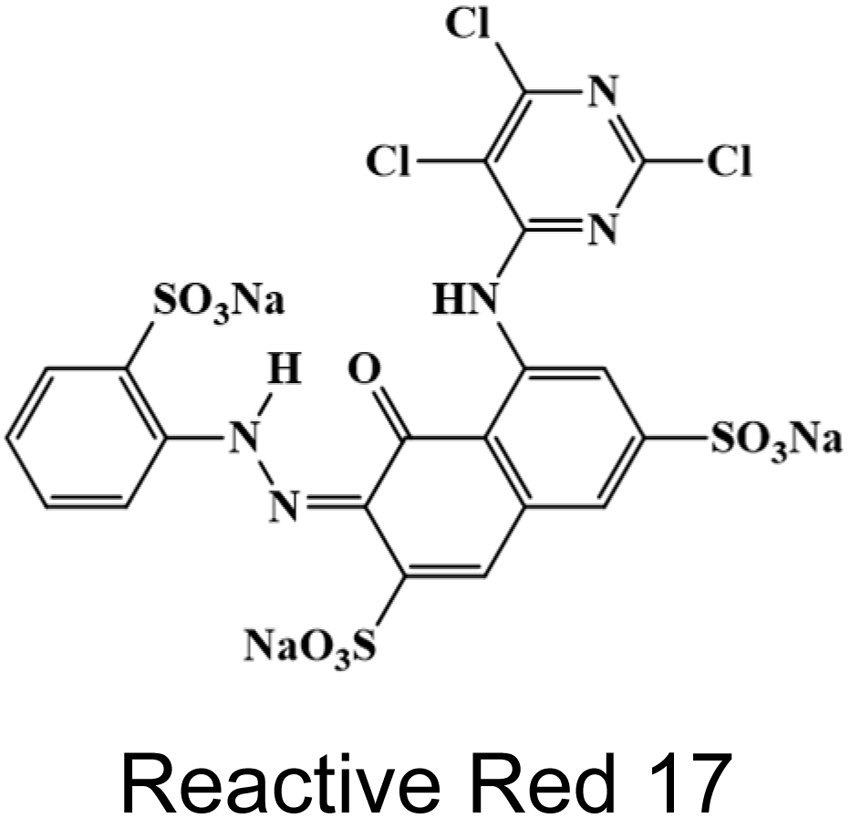
	Monochloro-difluoropyrimidinyl-based reactive dyes	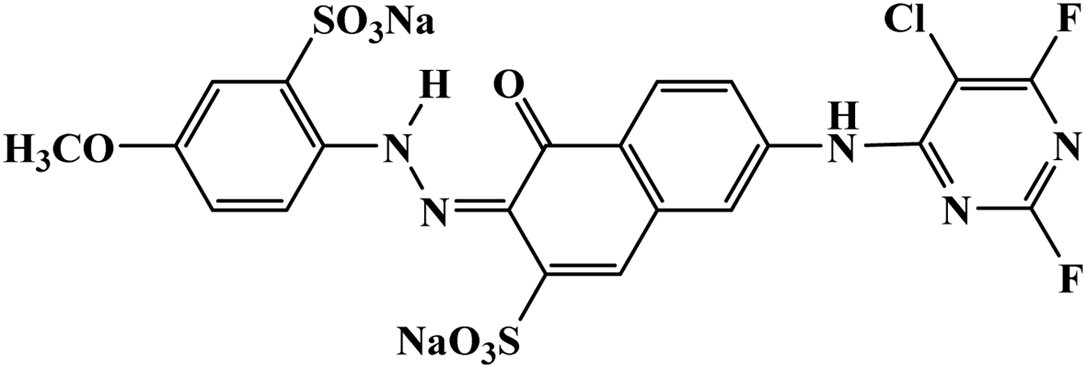
Dichloroquinoxaline		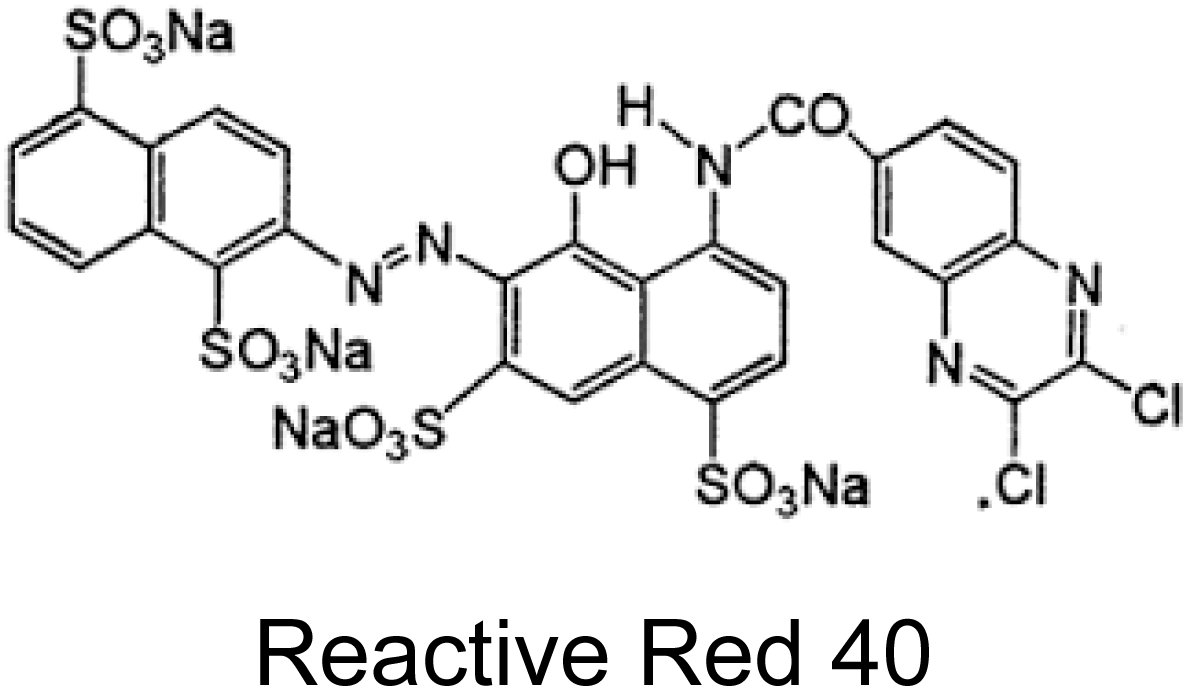
Bromoacrylamide		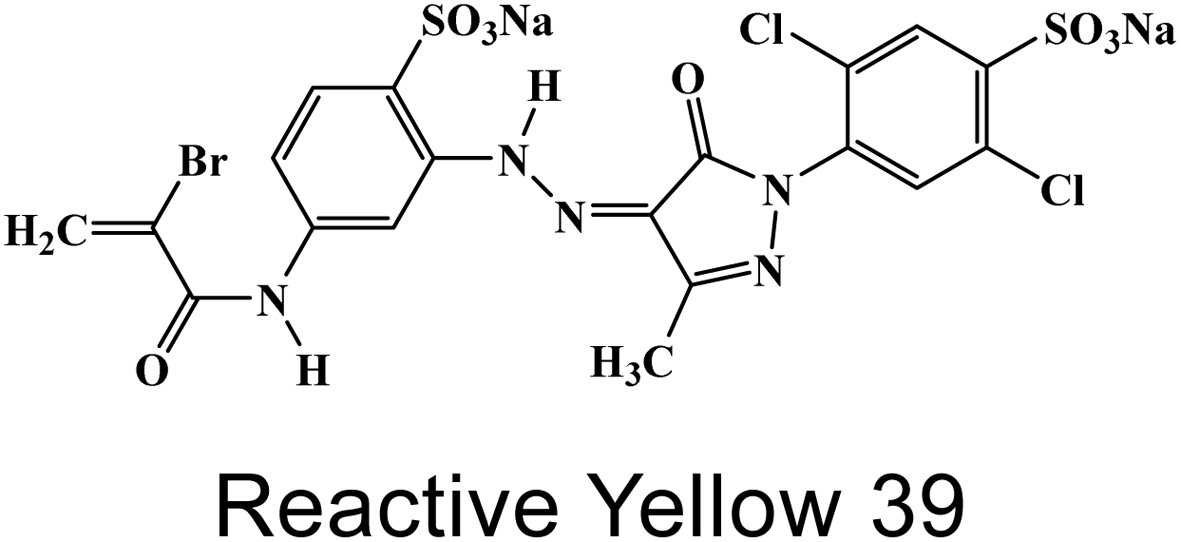
Phosphonic acid		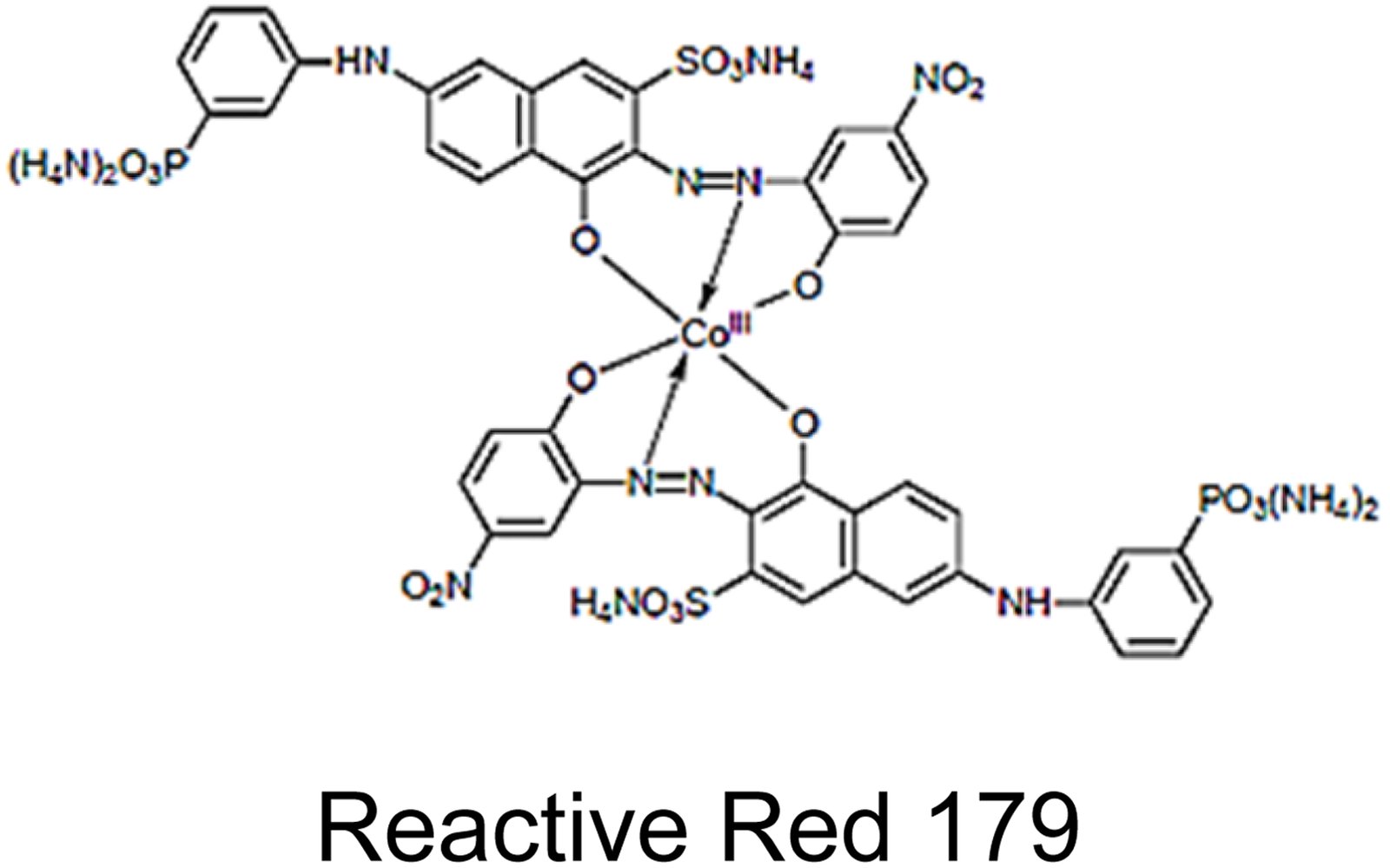
Sulfatoethylsulfone		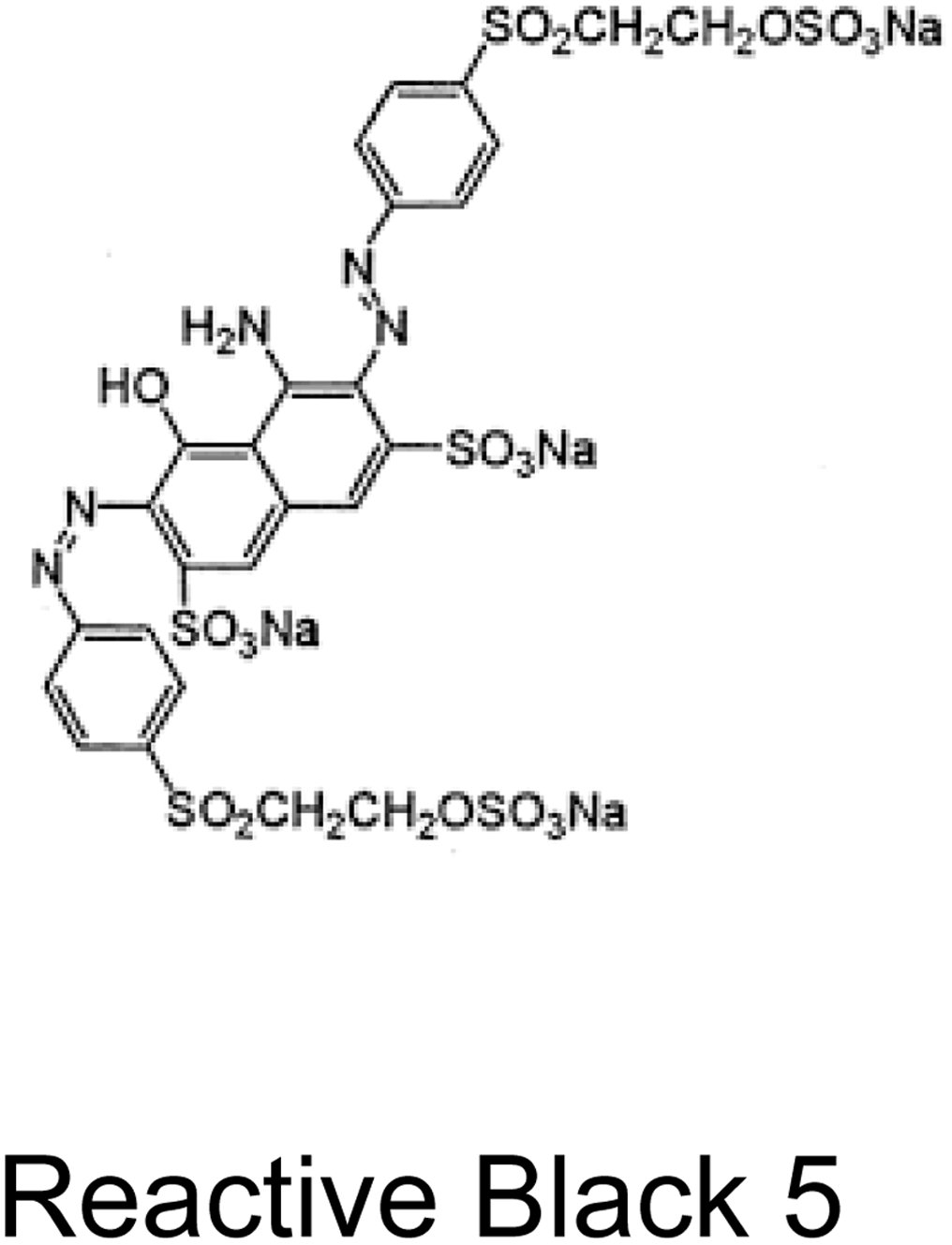

In contrast, DR dyes incorporate reactive moieties, such as halotriazine, vinyl sulfone, or other electrophilic functional groups, into the disperse dye chromophore.^26^ These reactive sites are capable of forming covalent bonds with nucleophilic groups present in fibers or in surface-modified polyester.^27^ The molecular structure of DRs should consist of three essential components: a chromophore, a linking group, and a reactive group.^28^

This hybrid molecular architecture is designed to balance two competing requirements: adequate hydrophobicity for diffusion into synthetic fibers and sufficient chemical reactivity for covalent fixation.^13,14,29^ If the dye is too hydrophilic, its disperse-dye character is lost; if it is too hydrophobic, the reactive group may become inaccessible or ineffective. Therefore, careful control of molecular size, polarity, and substituent positioning is essential in the design of effective DR dyes.^18^

The concept of DR dyes is closely related to the development of high-performance, environmentally conscious dyeing systems. By promoting higher fixation rates and reducing unfixed dye in the effluent, these hybrid dyes have the potential to lower water consumption, decrease chemical usage, and improve overall process sustainability.^30^ Their design is particularly relevant in advanced dyeing techniques, including low-liquor-ratio processes, SC-CO_2_ dyeing, and waterless or near-waterless technologies.^26^

Overall, DR dyes represent a breakthrough in dye chemistry, integrating the diffusion-based mechanism of disperse dyes with the covalent fixation concept of reactive dyes to achieve improved fastness and more sustainable dyeing performance.

### 2.2. Dyeing mechanism of DR dyes

The dyeing mechanism of DR dyes proceeds through a three-stage process involving initial diffusion, activation of the reactive moiety, and final covalent bonding with fiber functional groups (Table 3).^31–33^

**Table 3:** Mechanistic steps of DR dyeing on fibers^31–33^

Stage	Mechanism step	Key process	Outcome
Stage 1	Physical adsorption and diffusion	DR dye molecules are dispersed in the dye bath, adsorb onto the fiber surface, and diffuse into the amorphous regions of both hydrophilic and hydrophobic fibers	Physical penetration of dye into the fiber matrix
Stage 2	Activation of the reactive group	Heat, moisture, or alkaline conditions activate the reactive functional group, increasing its susceptibility to nucleophilic attack	Formation of an active electrophilic site on the dye molecule
Stage 3	Covalent bond formation	The activated reactive group reacts with nucleophilic functional groups on the fiber or modified surface	Stable covalent dye–fiber bond and improved wash fastness

### 2.3. Factors influencing dyeing efficiency

The efficiency of dyeing with DR dyes depends on several related factors that affect physical diffusion and covalent fixation.^25,29,34–37^ In general, the effectiveness of DR dyeing arises from the combined influence of fiber structure, dye molecular design, and dyeing conditions, all of which govern dye transport, adsorption, and covalent fixation within the fiber matrix. These factors determine the extent of dye uptake, fixation efficiency, and final color uniformity. A clear understanding of these interconnected parameters is essential for optimizing dyeing processes, particularly in emerging solvent-assisted or waterless systems. The key variables influencing DR dyeing efficiency are summarized in Table 4.

**Table 4:** Summary of key factors affecting dyeing efficiency^25,29,34–37^

Factor	Effect on dyeing	Notes
Fiber properties	Influences diffusion and accessibility of reactive sites	Higher amorphous content → better uptake
Dye structure	Determines solubility, diffusion rate, and fixation efficiency	Smaller, less polar molecules diffuse faster; reactive group type critical
Temperature	Enhances diffusion and reactive group activation	Excessive heat → hydrolysis risk
pH/alkali	Activates reactive groups; affects nucleophilicity of fibers	Optimal pH required for each dye class
Time	Longer exposure increases fixation	Excessive time → hydrolysis of reactive moieties
Carriers/auxiliaries	Improve leveling, penetration, and dispersion	Common for polyester blends
Water quality/ionic strength	Affects solubility, adsorption, and evenness	Hard water may reduce dye uptake

## 3. Review of the synthesis of DR dyes

Across the wide range of synthetic approaches reported for DR dyes in the literature, a common and fundamental design principle prevails: the integration of a chromophoric color-generating unit with a reactive functional group. Most synthetic routes follow a principal strategy. In the more common approach, the chromophore structure (such as azo, anthraquinone, thiazole, or pyrrole–pyrazolone systems) is first constructed, after which the reactive group, typically a chlorotriazine or vinyl sulfone moiety, is introduced in a separate step onto a suitable functional site of the chromophore. Key factors for the success of these syntheses include the appropriate choice of solvent (ranging from acetone and dioxane to DMF and toluene), precise temperature control (particularly for reactions involving cyanuric chloride at 0–5 °C), and the use of suitable bases (such as sodium carbonate, triethylamine, or sodium acetate) to neutralize the generated acid. Finally, common purification techniques, including recrystallization, column chromatography, and desalting, play a critical role in isolating and purifying the final products. DR dyes have unique physicochemical properties resulting from the combination of a hydrophobic chromophore and an electrophilic reactive group that control dye solubility, diffusion, fiber interaction, and fixation efficiency.^38–41^

The dyeing performance of DR dyes depends critically on their physicochemical properties. Low molecular weight enhances diffusion into hydrophobic fibers (*e.g.*, polyester), while high molecular weight improves fixation and light fastness. The balance between hydrophobic chromophores and hydrophilic sulfonate groups ensures good dispersion and leveling. The reactive group (*e.g.*, triazine, vinyl sulfone) governs covalent fixation, pH sensitivity, and hydrolysis resistance. Solubility in supercritical CO_2_ enables waterless dyeing, while thermal stability allows processing up to 150 °C. Finally, DR dyes offer a broad color gamut and excellent fastness properties (wash 4–5, light 5–6, and satisfactory rub fastness) (Table 5).^38–41^

**Table 5:** Physicochemical properties of DR dyes and their effects on dyeing performance^38–41^

Property	Description/relevance	Effect on dyeing
Molecular weight	Size of dye molecule	Low MW → faster diffusion into hydrophobic fibers; high MW → slower diffusion but better fixation and light fastness
Polarity/hydrophobicity	Balance between hydrophobic chromophore and hydrophilic substituents (*e.g.*, sulfonates)	Hydrophobic chromophores penetrate polyester; hydrophilic groups improve dispersion and leveling
Reactive group	Electrophilic moiety (*e.g.*, triazine, pyrimidine, vinyl sulfone)	Governs covalent bond formation, fixation efficiency, pH sensitivity, and hydrolysis susceptibility
Solubility (non-aqueous/SC-CO_2_)	Dissolution in non-aqueous media such as SC-CO_2_	Facilitates waterless dyeing; disperse moiety ensures solubility, reactive moiety enables fiber fixation
Thermal stability	Resistance to high-temperature conditions	Essential for high-temperature polyester dyeing (up to 150 °C); reduces thermal decomposition during processing
Color gamut	Range of achievable shades	Enables broad color spectrum: yellow, red, blue, black
Fastness properties	Wash, light, and rub fastness	Fastness varies with dye structure, substrate, and conditions; representative results are reported in the application tables

### 3.1. Overview of synthetic routes to DR dyes

The following sections review the reported studies on the synthesis of DR dyes:

The synthesis of dye DR1 is accomplished in two main stages (Scheme 1). In the first stage, an amino-functionalized heterocyclic precursor (2-amino-4-phenylthiazole) is prepared *via* the cyclization of acetophenone with thiourea in the presence of iodine in toluene under thermal conditions. Following purification, this compound (1) serves as the chromophore building block for the final dyes. In the second stage, DR dyes are constructed. Initially, cyanuric chloride reacts with aniline to form an intermediate dichlorotriazine containing one reactive site (2). Subsequently, this intermediate is coupled under mild, low-temperature conditions with the azo-thiazole (1) precursor *via* nucleophilic substitution. Consequently, dye DR1 is produced, which contains a reactive chlorotriazine group capable of forming covalent bonds with hydroxyl groups on cellulose fibers.^42^

**Scheme 1:**
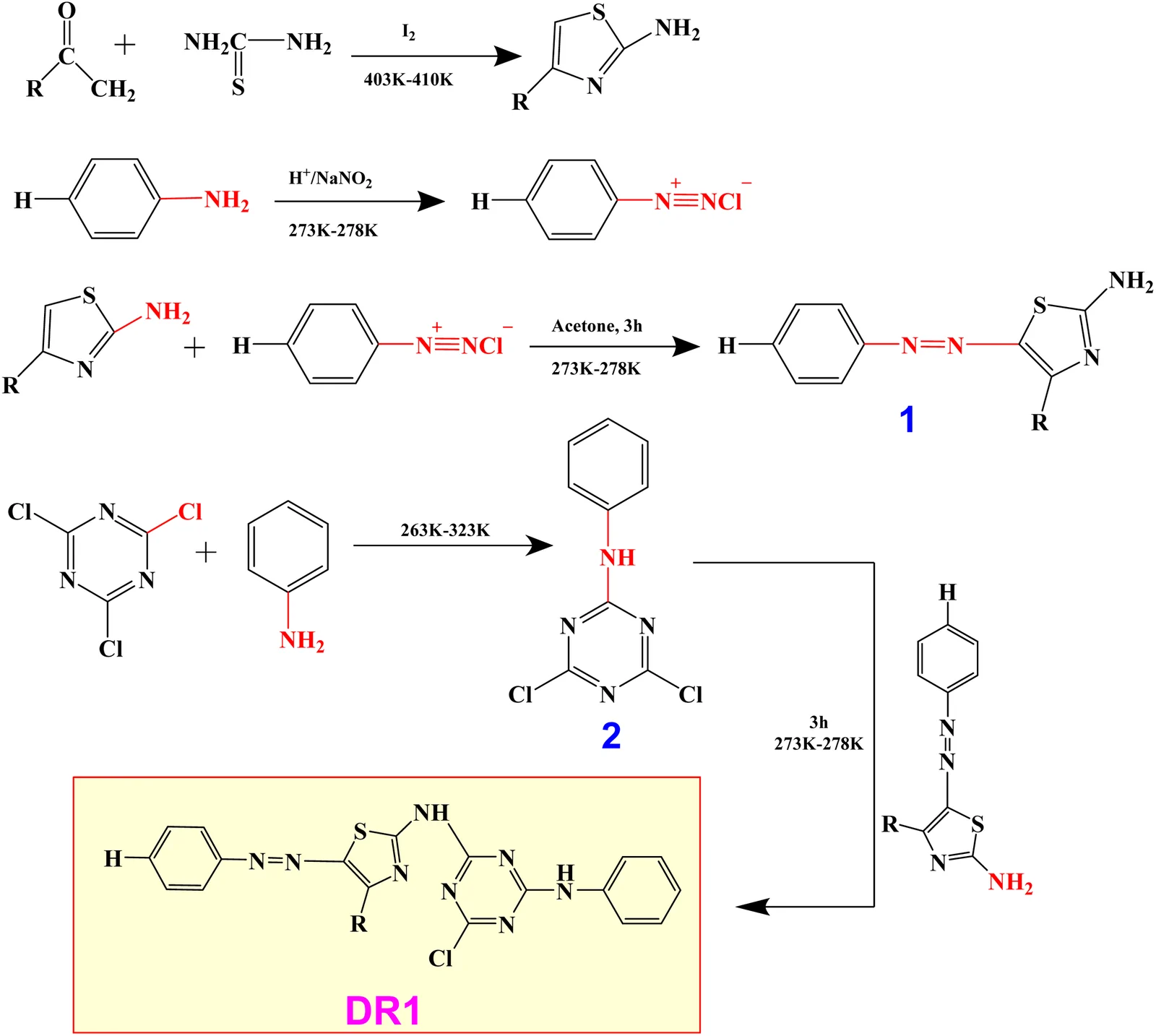
Synthesis of DR1 dye *via* thiazole precursor formation and subsequent coupling with dichlorotriazine.^42^

In the synthesis of DR2, these dyes follow a common structural strategy with different chromophores (Scheme 2). This pathway begins with the preparation of a shared diethylaniline intermediate (3), obtained by acidic hydrolysis of the corresponding acetamide precursor in ethanol. Next, the reactive triazine core is formed: cyanuric chloride reacts with amine (3) at 5 °C in toluene for 1 hour to produce a dichlorotriazine intermediate (4), and the reaction is stopped at this stage by immediate filtration, which isolates the intermediate as a gray filter cake without further substitution of the remaining chlorine atoms. Subsequently, an azo coupling reaction is carried out to generate the chromophore. The diazonium salt of *p*-nitroaniline is then coupled with intermediate (4) to form the azo compound (5). In the final stage, silane functionalization is performed. The azo compound (5) reacts with an aminosilane (*e.g.*, 3-aminopropyldimethoxymethylsilane) in tetrahydrofuran at 30 °C for 0.5 hours, where the remaining reactive chlorine atoms on the triazine ring is substituted by the aminosilane group. The final dye is collected by filtration, dried, and then purified by column chromatography using dichloromethane : ethyl acetate (10 : 1, v/v), yielding the final dyes (DR2, yield 63%).^43^

**Scheme 2:**
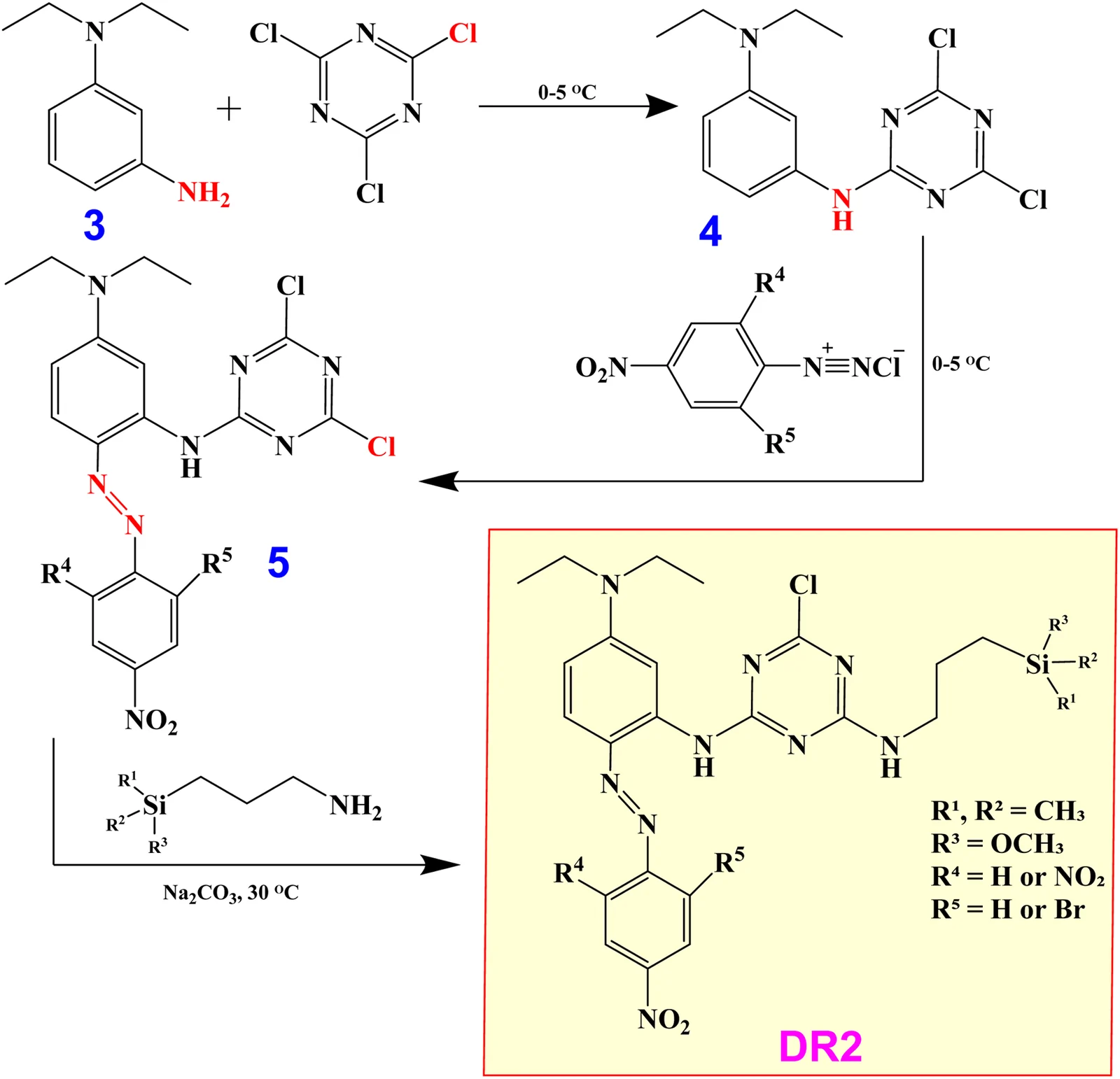
Synthesis of DR2 dyes *via* triazine formation, azo coupling, and silane functionalization.^43^

The synthesis of dye DR3 is based on the acylation of an aromatic amine containing a chromophore with chloro- or bromoacetyl chloride, with the aim of introducing a reactive haloacyl functional group into the chromophore structure (Scheme 3). For azo dyes (*e.g.*, DR3a, DR3b), aromatic azo amines—4-phenylazoaniline (6) and 4-nitrophenylazoaniline (7) react with haloacyl chloride in dichloromethane in the presence of triethylamine at low temperature. By combining two types of amines with two types of haloacyl chloride, four differently colored products are obtained. For the anthraquinone dye (DR3c), 1-amino-9,10-anthraquinone (8) is used as the starting material. Due to its different solubility, the reaction is carried out in DMF under similar temperature conditions. Upon completion, the product is precipitated by adding a large volume of water, then isolated, yielding two yellow powdered products. Overall, this is a simple and efficient one-step method for producing a family of azo and anthraquinone dyes, all containing a haloacetamide group (chloro- or bromoacetamide) as a reactive moiety or potential linker. (DR3a: yield 86%, DR3b: yield 83%, DR3c: yield 82%).^44^

**Scheme 3:**
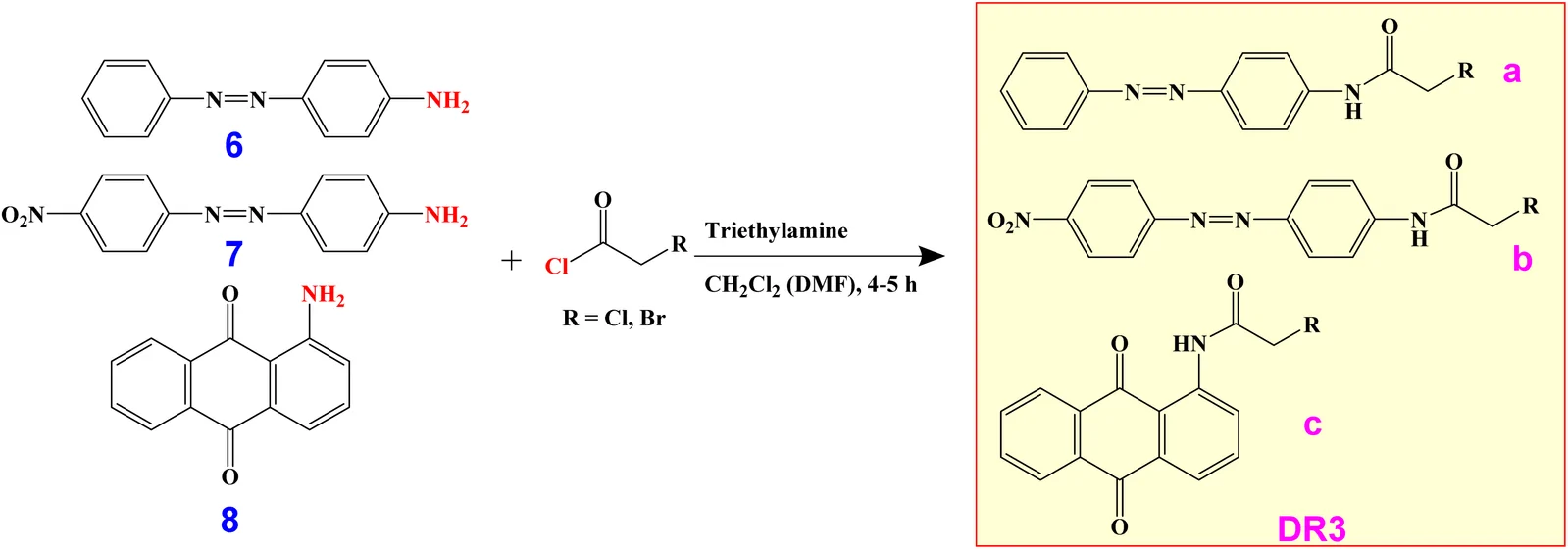
Synthesis of DR3 dyes *via* acylation of aromatic azo or anthraquinone amines with haloacetyl chlorides.^44^

The synthesis of dye DR4 follows a two-step pathway starting from an amine-containing dye core (9) (Scheme 4). In the first step, the hydroxyethyl groups of compound (9) undergo esterification with phenylacetyl chloride in dichloromethane at low temperature (approximately 0 °C) in the presence of triethylamine, yielding a phenylacetate intermediate (10). In the second step, this intermediate undergoes a Regitz diazo transfer reaction using 4-acetamidobenzenesulfonyl azide (*p*-ABSA) in the presence of DBU in acetonitrile. This converts the phenylacetate groups into α-phenyl diazo ester groups, yielding the final DR4 dyes (yield 85%). The resulting dyes contain a reactive diazo ester functionality capable of forming covalent bonds with fibers *via* carbene insertion upon heating. Structural variations in compound (9) lead to the formation of five different final derivatives.^45^

**Scheme 4:**
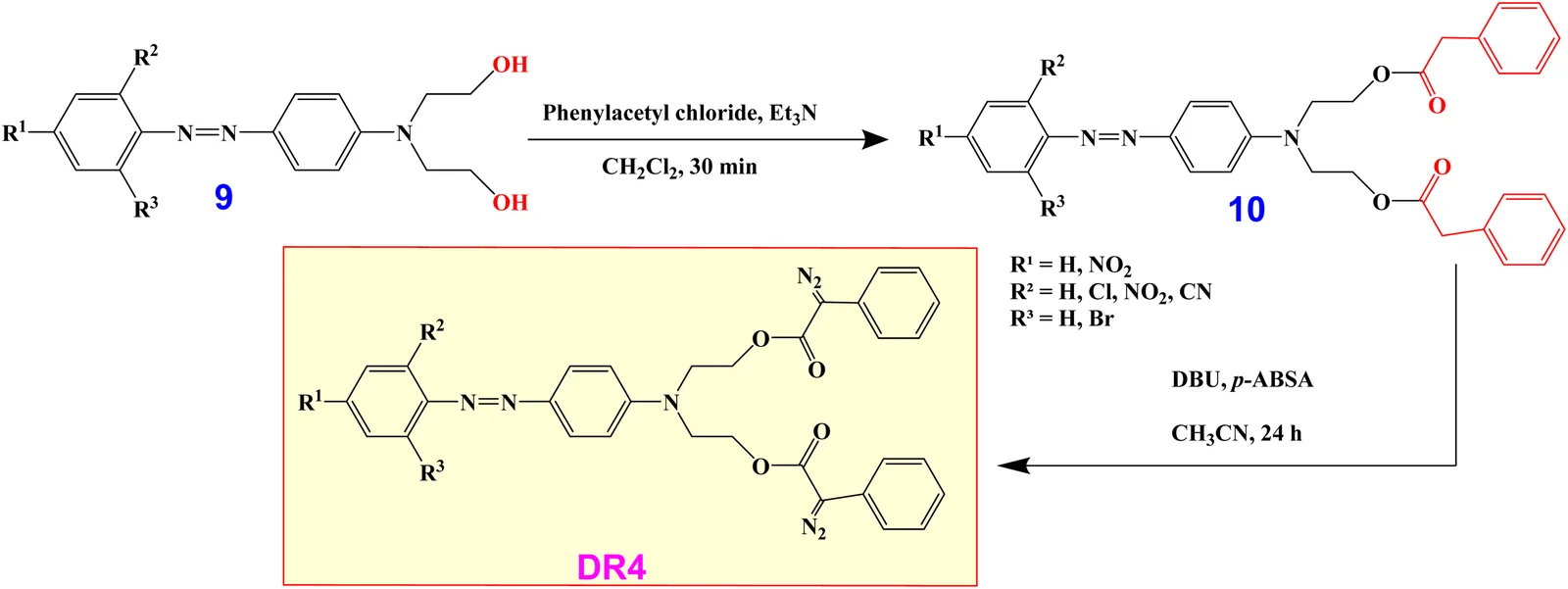
Synthesis of DR4 dyes *via* acylation and DBU-mediated substitution to form a covalent bis-chromophoric system.^45^

This synthetic pathway, as shown in Scheme 5, presents a rational multi-step strategy for preparing DR5 dyes based on an anthraquinone (quinizarin) core. The overall objective is to attach a reactive triazine unit, derived from cyanuric chloride, to an anthraquinone chromophore functionalized with an aminoalkyl chain. The quinizarin chromophore (11) undergoes a nucleophilic aromatic substitution (SNAr) reaction with a primary amine. This step directly introduces an aminoalkyl group onto the dye core. The free hydroxyl group on the chromophore is protected with a sulfonyl (tosyl) group. This serves two purposes: it prevents interference in subsequent steps and converts the hydroxyl into a good leaving group for the next reaction. The tosyl group in compound (12) is then replaced *via* another substitution reaction with a protected aminoalkyl carbamate. This step attaches an aminoalkyl chain of variable length to the chromophore while the terminal amine remains protected. The Boc protecting group at the chain terminus is removed under mild acidic conditions, liberating a free primary amine (13) essential for the final step. The amine obtained by deprotecting intermediate (13) reacts with cyanuric chloride *via* an SNAr reaction at low temperature (approximately 0 °C). This step connects the reactive chlorotriazine group to the dye through an aminotriazine C–N linkage, yielding the final DR5 dye (yield 53%).^33^

**Scheme 5:**
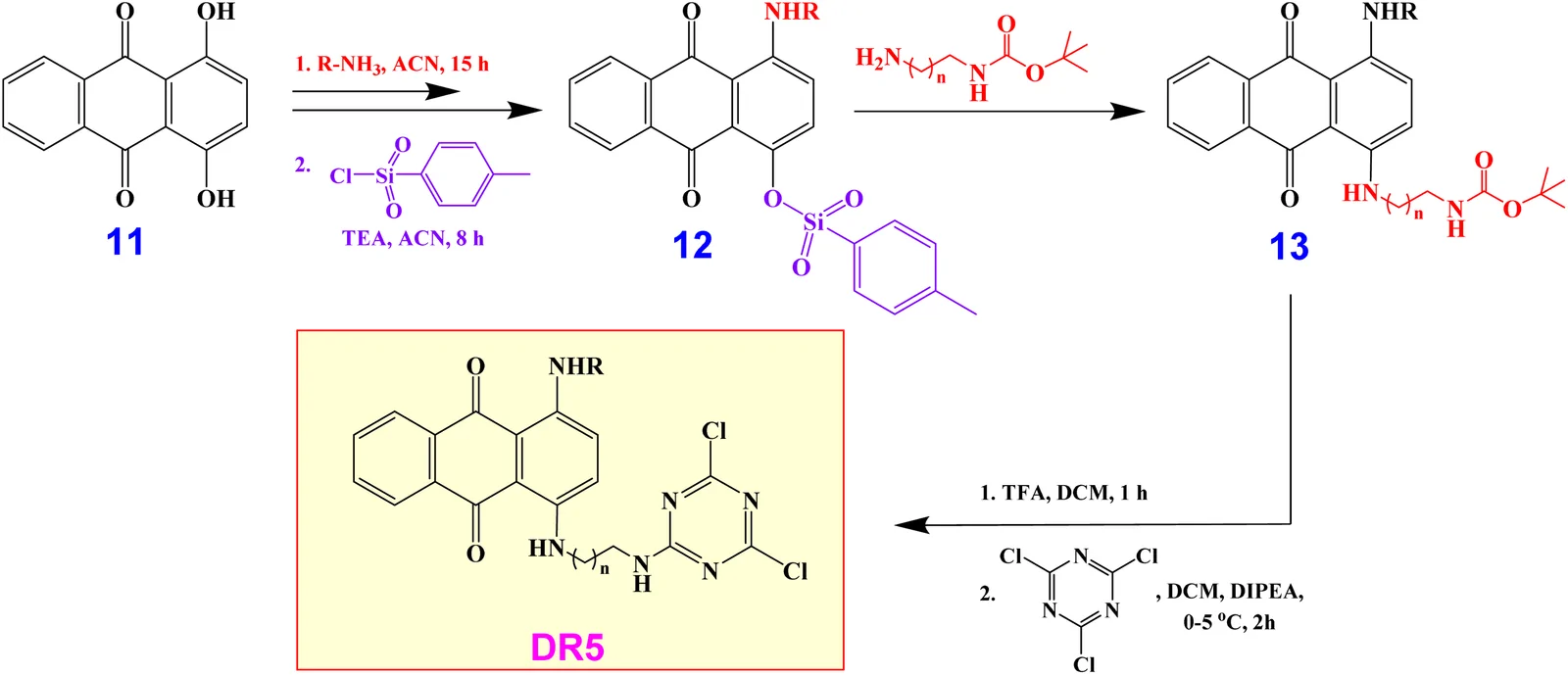
Multi-step synthesis of DR5 dyes *via* quinizarin functionalization, tosyl protection, Boc-amine coupling, and cyanuric chloride condensation.^33^

In the synthesis of dye DR6, as indicated in Scheme 6, the overall strategy involves linking an anthraquinone chromophore to a reactive triazine core. The first step constructs the core chromophore *via* a C–N coupling reaction of the Ullmann type between the anthraquinone core and a benzene ring. Specifically, 1-chloroanthraquinone (14) reacts with a sulfate salt of diaminotoluene (2,5-diaminotoluene) (15) in the presence of copper(i) iodide as a catalyst and potassium carbonate as a base, in DMF under a nitrogen atmosphere at 100 °C. The product (16) retains a free amino group (–NH_2_) for the subsequent step. In the second step, the aminoanthraquinone precursors undergo a condensation reaction with cyanuric chloride. This reaction is carried out at 0–5 °C in the presence of a mild aqueous base to neutralize the acid released, using 1,4-dioxane as the solvent. Under these conditions, only one of the reactive chlorine atoms of cyanuric chloride is substituted by the primary amine, attaching a dichlorotriazine unit to the chromophore. This transformation yields the final DR6 dyes (yield 74%), in which the two remaining reactive chlorine atoms on the triazine ring enable covalent bonding with fibers.^46^

**Scheme 6:**
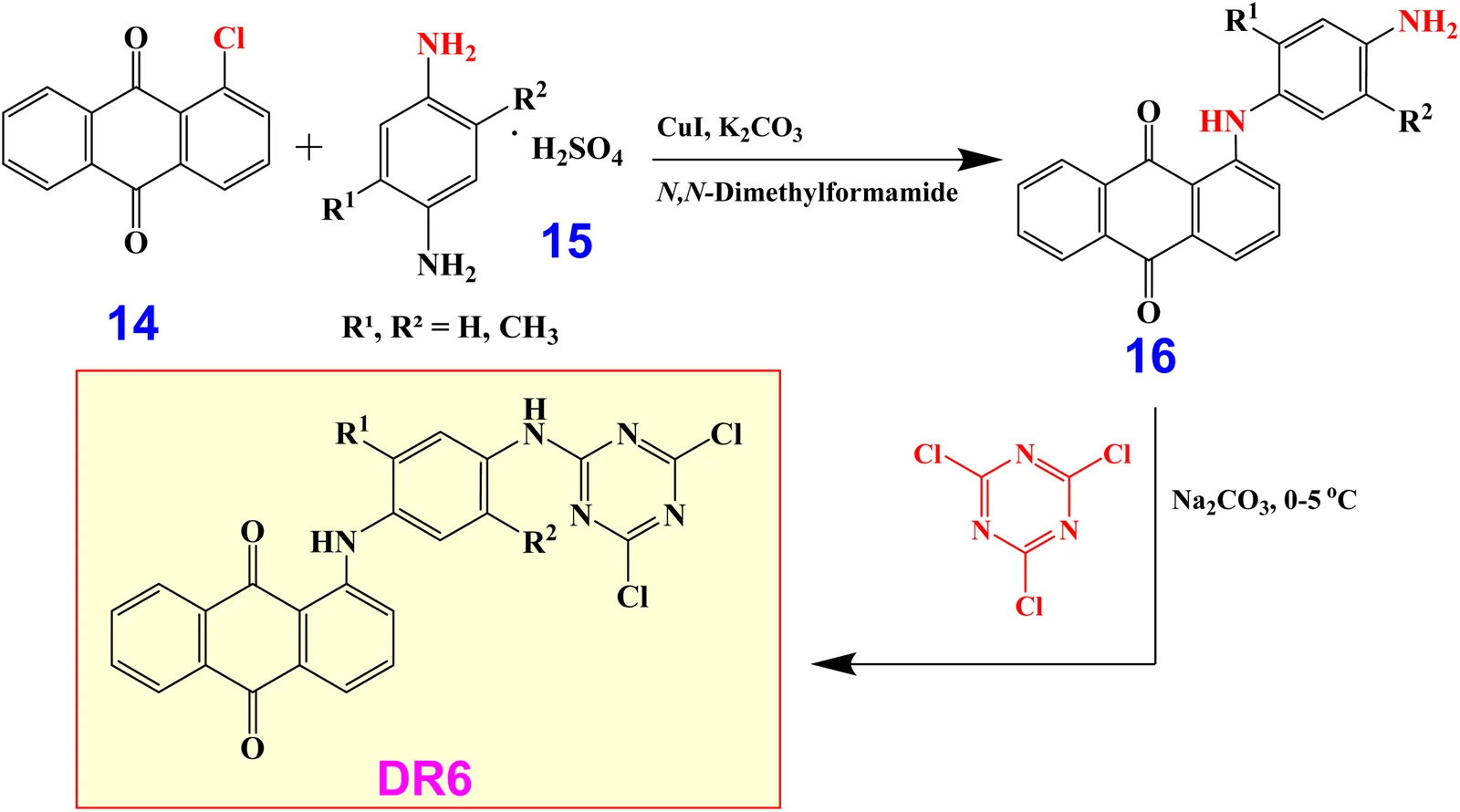
Synthesis of DR6 dyes *via* Ullmann C–N coupling and dichlorotriazine condensation.^46^

In the synthesis of dye DR7, a two-step pathway is followed in which a more complex chromophore is first formed and then converted into the final dye (Scheme 7). 1,4-Dihydroxyanthraquinone reacts with ethylenediamine and aniline in isobutanol under staged heating under nitrogen protection. The reaction is first stirred at 60 °C, then heated to 108 °C for 3 hours, and after cooling to 95 °C, copper acetate and piperidine are added, followed by boiling under an oxygen atmosphere for 3 hours. This oxidative condensation produces a hybrid anthraquinone structure (17) containing both aminoaryl and aminoalkyl functionalities. The yield of this precursor (17) is 67.5% after purification. Purification is performed by column chromatography using dichloromethane : petroleum ether (3 : 1, v/v) as eluent, followed by washing with acetone. The complex anthraquinone precursor (17) is then condensed with cyanuric chloride in 1,4-dioxane at 1–5 °C under mild basic conditions (sodium carbonate) for 3 hours. After the reaction, deionized water is added, and the precipitated product is isolated by filtration, washed, and dried under vacuum. Further purification is performed by column chromatography using dichloromethane : petroleum ether (1 : 1, v/v). The final product is DR7 in an isolated yield of 63%, containing a triazine core with two remaining chlorine groups, which may contribute to interactions with fibers.^47^

**Scheme 7:**
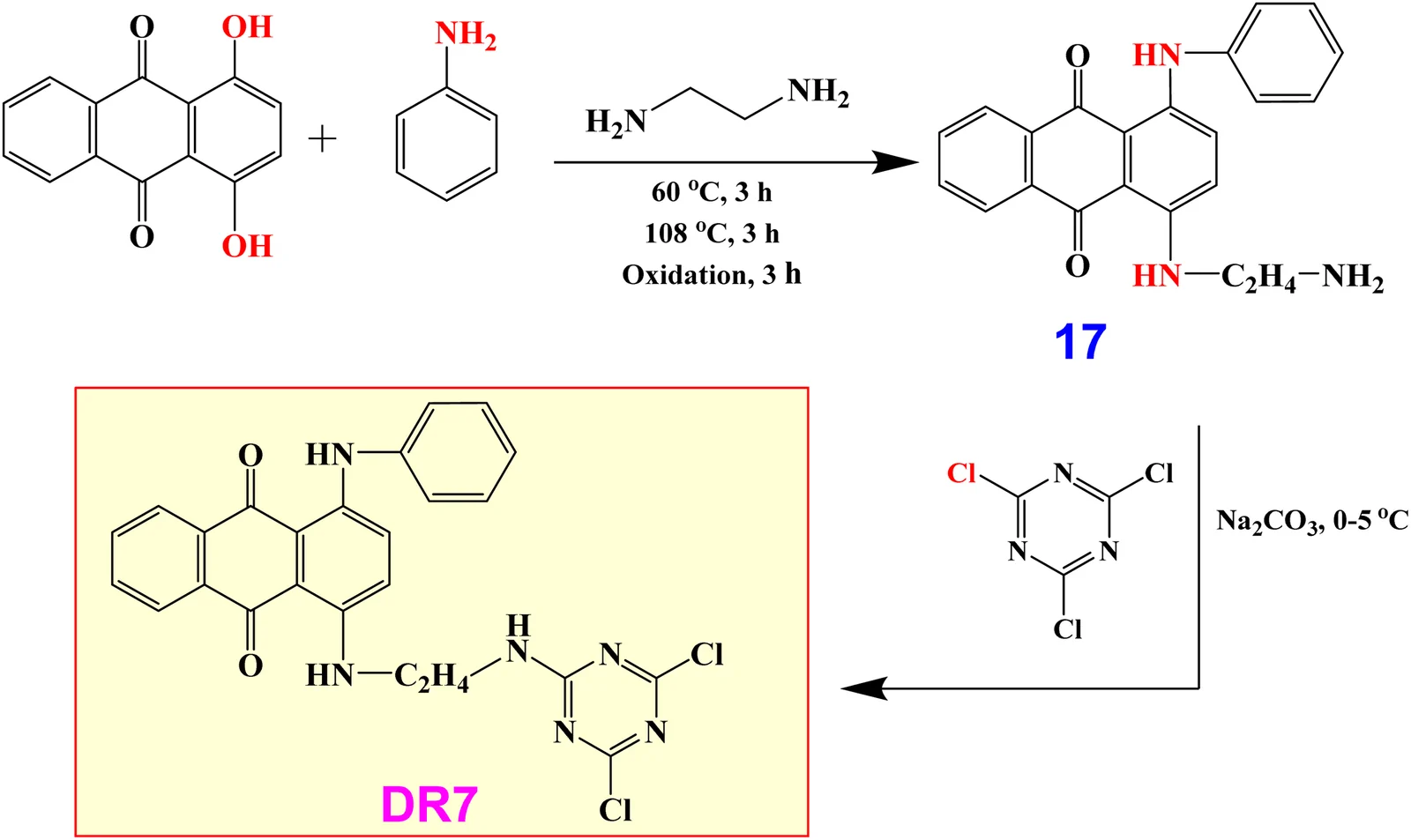
Synthesis of DR7 dyes *via* oxidative condensation of 1,4-dihydroxyanthraquinone with ethylenediamine and aniline, followed by triazine functionalization.^47^

This route follows a clear two-step strategy for preparing dye DR8, which is distinguished by the use of a flexible bridging group between the chromophore and the reactive unit (Scheme 8). 1-Chloroanthraquinone undergoes a Ullmann-type C–N coupling with *N*-phenylethylenediamine in the presence of copper metal and potassium hydroxide in DMF under nitrogen. This reaction attaches the anthraquinone core to one end of a diamine chain, yielding product (18), while a secondary amine group remains at the opposite terminus. The ethylenediamine segment serves as a flexible spacer between the chromophore and the reactive group. The secondary amine on the spacer reacts with cyanuric chloride in 1,4-dioxane at low temperature in the presence of sodium carbonate. One reactive chlorine atom is substituted, forming an amide bond and attaching a dichlorotriazine unit to the end of the chain. The final dye DR8 (yield 60%) consists of an anthraquinone chromophore, a flexible ethylenediamine bridge, and a reactive chlorotriazine group.^48^

**Scheme 8:**
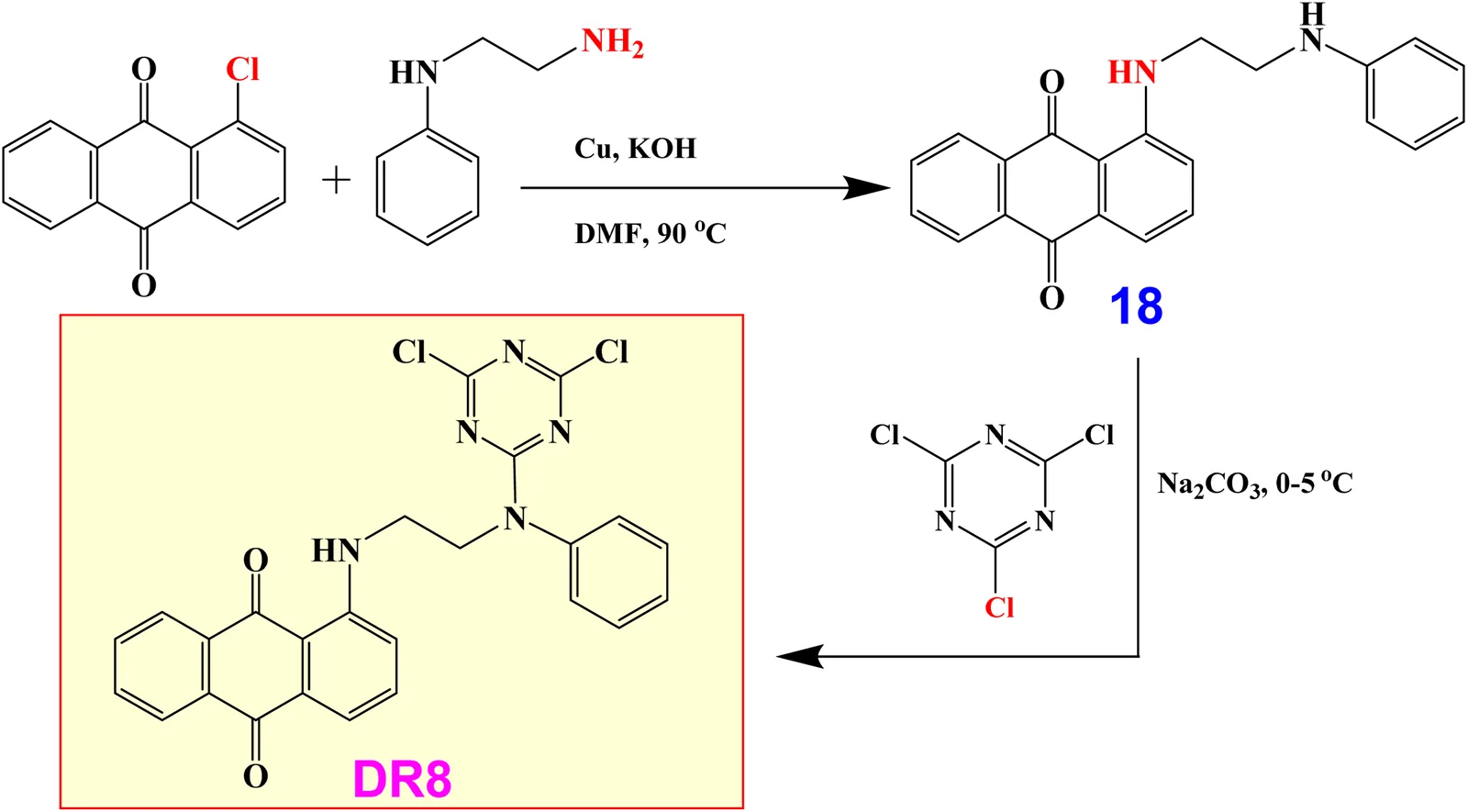
Synthesis of DR8 dye *via* Ullmann coupling and flexible ethylenediamine-linked dichlorotriazine attachment.^48^

Scheme 9 illustrates a two-step synthesis for producing a DR9 based on a tetrahydrobenzothiophene core, utilizing an unconventional hydrazine formation followed by azo coupling. An aminothiophene ester (19) is first diazotized with sodium nitrite under acidic conditions to generate an unstable diazonium intermediate. Instead of proceeding with a conventional azo coupling, this intermediate reacts directly with malononitrile in alkaline ethanolic solution. This yields a hydrazone structure (20) attached to the thiophene ring, containing a reactive hydrazone group (–N–N=C(CN)_2_) and a conjugated malononitrile unit. A reactive diazonium salt is prepared from a sulfonated amine precursor (21), introducing an ethylsulfate group that serves as a vinyl sulfone-type reactive moiety. This diazonium compound is then coupled with the hydrazino intermediate. The final product is dye DR9 (yield 70%), which possesses a complex thiophene-hydrazino-malononitrile chromophore and a reactive vinyl sulfone group, enabling covalent bonding with cellulosic fibers under alkaline conditions.^49^

**Scheme 9:**
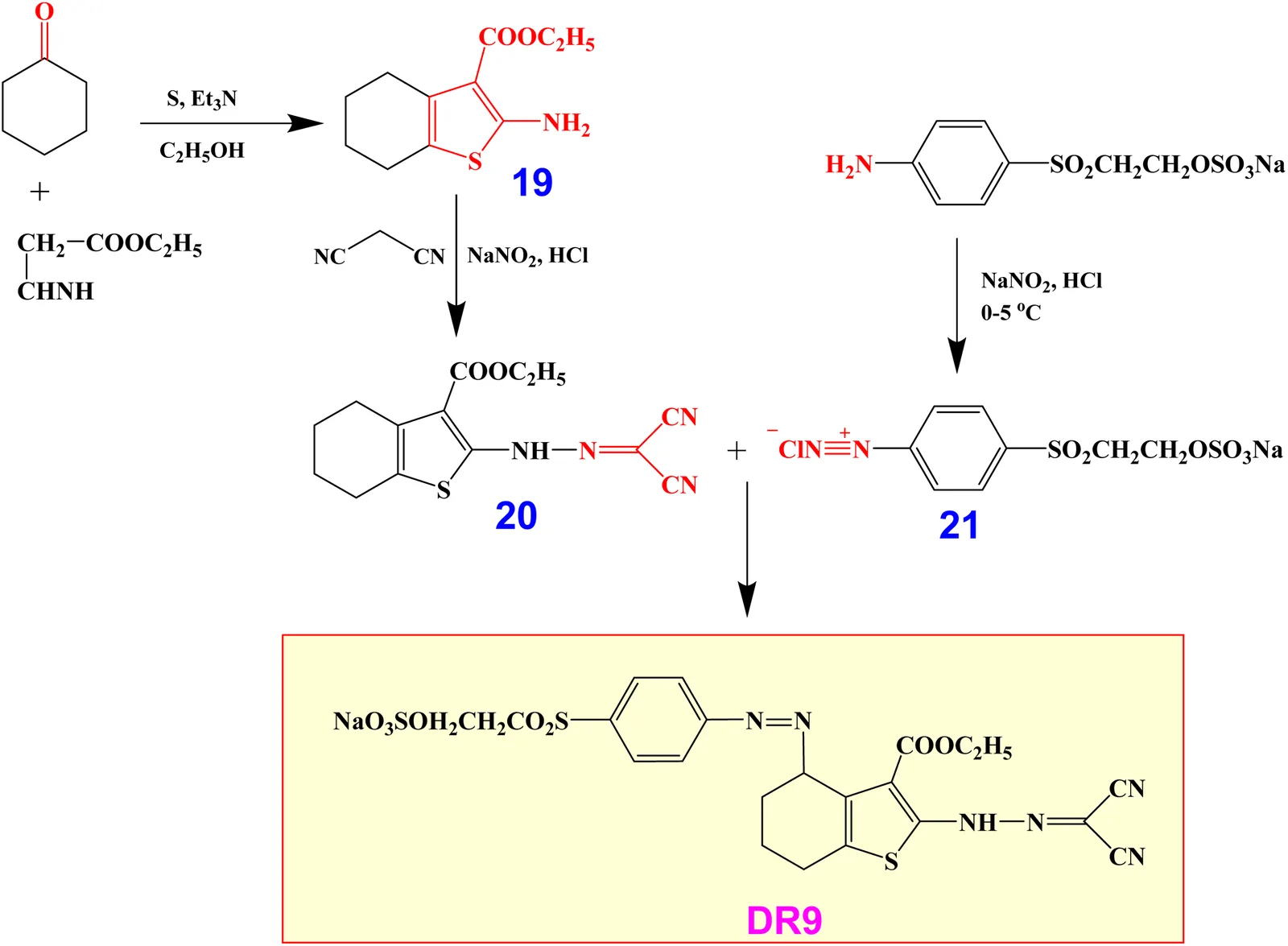
Synthesis of DR9 dye *via* hydrazine formation and vinyl sulfone–hydrazino coupling.^49^

Scheme 10 shows the two-step pathway for the synthesis of DR10 dyes with a triazine core. The process begins with two pre-synthesized key intermediates: a isatin hydrazide (22) and a dichlorotriazine intermediate (23). An amino-containing chromophore reacts selectively with the active chlorine on the triazine core under mild conditions (50 °C, in the presence of a base) to form a covalent bond. The choice of chromophore determines the identity and color of the final dye. For dye DR10a (brown powder), 3-aminobenzanthrone (24) in acetic acid with sodium acetate is used. For dye DR10b (red powder), 2-aminoanthraquinone (25) in THF with sodium carbonate as the base is employed. The resulting structure is composed of three main components: a large chromophore (benzanthrone or anthraquinone), a dichlorotriazine core (with one remaining reactive chlorine), and an isatin unit linked to the triazine *via* a hydrazone bridge. This modular strategy enables the design of a family of reactive dyes with diverse optical properties by varying the aminochromophore (DR10a: yield 87%, DR10b: yield 60%).^50^

**Scheme 10:**
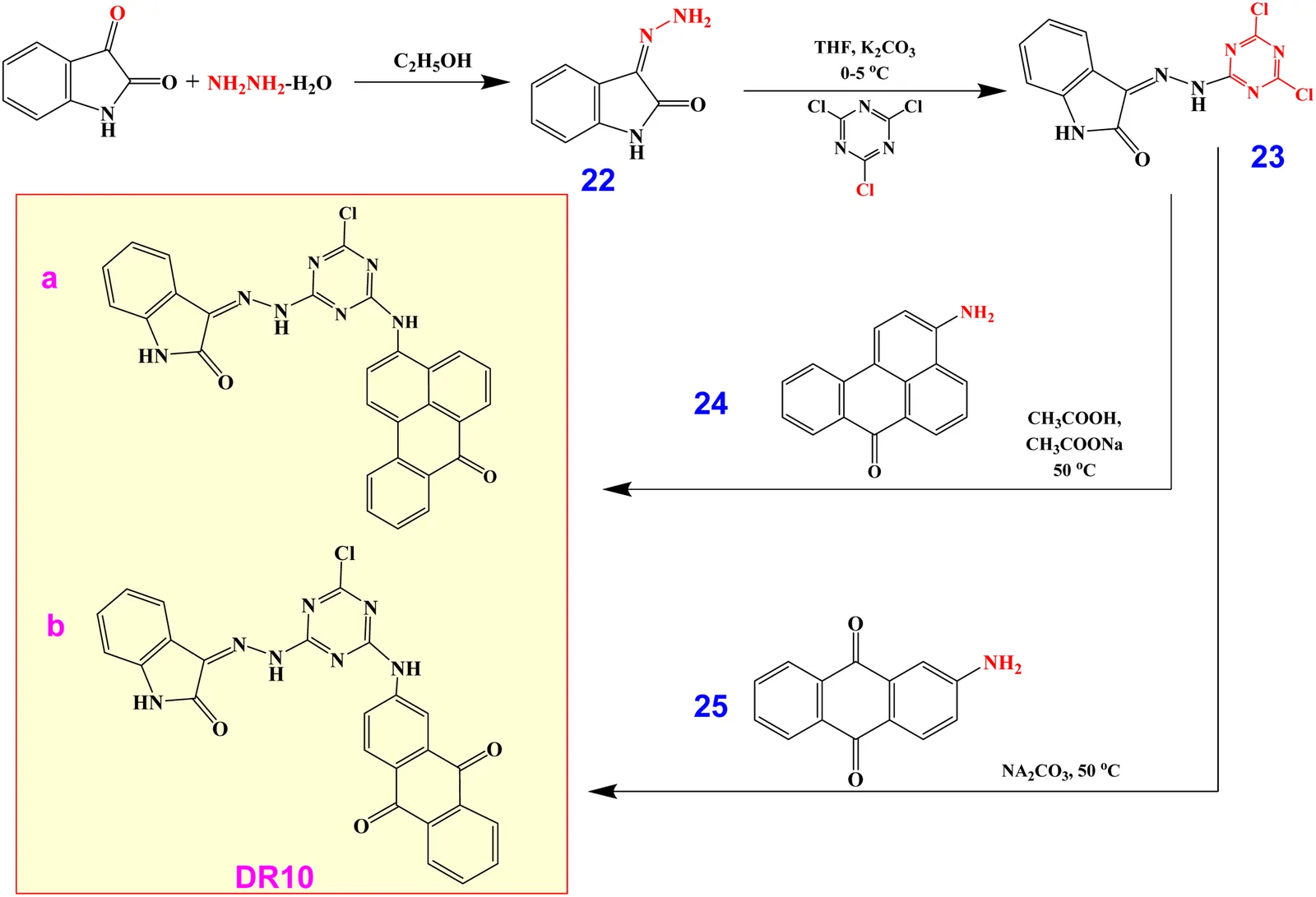
Modular synthesis of DR10 dyes *via* isatin–triazine coupling and aminochromophore variation.^50^

In a multi-step synthesis to produce DR11, three related anthraquinone dyes with different side chains are generated by linking a pyrazole unit to an anthraquinone chromophore *via* a pyrazole-blocked isocyanate (or *N*-carbamoylpyrazole) linkage (Scheme 11). The process begins with the preparation of an amino-anthraquinone chromophore (26) through the high-temperature aromatic substitution of 1-hydroxy-4-nitroanthraquinone with 1,4-phenylenediamine in 4-nitrobenzene as solvent. The reaction is carried out at 150 °C for 20 minutes and then at 210 °C for 12 hours. In parallel, various pyrazole derivatives (unsubstituted, 3,5-dimethyl, and 3,5-di-*tert*-butyl) are synthesized *via* cyclization of a diketone with hydrazine hydrate in methanol at room temperature for 1 hour, yielding white solids (*e.g.*, 3,5-di-*tert*-butylpyrazole, 86%). The key step involves converting the amino group of the aminoanthraquinone into a reactive isocyanate (27) using triphosgene in toluene at 130 °C under an N_2_ atmosphere, with triethylamine as a base. This isocyanate then reacts directly with the pyrazole nitrogen (28) at 40 °C in toluene to form a urethane bond. The resulting dyes DR11 all feature an anthraquinone chromophore connected through a phenyl-urethane bridge to a pyrazole ring and differ only in the substituents on the pyrazole (hydrogen, methyl, or *tert*-butyl), with isolated yields of 89%, 88%, and 90%, respectively.^51^

**Scheme 11:**
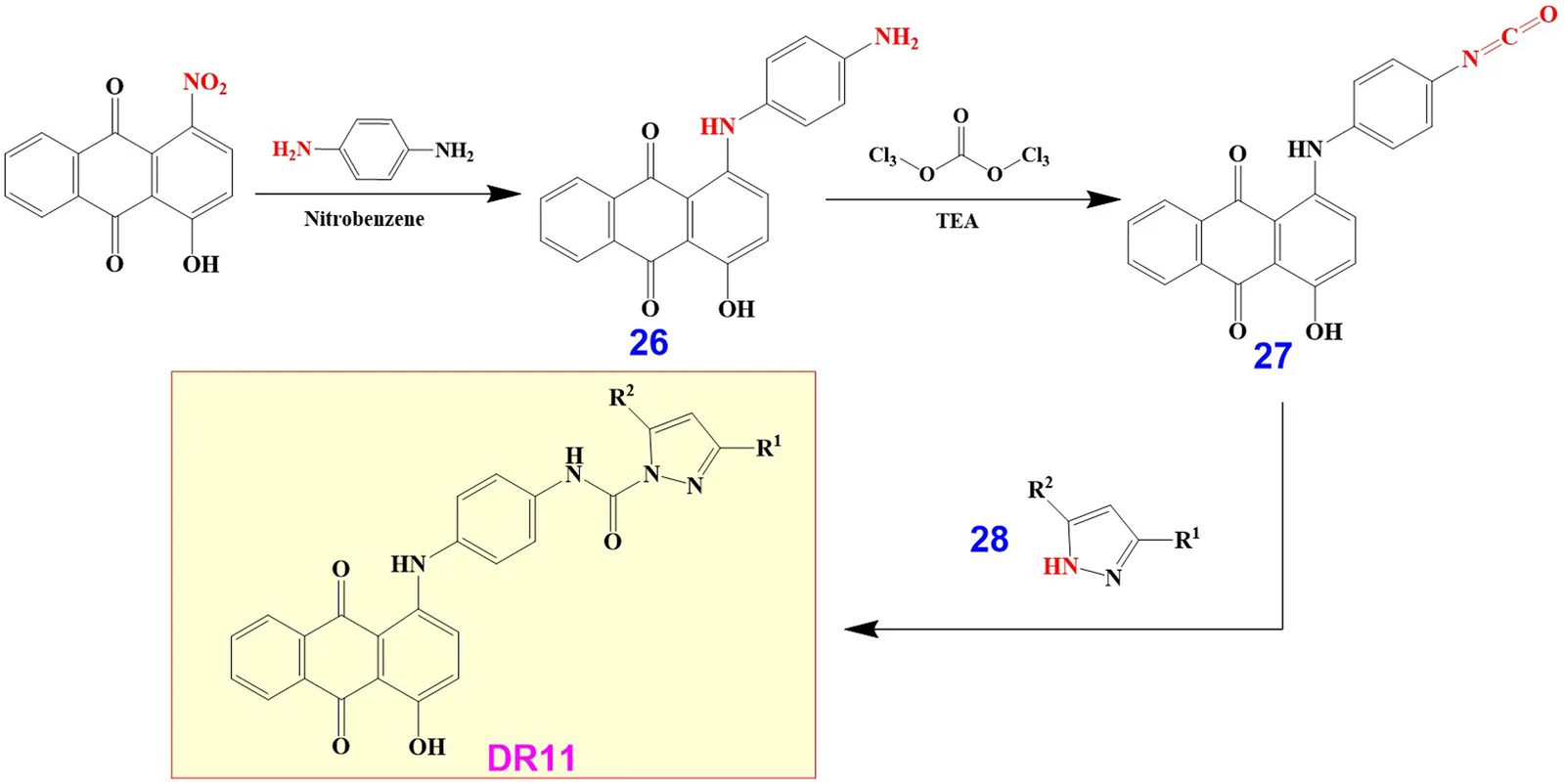
Synthesis of DR11 dyes *via* isocyanate formation and pyrazole–anthraquinone urethane coupling.^51^

The synthesis process of DR12 describes a straightforward two-step preparation of a DR dye from its precursor (30) (Scheme 12). In the first step, a classic azo dye is prepared: 4-nitroaniline hydrochloride is treated with HCl and water at 0 °C. Sodium nitrite in water is added slowly. After 30 minutes, urea is added to eliminate unreacted nitrous acid. The resulting diazonium salt solution is then coupled by dropwise addition at 0–5 °C with compound (29) in acetic acid over 1 hour. No external catalyst is used in this step. After neutralization with KOH solution, the solid is collected, stirred with 5% Na_2_CO_3_ solution, filtered, washed with water, and crystallized from ethanol, yielding dye (30) in 30% yield. This azo coupling produces dye (30), which features anitro-substituted azo structure and serves as the precursor for the next step. In the second step, dye (30) is dissolved in dichloromethane, and collidine is added as an organic base. After cooling to 0 °C, cyanuric chloride is slowly added, and the reaction mixture is stirred at 0 °C for 2 hours. No additional catalyst is required. One of the reactive chlorine atoms on the triazine reacts with the secondary amino group (–NHCH_3_) on the azo dye. The solid is then filtered, washed three times with ice water, and crystallized from dichloromethane, yielding DR12 (25% yield). The two remaining chlorine atoms on the triazine ring provide sites for covalent bonding with fibers, completing the formation of a DR dye.^52^

**Scheme 12:**
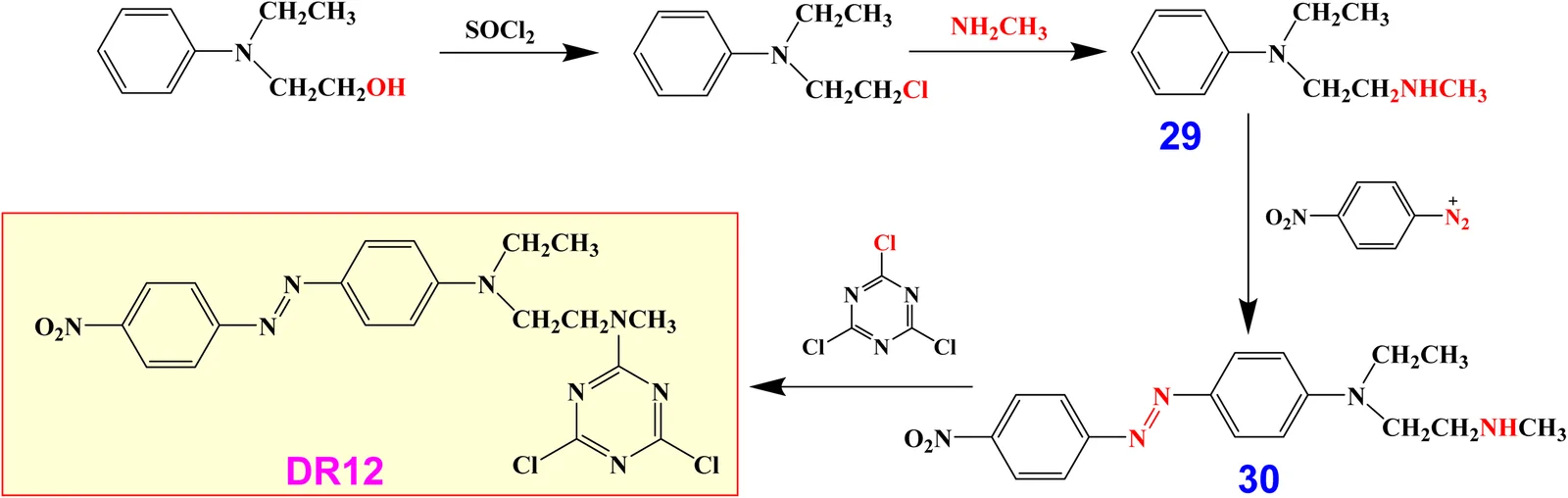
Synthesis of DR12 dye *via* azo coupling and cyanuric chloride condensation.^52^

Scheme 13 describes a classic, straightforward two-step synthesis of a series of thiazole-based DR dyes (DR13). In the first step, an aryl amine (which varies depending on the target derivative) is diazotized to form its diazonium salt, which is then coupled at 0–5 °C with an amino-thiazole derivative [2-amino-4-(*para*-tolyl)thiazole (31)] in ethanol. This azo coupling generates a thiazole-azo chromophore bearing a free amino group on the thiazole ring (32), which serves as the attachment site for the subsequent step. By varying the aryl amine, a series of related intermediates is obtained. In the second step, the amino group of the thiazole undergoes sulfonylation with an ethenesulfonyl chloride in dry DMF under cooled conditions. This forms a sulfonamide bond (–NHSO_2_–) between the amino group and the sulfonyl chloride, yielding the final DR13 dye (yield 89%). The final structure comprises a thiazole-azo chromophore, a sulfonamide bridge, and a terminal ethenesulfonyl group. Under alkaline conditions during dyeing, the terminal vinyl sulfonyl group can react with nucleophilic fiber groups under suitable conditions.^53^

**Scheme 13:**
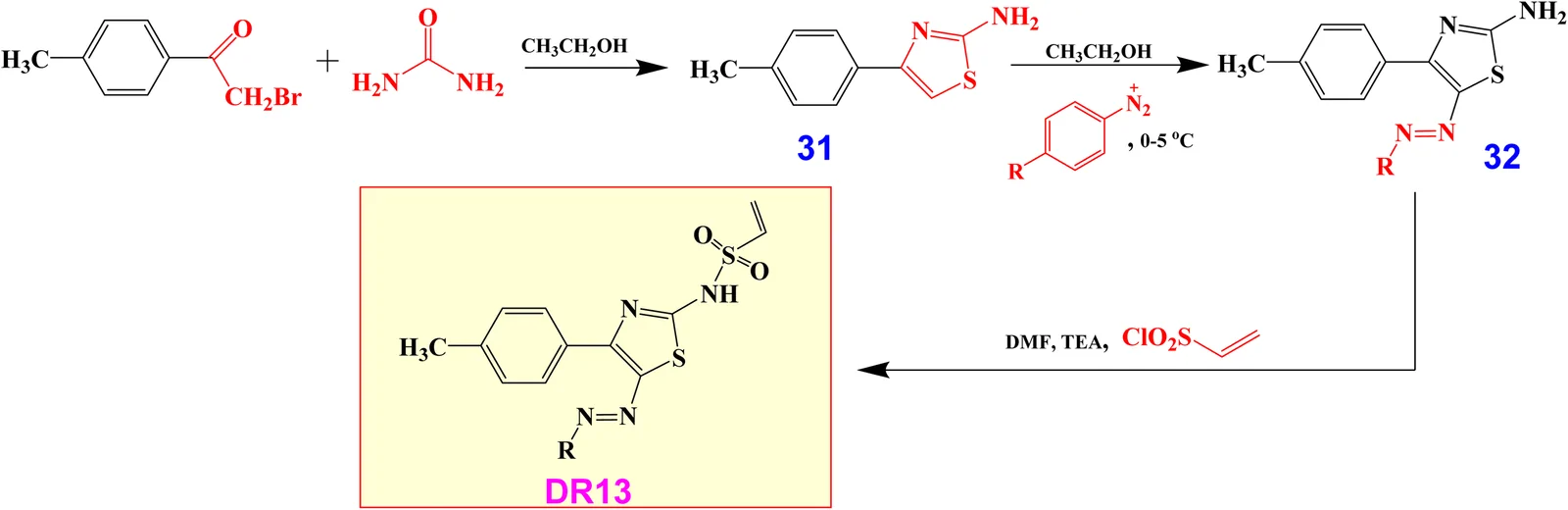
Synthesis of DR13 dyes *via* thiazole azo coupling and sulfonylation with ethenesulfonyl chloride.^53^

In a paper, a systematic three-step synthesis of a family of chlorotriazine-based DR dyes (DR14) is described (Scheme 14). The process begins with the diazotization of a classical diazo component, 2-chloro-4-nitroaniline, in an aqueous–organic medium (water and PEG 200) with hydrochloric acid at a very low temperature (0–5 °C), producing a stable yellow solution of the diazonium salt (33). The diazonium salt is immediately coupled at low temperature (pH ∼ 6) with a series of coupling components such as aniline, *m*-toluidine, and *m*-anisidine. This reaction yields three azo precursors, each bearing a free amino group (–NH_2_) on the coupling component ring, which serves as an attachment point for the final step (34). The azo precursor is then condensed with cyanuric chloride at 0–5 °C in a two-phase system (1,4-dioxane/water) in the presence of NaOH. One highly reactive chlorine atom on cyanuric chloride reacts with the amino group on the precursor, forming an aminotriazine linkage and producing the final DR14 dichlorotriazine-based DR dyes. Variation of the coupling component (aniline, toluidine, or anisidine) in step 2 introduces different substituents (H, methyl, methoxy) at *meta* to the amino group and *ortho* to the azo linkage, influencing solubility, color, and fiber interactions. The two remaining chlorine atoms on the triazine ring serve as reactive sites for covalent bonding with fibers (yield over 90%).^18^

**Scheme 14:**
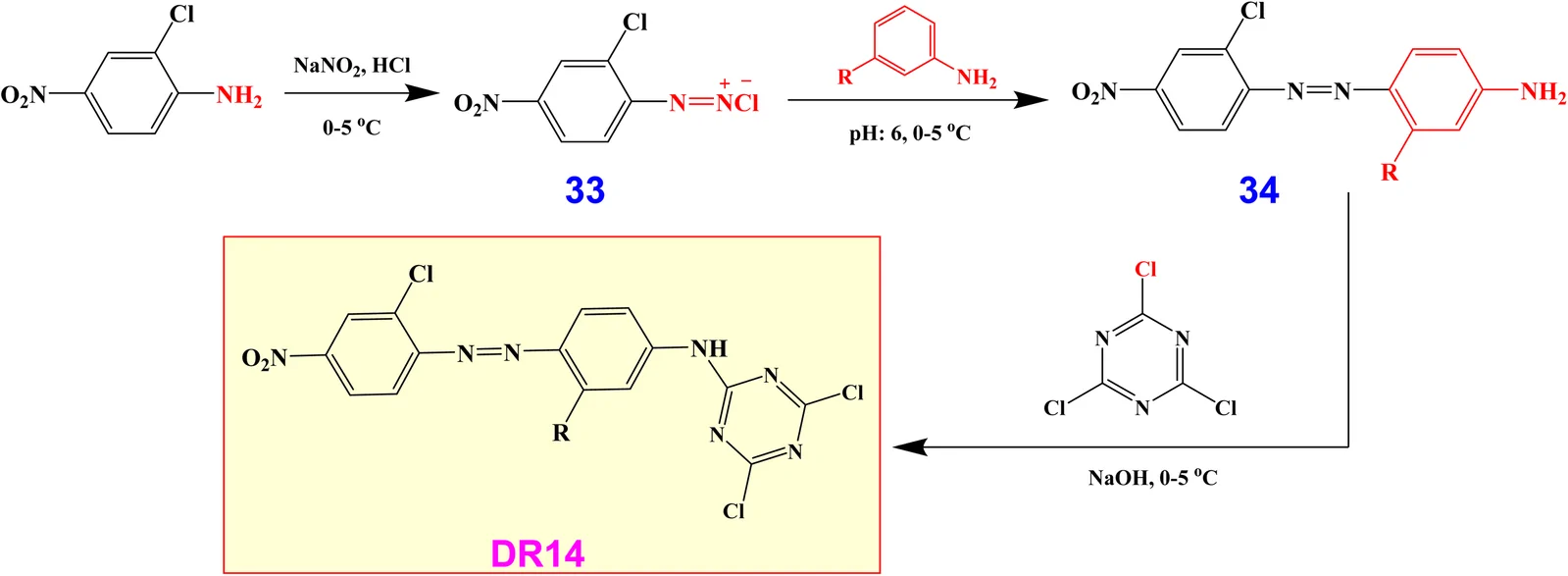
Three-step synthesis of DR14 dyes *via* diazotization, azo coupling, and dichlorotriazine condensation.^18^

Scheme 15 describes a targeted four-step synthesis of dye DR15, involving functional group modifications to tune the dye properties and create attachment sites. 2-Amino-6-nitrobenzothiazole is diazotized in a strongly acidic medium (sulfuric and phosphoric acid) to form the diazonium salt (35), which is prepared for subsequent steps. The resulting diazonium salt is reacted at low temperature with 3-methyl-1-phenyl-2-pyrazolin-5-one (pyrazolone), yielding a hybrid chromophore with an azo bond (36) that still contains a nitro group on the benzothiazole ring. The nitro group is reduced to an amino group using sodium sulfide nonahydrate and sodium bicarbonate in ethanol (37). This transformation both alters the chromophore's color properties and provides the primary attachment site for the final step. The amino group on (37) is condensed with cyanuric chloride at low temperature under neutral conditions, attaching a dichlorotriazinyl moiety to the hybrid chromophore. The final product is a complex DR15 (yield 75%) dye, ready to form covalent bonds with fibers.^31^

**Scheme 15:**
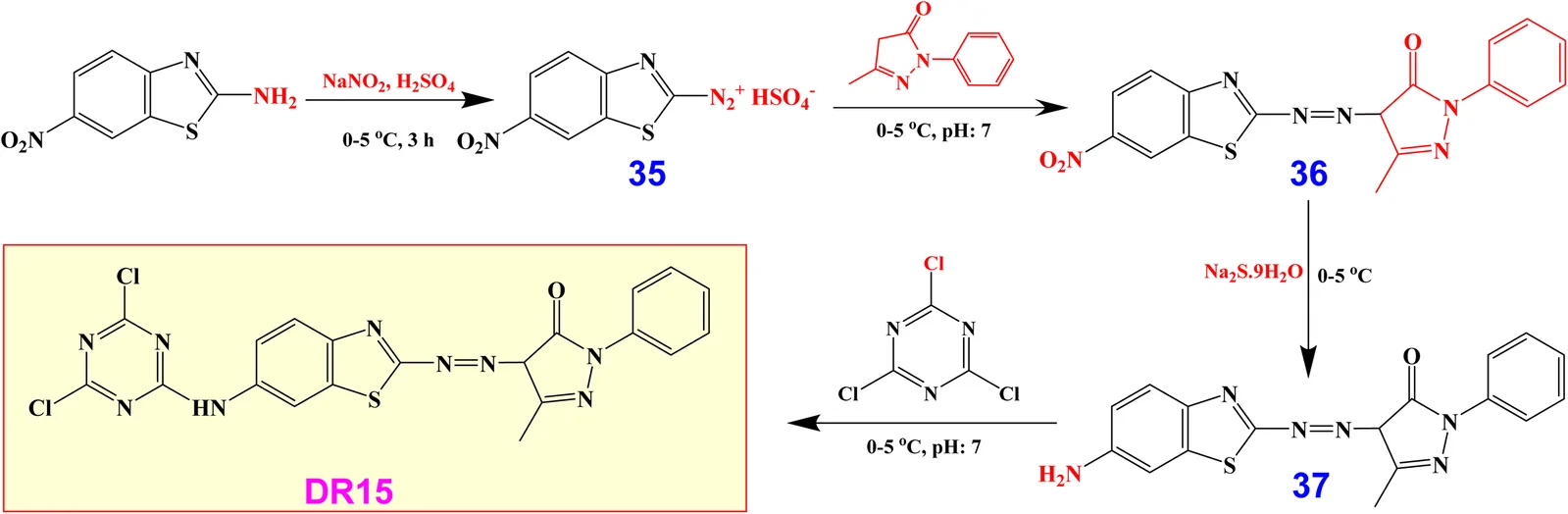
Four-step synthesis of DR15 dye *via* diazotization, azo coupling, nitro reduction, and triazine functionalization.^31^

Scheme 16 describes a direct, one-step synthesis of a vinyl sulfonyl DR dye (DR16). Unlike many previous routes that employ cyanuric chloride, the reactive group here is already present within the diazo component. An aminophenyl ethyl sulfonate sulfate (aminophenyl-4-(β-sulfatoethylsulfone)), (38) contains: (1) an amino group for diazotization, and (2) an beta-sulfatoethylsulfone group, which serves as the precursor for the reactive vinyl sulfonyl moiety. Under alkaline dyeing conditions, the sulfate group (–OSO_3_Na) is eliminated, generating a highly reactive vinyl sulfonyl group (–SO_2_CH=CH_2_) capable of covalent bonding with fibers. A tertiary aromatic amine (*N*,*N*-diethylaniline or *N*-benzyl-*N*-ethylaniline) is employed as the coupling component. The diazo component is first diazotized, and the resulting diazonium salt (39) is then coupled directly with the tertiary aromatic amine at low temperature (approximately 5 °C) in an azo coupling reaction. The final product is dye DR16 (yield 83%). In this approach, the reactive vinyl sulfonyl group is pre-incorporated into the diazo component and becomes attached to the dye immediately upon coupling, without requiring any additional steps.^32^

**Scheme 16:**
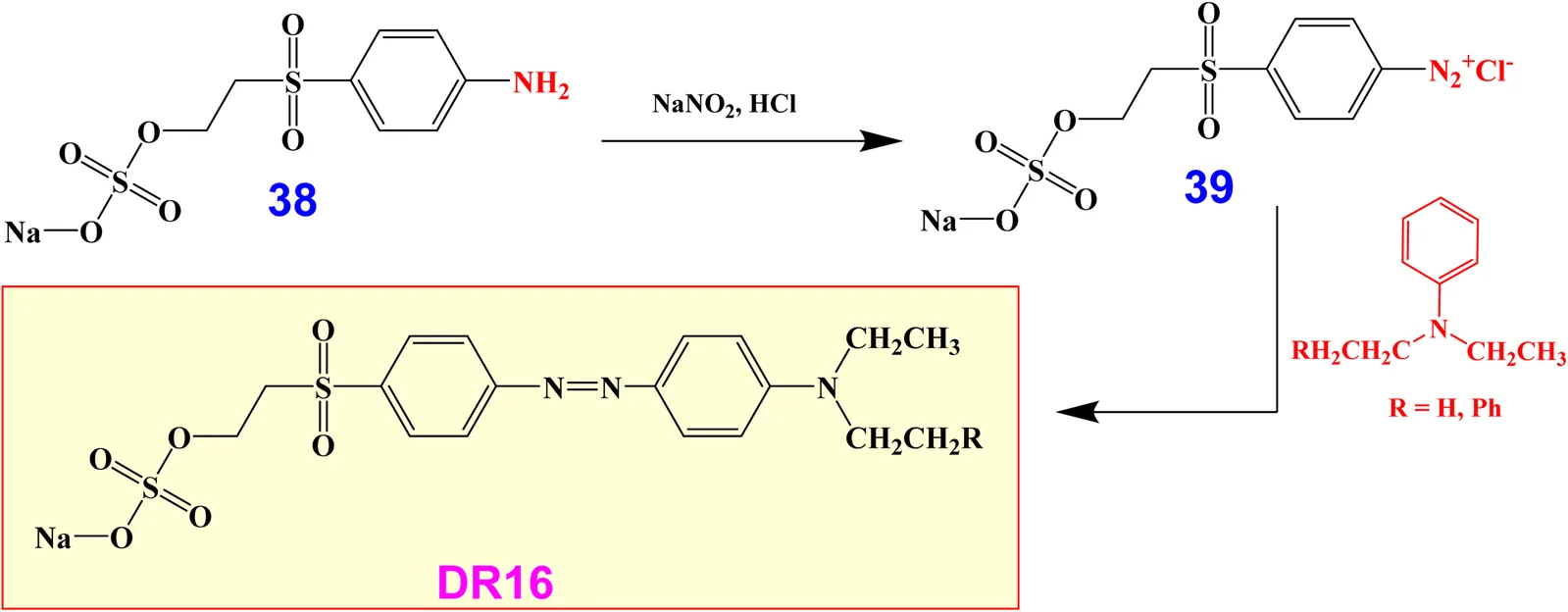
One-step synthesis of DR16 dye *via* pre-functionalized vinyl sulfonyl diazo component and azo coupling.^32^

For the synthesis of dye DR17, an innovative synthetic strategy is presented for producing two types of DR dyes, wherein an acyl fluoride (–COF) is employed as the reactive moiety instead of conventional reactive groups (such as chlorotriazine or vinyl sulfonyl) (Scheme 17).

**Scheme 17:**
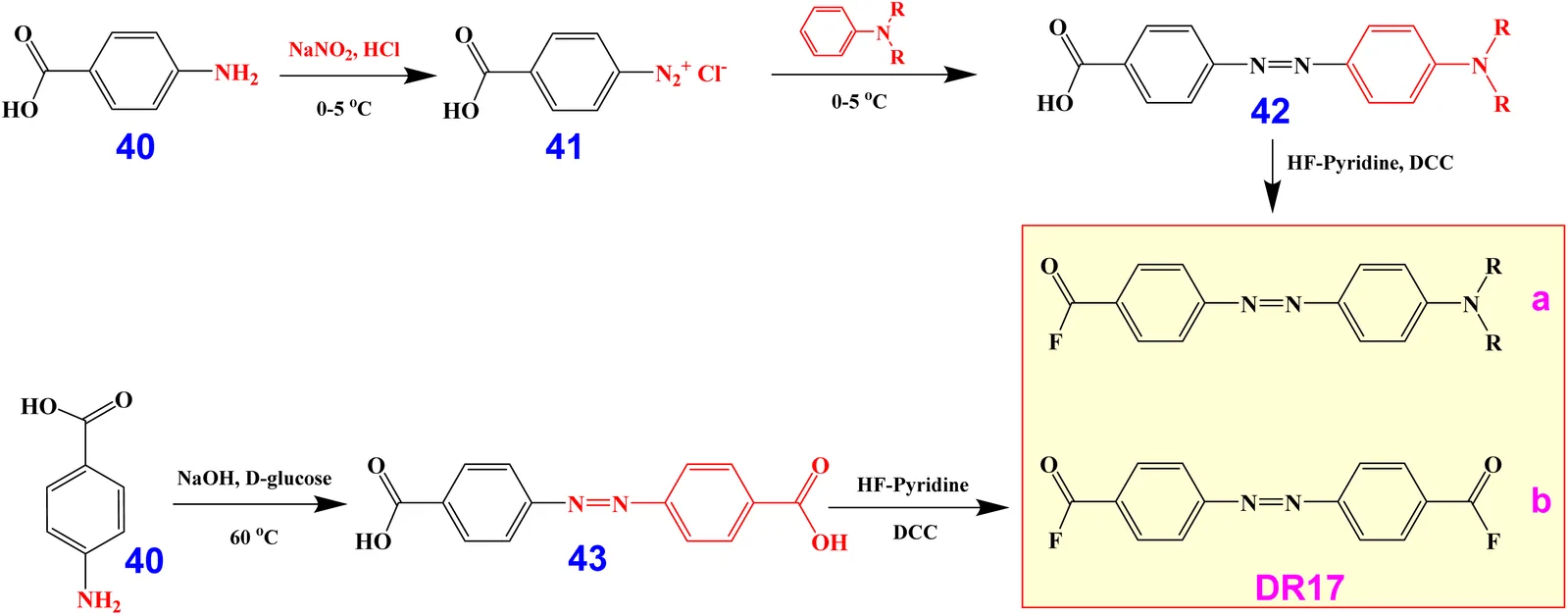
Synthesis of DR17 dyes *via* acyl fluoride formation from carboxylic acid precursors using HF–pyridine and DCC.^54^

#### For DR17a

4-Aminobenzoic acid (40) is diazotized (41) and then coupled with a azo carboxylic-acid product (42). This yields an azo compound containing a carboxylic acid group (–COOH) on one side. The carboxylic acid is converted to a reactive acyl fluoride using a mixture of HF–pyridine as the fluoride source and DCC (dicyclohexylcarbodiimide) as the dehydrating agent. This produces a monofunctional reactive dye, DR17a, bearing one acyl fluoride group.^54^

#### For DR17b

Instead of conventional diazotization, a reductive coupling method is employed. Compound (40) is reduced with d-glucose under alkaline conditions, converting nitro groups to amino groups, which are then coupled to form a symmetric dicarboxylic acid azo compound (43). Both carboxylic acid groups are converted to acyl fluorides under the same HF–pyridine/DCC conditions, yielding DR17b, a bifunctional reactive dye with two acyl fluoride groups at opposite ends of the molecule.^54^

This method describes the direct, one-step synthesis of two series of vinyl β-sulfatoethylsulfone-based DR dyes (DR18) (Scheme 18). The diazo component, 2-((4-aminophenyl)sulfonyl)ethyl hydrogen sulfate, serves both as a diazotizable amine precursor and as a pre-installed vinyl sulfone reactive group. Diazotization is carried out in an acidic medium at low temperature using sodium nitrite, generating the diazonium salt (44), which contains the reactive vinyl sulfone functionality. Two distinct coupling pathways are then employed to produce different dye series:

**Scheme 18:**
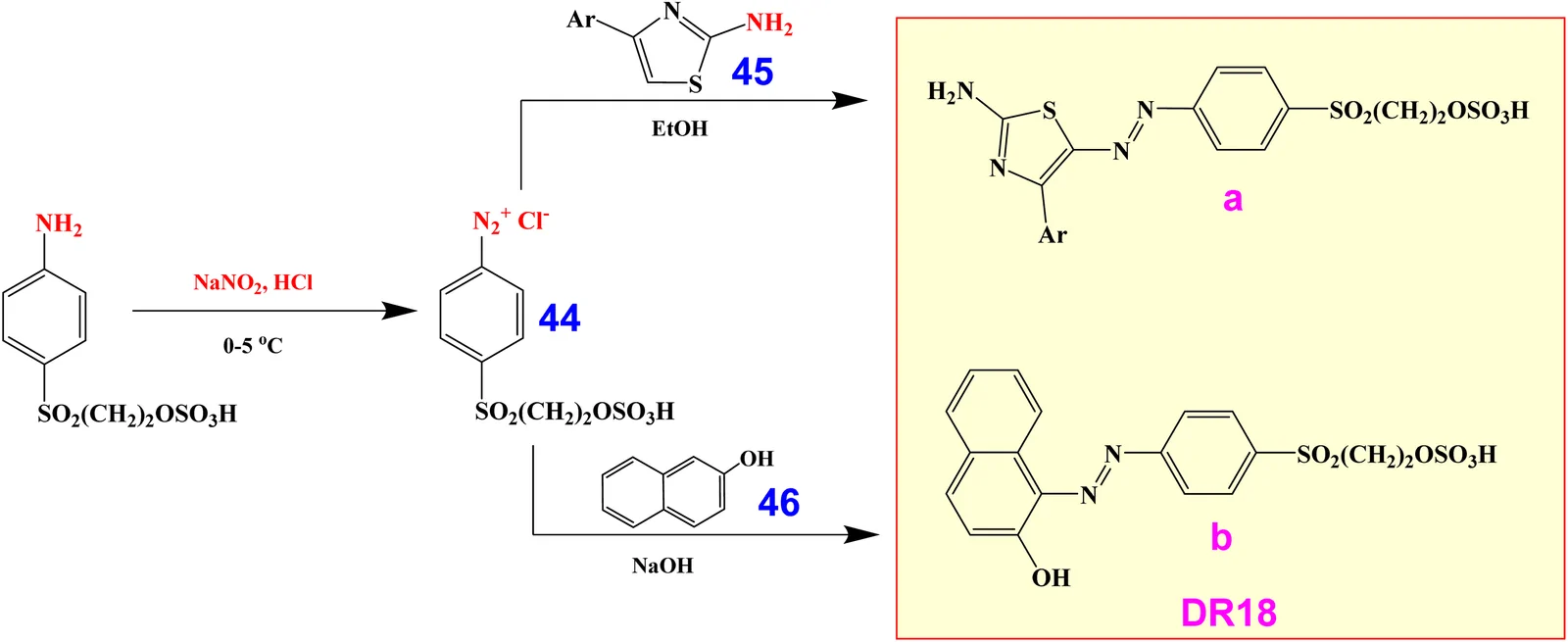
One-step synthesis of DR18 dyes *via* vinyl sulfone-functionalized diazo component and coupling with aminothiazole or α-naphthol.^39^

#### Synthesis of aminothiazole-based dye (DR18a)

The diazonium salt (44) undergoes azo coupling in ethanol with a series of 2-aminothiazole derivatives (45) in the presence of sodium acetate to maintain a near-neutral pH, under ice-bath conditions. The resulting DR18a dyes contain, in addition to the azo bond and the reactive vinyl sulfone group, a thiazole ring bearing an amino group. This amino group on the thiazole provides a chemically active site for further modification or fiber interaction (yield 75%).^39^

#### Synthesis of hydroxy-naphthol-based dye (DR18b)

The diazonium salt (44) is coupled with a classical coupling component, α-naphthol (46), dissolved in an alkaline medium. This reaction produces the DR18b dyes, which contain a naphthol moiety with a hydroxyl group capable of participating in subsequent interactions or fiber bonding.^39^

Scheme 19 illustrates the rational, multi-step synthetic route for hybrid reactive dyes featuring a triazine–thiazole–pyrazolone framework, incorporating a thiazole derivative bearing a pyridine moiety. A primary aminothiazole is nitrosated at low temperature in an acidic medium (HCl/ethanol) (47) using isoamyl nitrite, introducing a highly reactive nitroso group (–N=O) at the 5-position. This functionality serves as a key reactive site for subsequent condensation reactions. The aminothiazole (47) first reacts with cyanuric chloride and is subsequently nitrated at the 4-position. These divergent pathways demonstrate the synthetic flexibility of this approach. The nitroso group on intermediate (48) condenses with 1,3-substituted-5-pyrazolone derivatives, forming an imine bond (C=N) and generating complex hybrid dyes. The final DR19 products comprise: a reactive dichlorotriazine core for fiber attachment; a thiazole ring linked to a pyridine moiety; a pyrazolone ring functionalized with various aryl groups (yield 98%). These dyes are expected to exhibit deep coloration (blue, violet, or green) with high stability and strong fiber fixation.^55^

**Scheme 19:**
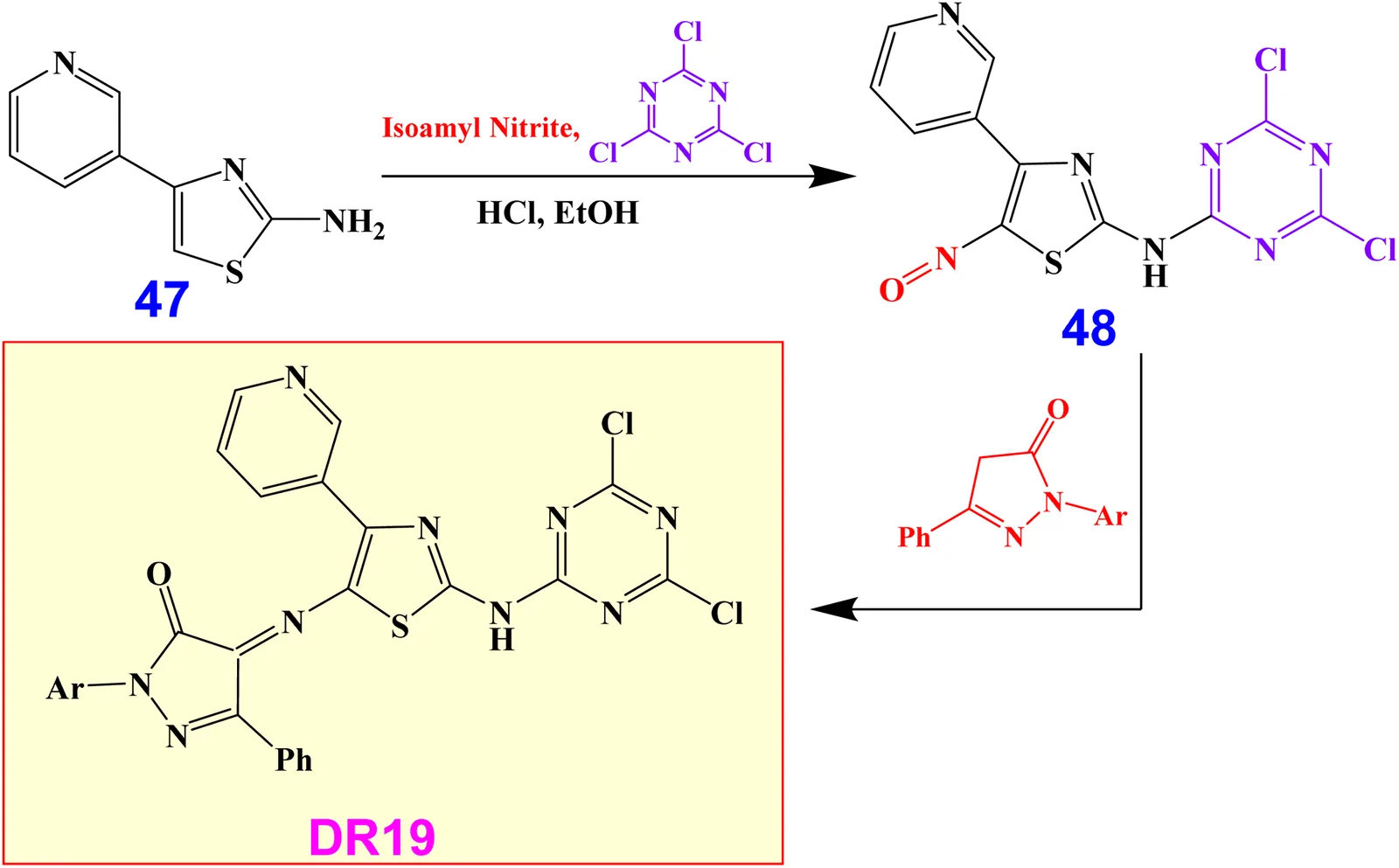
Multi-step synthesis of DR19 dyes *via* nitrosation, triazine coupling, and pyrazolone condensation.^55^

A four-step synthesis of dye DR20 featuring a novel pyrrolocyanine chromophore is presented (Scheme 20). Malononitrile undergoes base-catalyzed dimerization to form a tricyano dimer, which is then cyclized with diethyl oxalate to generate a highly conjugated pyrrolotricyanine system (51) exhibiting strong absorption in the visible-near-infrared region. An aminoaryl alcohol (49) reacts at room temperature with cyanuric chloride *via* its hydroxyl group, forming a triazine ether (50) containing a reactive dichlorotriazinyl core linked to an ethyl–aminoaryl chain. The chromophore (51) and the carrier (50) are coupled in DMF using methanesulfonyl chloride as an activator. An active group on the chromophore reacts with the secondary amine group on the carrier, forming a covalent bond. The resulting dye DR20 comprises a pyrrolocyanine chromophore, an aminoaryl linker, and a terminal reactive dichlorotriazine group for fiber attachment (yield 51%).^26^

**Scheme 20:**
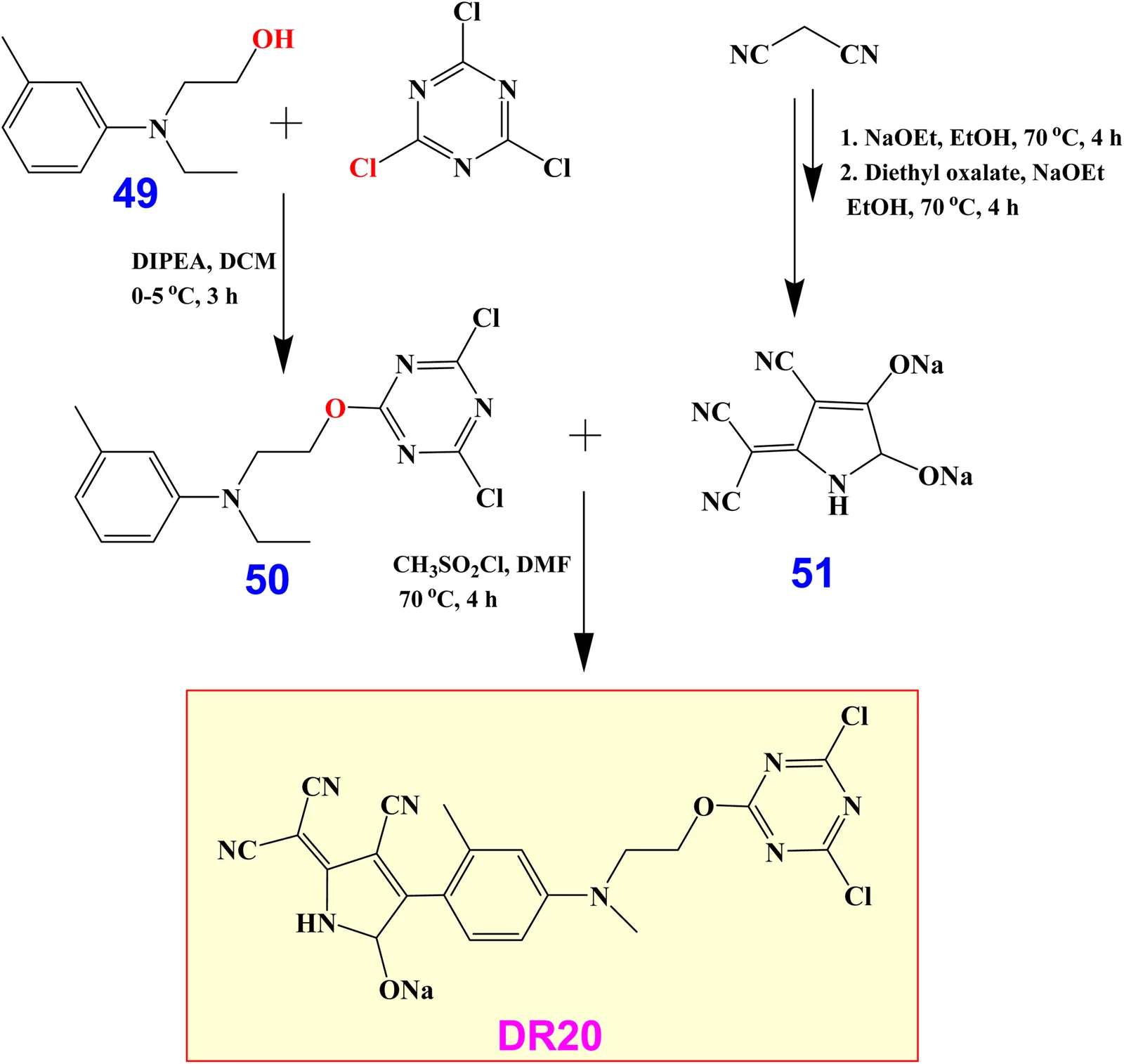
Four-step synthesis of DR20 dye *via* pyrrolocyanine chromophore formation and dichlorotriazine coupling.^26^

*Para*-phenylenediamine is reacted with cyanuric chloride in the presence of a base (Scheme 21). Under controlled molar ratios and ambient temperature, one chlorine atom of cyanuric chloride undergoes nucleophilic substitution with an amino group of phenylenediamine. The resulting monochlorotriazine derivative (52) is linked to a phenylamine moiety on one side, while retaining one reactive chlorine atom and one free amino group on the opposite side for further functionalization. Intermediate (52) is then reacted with a diaminoanthraquinone chromophore under reflux conditions. The free amino group of (52) forms a covalent bond with a reactive site on the anthraquinone ring—likely *via* nucleophilic substitution of a halogen or at an activated position. The final dye (DR21) is blue and consists of an anthraquinone chromophore linked through a phenyl–diaminotriazine bridge to a terminal reactive chlorotriazine group (yield 65%).^19^

**Scheme 21:**
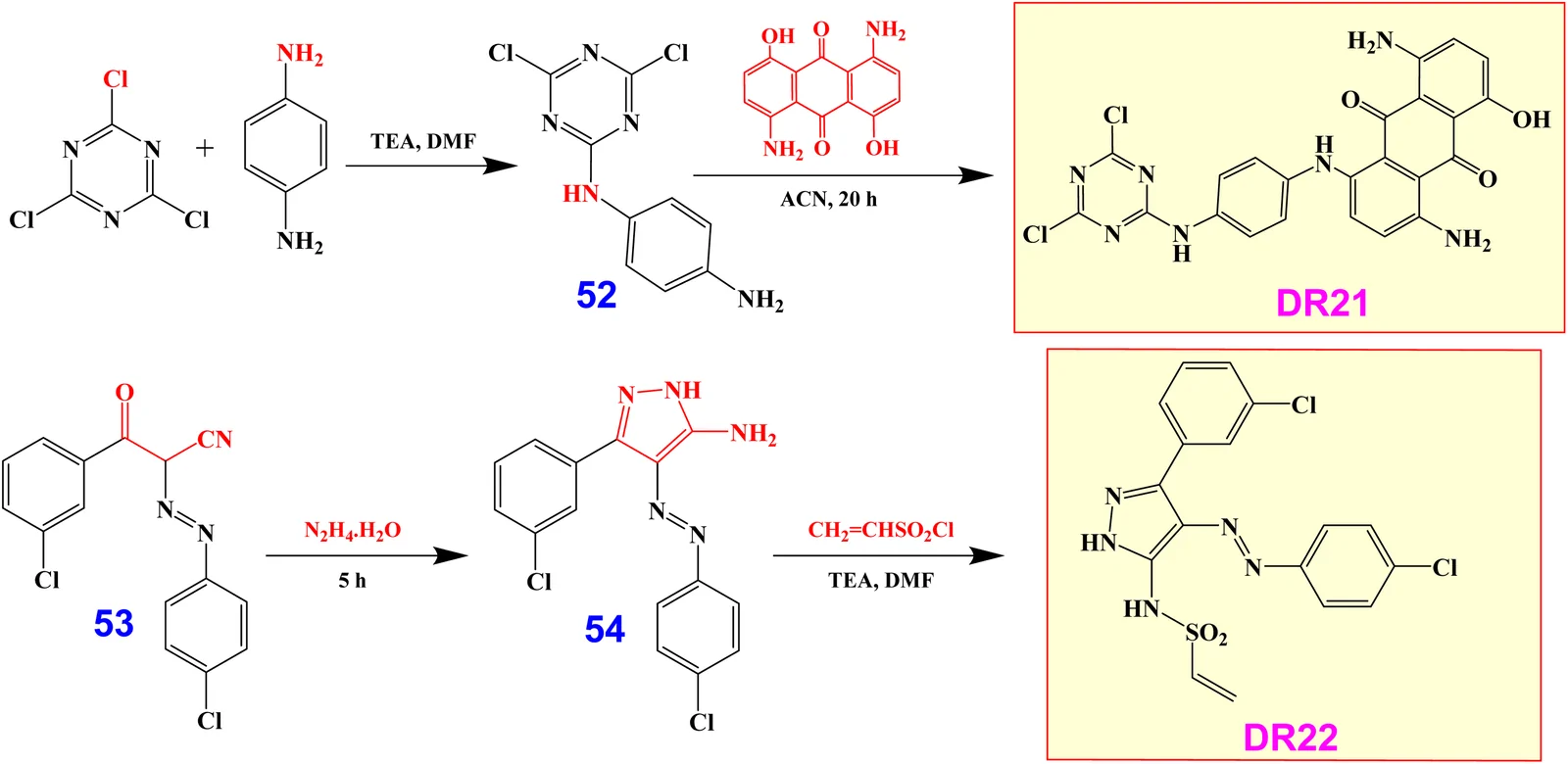
Synthesis of DR21*via* phenylenediamine–triazine coupling and anthraquinone functionalization, and DR22*via* pyrazolone azo coupling and vinyl sulfonylation.^19^

This independent route focuses on constructing an azo dye containing a pyrazolone system, followed by introduction of a vinyl sulfone reactive group. An azo–ketonitrile derivative (53) is heated with hydrazine hydrate to induce cyclization, forming a 5-aminopyrazolone core (54) and generating a new heterocyclic system. The amino group on the pyrazolone ring (54) undergoes sulfonylation with vinyl sulfonyl chloride at low temperature in the presence of a base (triethylamine), yielding the sulfonamide-linked vinyl sulfone dye (DR22; yield 87%). Under alkaline dyeing conditions, the vinyl sulfone group is capable of forming covalent bonds with fibers.^19^

Scheme 22 presents an innovative three-step synthesis for DR dyes incorporating a novel epichlorohydrin-based reactive group. Departing from conventional triazine or vinyl sulfone moieties, a cyclic epoxide is introduced as the reactive site. An alcohol precursor (55) is activated using *N*-chlorosuccinimide (NCS) with acetic anhydride and DMAP, generating an acetate ester intermediate (56). This intermediate is then reacted with epichlorohydrin under reflux, producing a chlorohydrin intermediate (57) bearing a 3-chloro-2-hydroxypropyl moiety. Intermediate (57) is then coupled with diazonium salts derived from three different diazo components (2-chloro-4-nitroaniline, 4-nitroaniline, and 2-bromo-4,6-dinitroaniline) under acidic, low-temperature conditions, yielding the DR23 dyes (yield 73%).^56^ The terminal chlorohydrin group serves as a precursor to the reactive epoxide, which is generated *in situ* under alkaline dyeing conditions and subsequently reacts with cellulose hydroxyl groups.

**Scheme 22:**
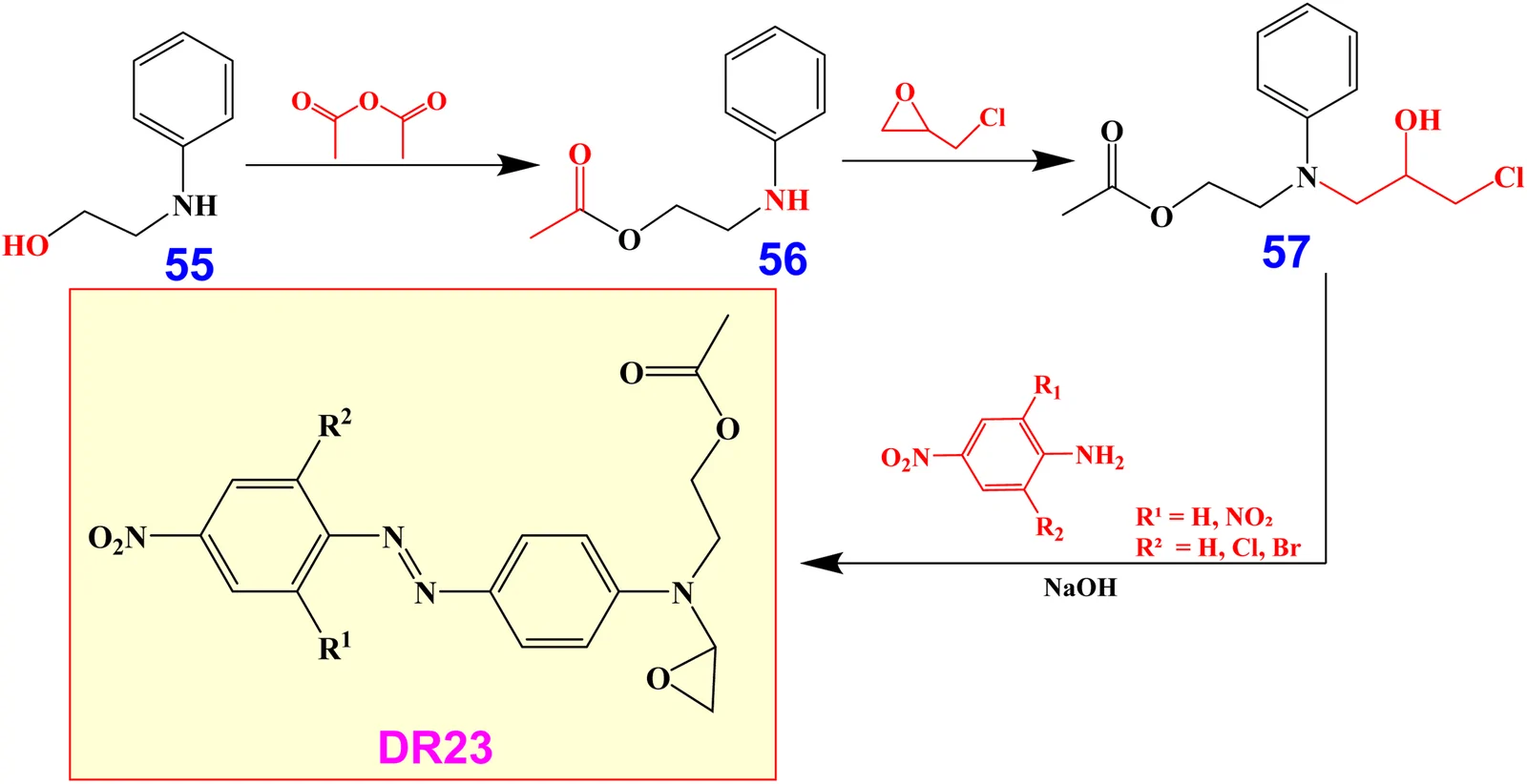
Three-step synthesis of DR23 dyes *via* epichlorohydrin-derived epoxide functionalization and azo coupling.^56^

The study presents a modular strategy for the synthesis of thiazole-based compounds as precursors to DR dyes, all centered around a 2-aminothiazole core (Scheme 23). 2-Amino-4-aryl thiazoles bearing a free amino group at the 2-position are acylated with chloroacetyl chloride to generate 2-chloroacetamido derivatives (58). The chloroacetamide moiety introduces a reactive site for further substitution and can serve as a precursor for fiber attachment. The chloroacetamido intermediates (58) undergo direct azo coupling with aromatic diazonium salts to yield 5-arylazo-thiazoles (DR24, yield 93%), in which the reactive group is already pre-installed.^57^

**Scheme 23:**
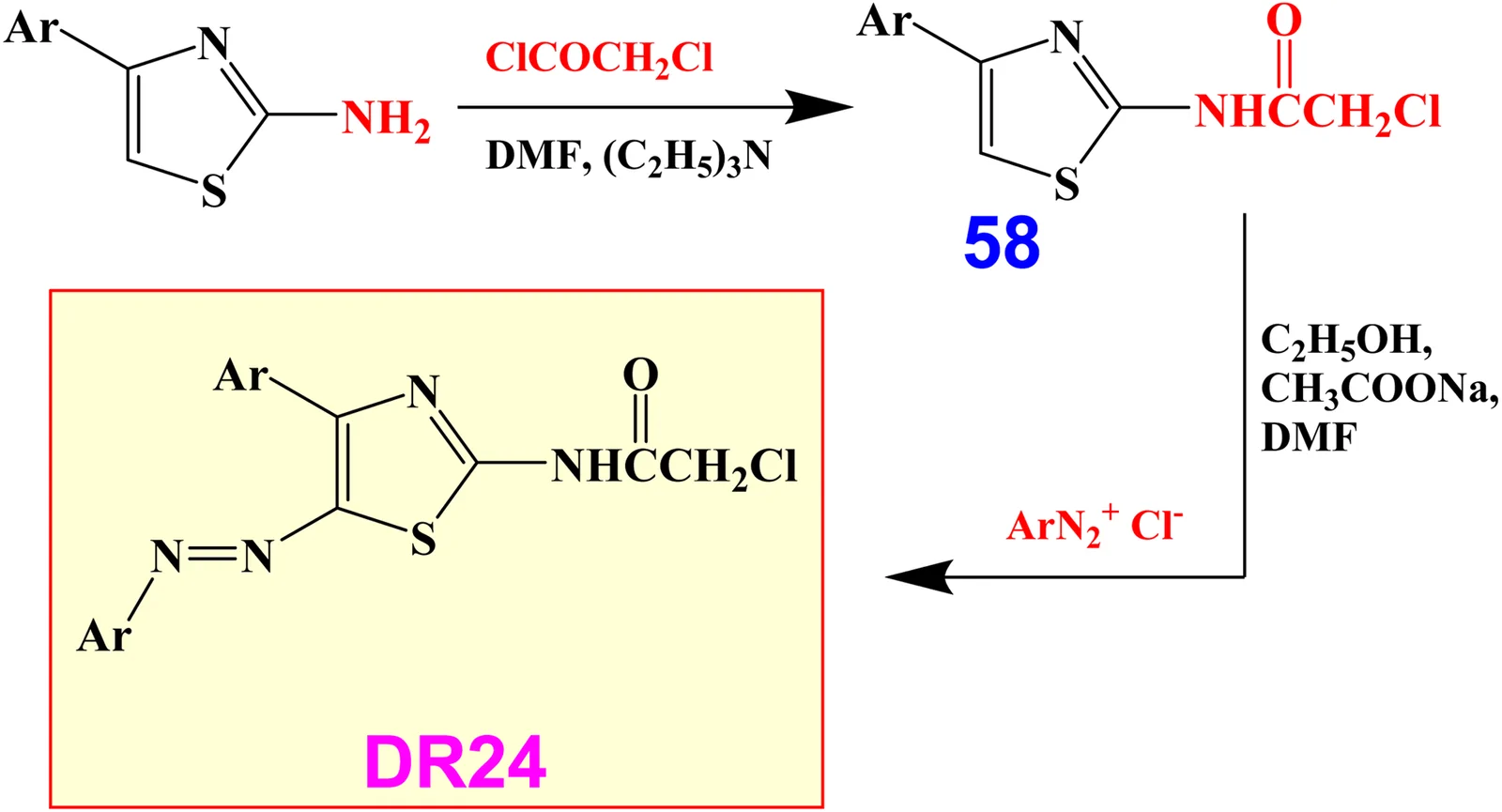
Modular synthesis of DR24 dyes *via* chloroacetamidation of 2-aminothiazole and direct azo coupling.^57^

Scheme 24 describes a direct, one-step synthesis of vinyl sulfone-based DR dyes employing a diazo component pre-functionalized with a reactive group, coupled with pre-activated heterocyclic pyrrolinone chromophores. The diazo component, 1-aminobenzene-4-β-sulfatoethylsulfone, serves as the vinyl sulfone precursor, while the chromophores are pyrrolinone esters substituted at the 2-position (with phenyl, thienyl, or 4-bromophenyl) and at the 3-position (with ethyl ester), providing a conjugated system for intense yellow to orange-red coloration. In aqueous HCl at 0 °C, the reactive diazonium salt (59) is generated, which is then coupled with the pyrrolinone ring in cold ethanol to form the azo bond. The final DR25 dyes feature a hybrid pyrrolinone–azo chromophore and a pre-installed vinyl sulfone reactive group (yield over 70%).^58^

**Scheme 24:**
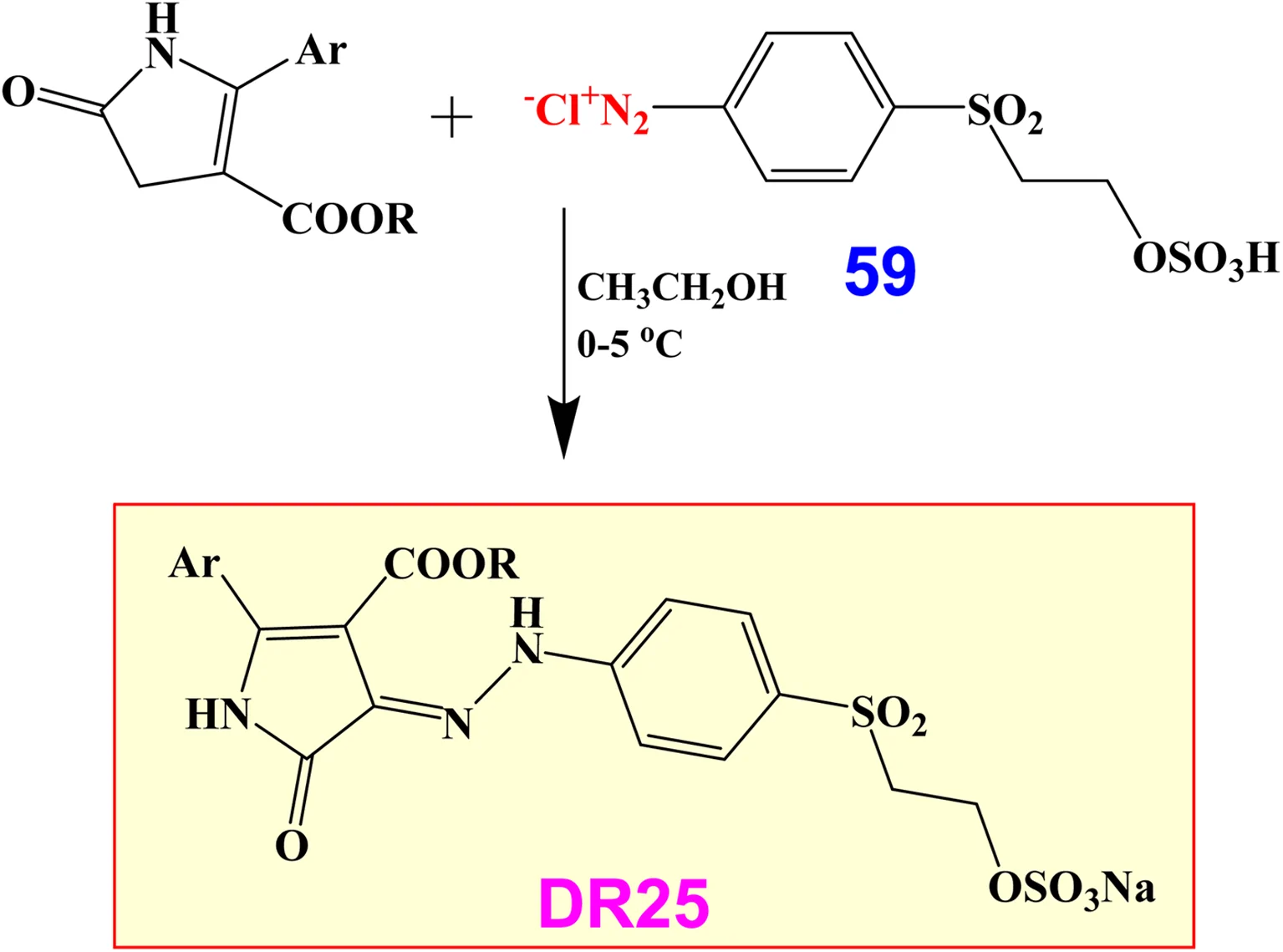
One-step synthesis of DR25 dyes *via* vinyl sulfone diazo component and pyrrolinone coupling.^58^

A straightforward, one-step method for converting conventional azo dyes into DR dyes is described, achieved through the introduction of a reactive chlorotriazinyl group (Scheme 25). Two precursor azo dyes are employed: dye (60), obtained by coupling 4-nitroaniline diazonium salt with 2-naphthol, and dye (61), formed by coupling the same diazonium salt with Naphthol AS (an amide derivative of 2-hydroxy-3-naphthoic acid). Both dyes contain a free hydroxyl group on the naphthyl ring. In this step, the hydroxyl group undergoes nucleophilic aromatic substitution with cyanuric chloride in dichloromethane at 0–5 °C. Completion of the reaction is indicated by stabilization of the pH, signifying that no further acid is being generated. The resulting DR dyes, DR26 (yield 84%) and DR27 (yield 85%), comprise a nitro-substituted azo chromophore, an ether linkage connecting the naphthyl ring to a dichlorotriazinyl core, and two remaining chlorine atoms on the triazine ring, which serve as reactive sites for covalent bonding with fibers^59^

**Scheme 25:**
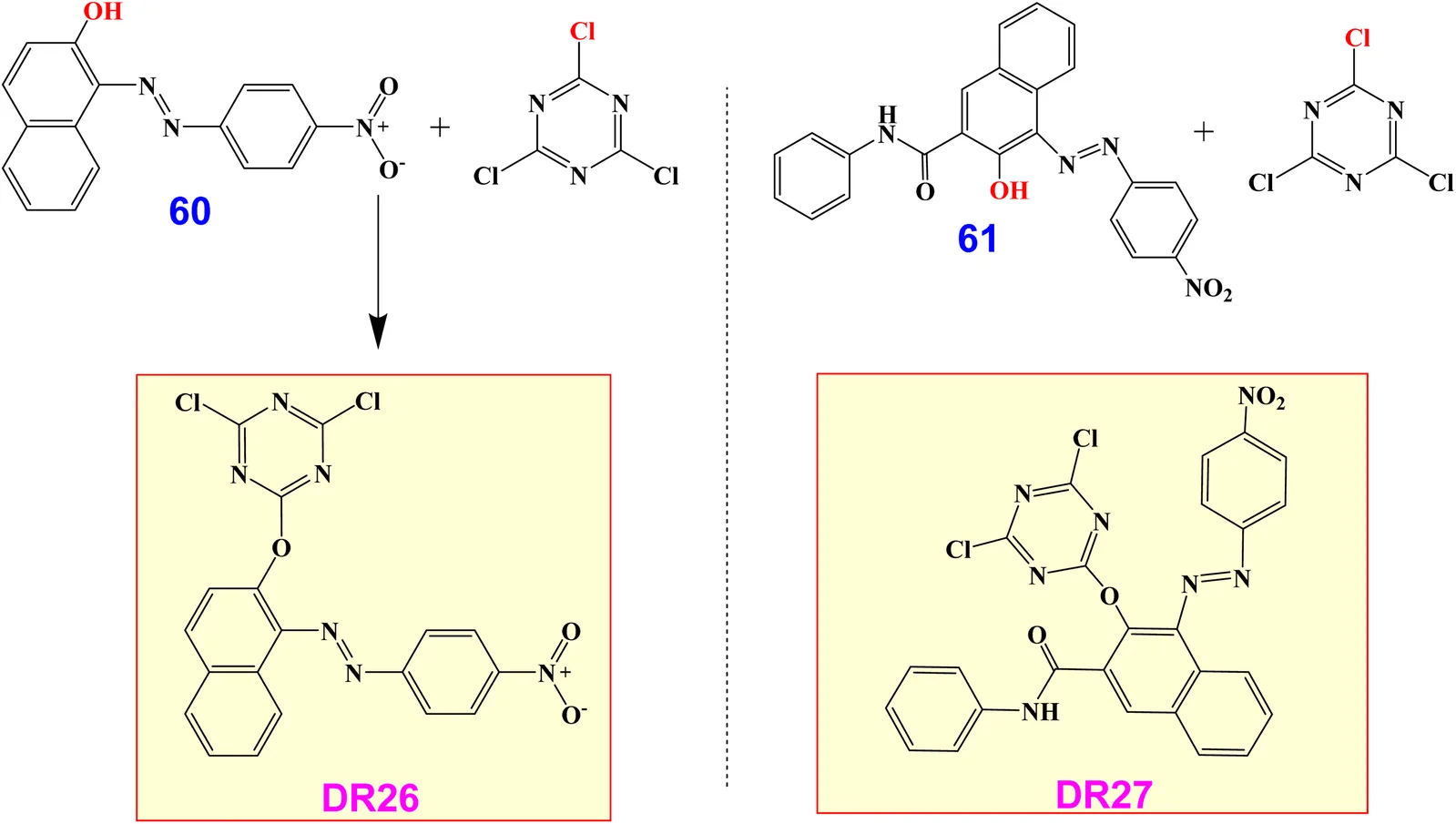
One-step conversion of conventional azo dyes to DR26 and DR27*via* chlorotriazinyl ether formation.^59^

The synthesis of dye DR28 follows a sequential three-step strategy, beginning with the construction of a nitro-substituted azo chromophore, followed by reduction of the nitro group to an amine, and finally attachment of the reactive triazine moiety (Scheme 26). 4-Nitroaniline was diazotized, and the resulting diazonium salt was coupled with *N*,*N*-diethylaniline under slightly acidic conditions to yield the monoazo dye (62) as a red powder. This product comprises the azo chromophore and a nitro group located on the diazo component side. Using Na_2_S·9H_2_O, (62) was subjected to selective reduction. The nitro group was converted to a primary amine, affording the amino-functionalized azo dye (63) as a deep orange powder. The amino group of compound (63) underwent nucleophilic substitution with cyanuric chloride (2,4,6-trichloro-1,3,5-triazine). This reaction selectively replaced one chlorine atom of the triazine ring with the amino group, yielding the final DR dye, a dichlorotriazinyl derivative (DR28), as a deep yellow powder after recrystallization (yield 94%). The two remaining chlorine atoms on the triazine ring serve as reactive sites capable of forming covalent bonds with nucleophilic groups on fibers.^60^

**Scheme 26:**
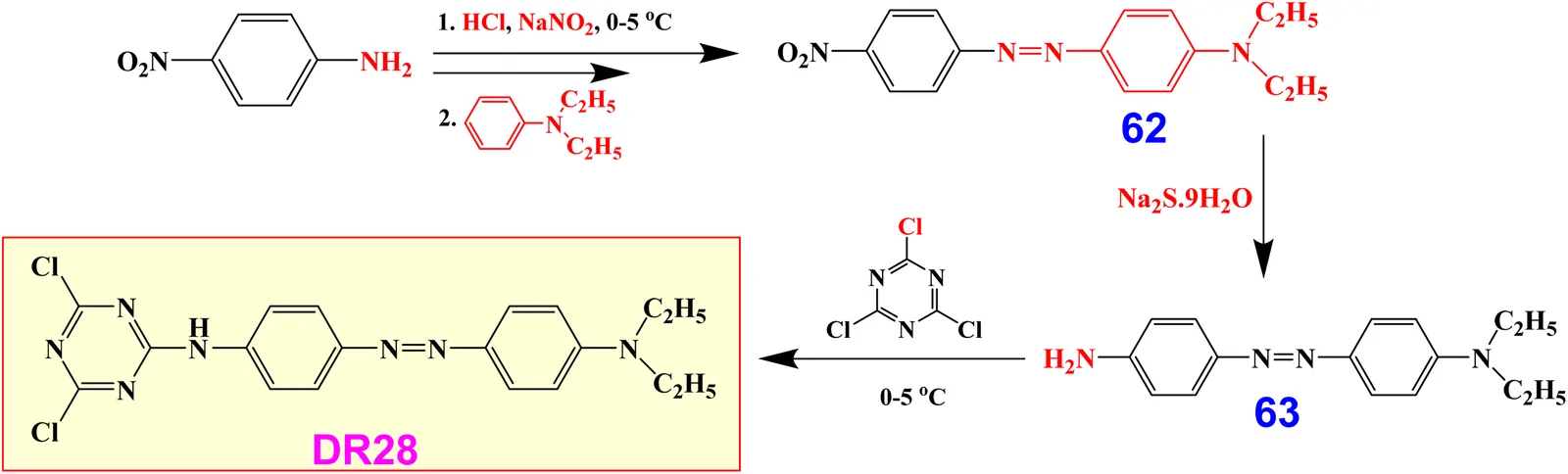
Three-step synthesis of DR28 dye *via* azo coupling, nitro reduction, and cyanuric chloride substitution.^60^

The synthesis of dye DR29 follows a sequential three-step strategy similar to that of DR28, with the key difference being the replacement of the coupling component (Scheme 27). In this case, 3-methyl-1-phenyl-5-pyrazolone is employed in place of *N*,*N*-diethylaniline, leading to a distinct chromophoric system. Following diazotization, 4-nitroaniline was coupled with 3-methyl-1-phenyl-5-pyrazolone. The coupling reaction occurred at the reactive methylene or methine position of the pyrazolone ring, producing the monoazo dye (64) as an orange-red powder. Compound (64) was reduced using sodium sulfide. The nitro group was selectively converted to a primary amine, and after concentration of the combined organic phases, the amino-functionalized azo dye (65) was obtained as a red powder. The amino group of compound (65) underwent nucleophilic substitution with cyanuric chloride (2,4,6-trichloro-1,3,5-triazine). One chlorine atom of the triazine ring was selectively replaced by the amine, yielding the final dichlorotriazinyl DR dye, DR29 (yield 91%).^60^

**Scheme 27:**
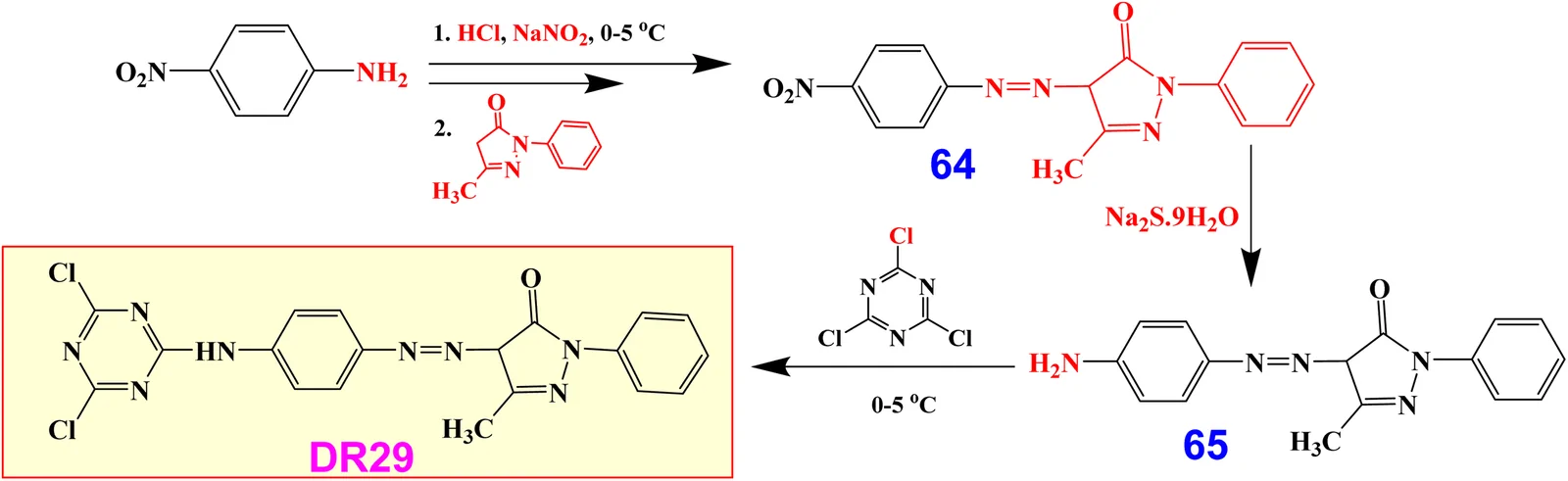
Three-step synthesis of DR29 dye *via* pyrazolone azo coupling, nitro reduction, and triazine functionalization.^60^

The synthesis of dye DR30 follows a two-step sequential strategy, commencing with a commercially available azo dye—4-(4-nitrophenylazo) aniline (Disperse Orange 3, 66) rather than proceeding *via* diazotization and coupling (Scheme 28). This approach offers an alternative and more direct route for converting a conventional disperse dye into a DR dye. 4-(4-Nitrophenylazo)aniline was subjected to reduction using Na_2_S·9H_2_O. The nitro group on the diazo component was selectively reduced to a primary amine. The combined organic phases were concentrated and dried to yield the amino-functionalized azo dye (67) as a deep yellow powder. This product contains two amino groups: one originally present on the coupling component and the other newly generated on the diazo component. Compound (67) was added dropwise to a solution of cyanuric chloride in 1,4-dioxane. Notably, two equivalents of cyanuric chloride were employed, indicating that both amino groups (on the diazo and coupling components) underwent nucleophilic substitution reactions. One chlorine atom on each triazine ring was selectively replaced by the respective amine group. Upon completion, dye DR30 was obtained as a yellow powder (yield 90%).^60^

**Scheme 28:**
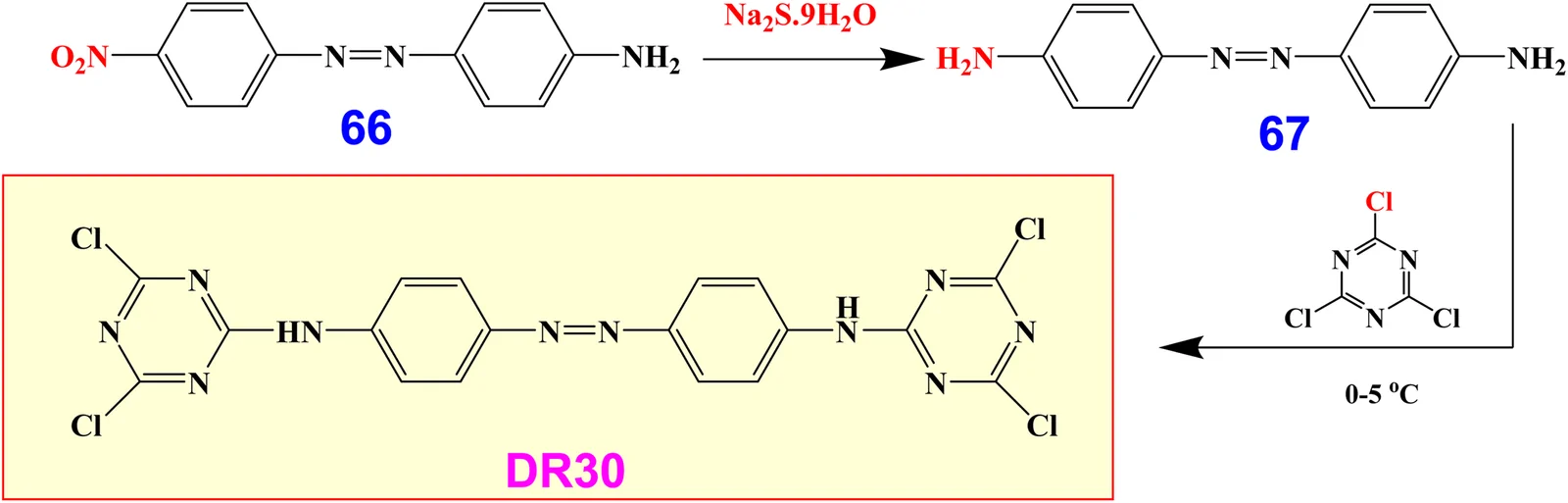
Two-step synthesis of DR30 dye *via* nitro reduction of Disperse Orange 3 and bis-triazine functionalization.^60^

Scheme 29 presents a modular two-step synthetic approach for preparing a series of DR dyes incorporating a rhodanine core (2-thioxothiazolidin-4-one), a reactive dichlorotriazinyl group, and an arylazo chromophore. 3-Amino-2-thioxothiazolidin-4-one (68) underwent a condensation reaction with cyanuric chloride. The reaction is carried out by first stirring cyanuric chloride in acetone and water at below 5 °C for 1 hour. Then, a neutral solution of 3-amino-2-thioxothiazolidin-4-one in aqueous sodium carbonate solution is added in small portions over 1 hour. The pH is kept neutral by adding sodium carbonate solution. The reaction mixture is continuously stirred at 0–5 °C for 4 hours until a clear solution is formed. This reaction selectively replaced one chlorine atom of the triazine ring *via* nucleophilic attack by the amino group of the rhodanine derivative, yielding the cyanurated intermediate 3-((4,6-dichloro-1,3,5-triazin-2-yl)amino)-2-thioxothiazolidin-4-one (69). The resultant solution is used for the next step without further purification. Intermediate (69) served as the coupling component in a series of diazo coupling reactions. Aromatic amines were conventionally diazotized: the aromatic amine in HCl is cooled in an ice bath at 0–5 °C, and NaNO_2_ solution is added dropwise over 30 minutes, followed by stirring for another 1 hour. Completion of diazotization is confirmed by starch-iodide paper. Excess nitrous acid is destroyed by adding urea. The resulting diazonium salts were coupled with compound (69) by slow addition over 30 minutes in ethanol buffered with sodium acetate at 0 °C for 2 hours. Coupling occurred at the active methylene position (C-5) of the rhodanine ring. The resulting colored solid is filtered, repeatedly washed with cold water, dried, and crystallized from ethanol, producing the final dyes: 3-((4,6-dichloro-1,3,5-triazin-2-yl)amino)-5-(aryldiazenyl)-2-thioxothiazolidin-4-one (DR31; yield 78%).^61^

**Scheme 29:**
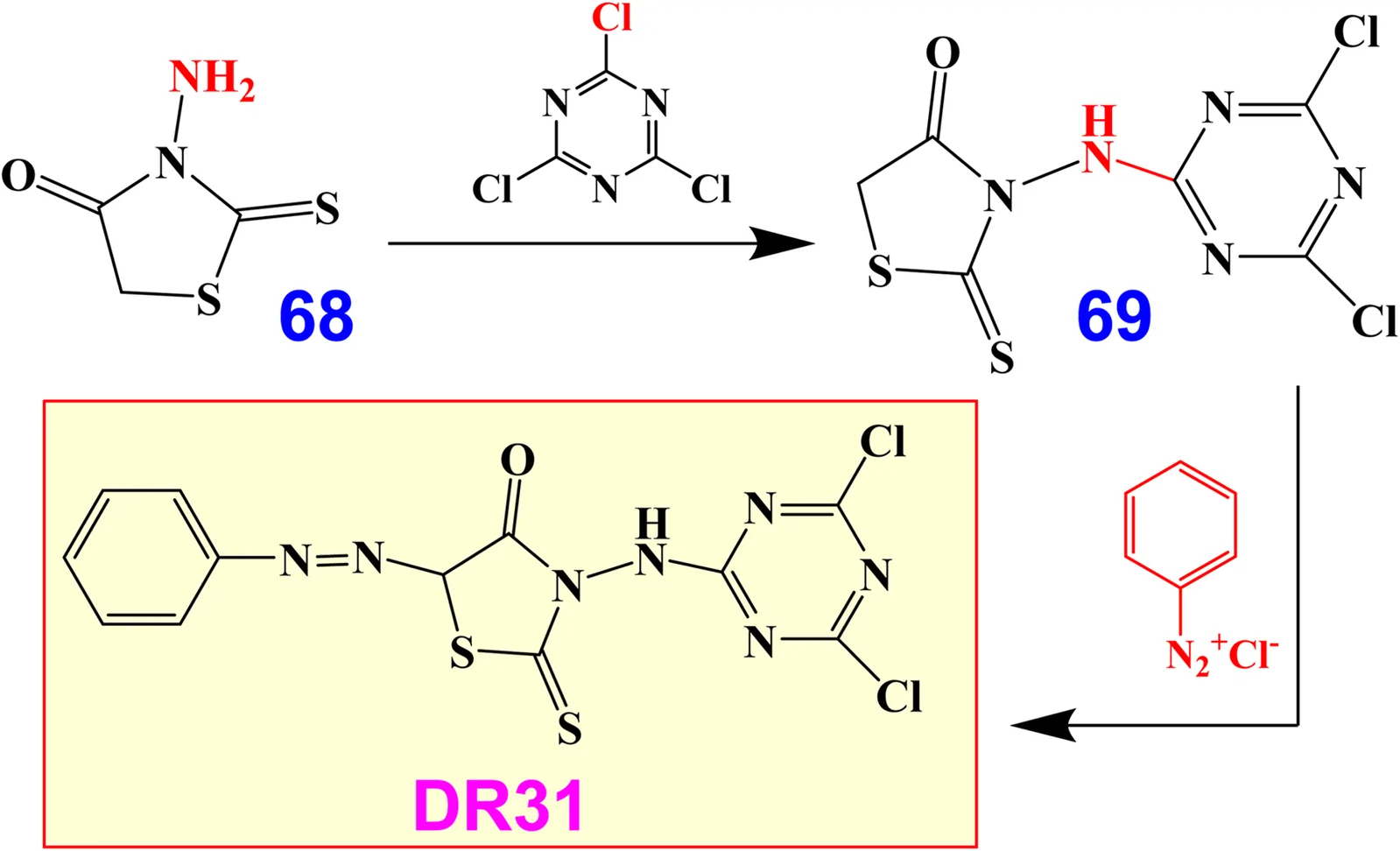
Modular two-step synthesis of DR31 dyes *via* rhodanine–triazine condensation and diazo coupling.^61^

While Section 3.1 summarizes the reported synthetic approaches and reaction conditions for the investigated DR dyes, a comparative evaluation of their reactive functionalities is necessary to identify broader trends beyond individual synthetic procedures. Accordingly, Section 3.2 classifies the reported dyes according to their reactive groups and examines the differences in reactivity, hydrolytic behavior, and reported synthetic accessibility, thereby providing a more structured basis for comparing their chemical and practical characteristics.

### 3.2. Classification of DR dyes by reactive functional groups

Researchers have reported synthesizing DR dyes (ranging from DR1 to DR31) using various methods. To facilitate comparison, a classification framework based on the reactive functional groups of these dyes has been developed. These groups can be categorized into seven classes: (i) dichlorotriazine, (ii) monochlorotriazine, (iii) vinyl sulfone, (iv) haloacyl, (v) acyl fluoride, (vi) epoxide, and (vii) sulfonamide. This framework enables comparison of key parameters, such as synthesis temperature, solvent, yield, reactivity, and hydrolytic stability, and helps select suitable reactive groups for waterless dyeing applications. Table 6 presents a comprehensive classification of all reported DR dyes according to their reactive functional groups. The table includes the dye codes, chromophore types, synthesis temperature, solvent, yield, and number of synthetic steps for each group.

**Table 6:** Classification of DR dyes by reactive functional groups

Reactive group	Dye codes	Chromophore type	Synthesis temp.	Solvent	Yield (%)	Steps
Dichlorotriazine	DR1, DR2, DR5–DR8, DR10, DR12, DR14, DR15, DR19, DR20, DR26–DR31	Thiazole-azo, anthraquinone, azo, benzothiazole-pyrazolone, pyrrolocyanine, rhodanine	0–50 °C	Toluene, dioxane, DCM, DMF, acetic acid	25–98	1–5
Monochlorotriazine	DR21	Anthraquinone	Ambient	N.R.	65	2
Vinyl sulfone	DR9, DR13, DR16, DR18, DR22, DR25	Thiophene-hydrazine, thiazole-azo, azo, naphthol-azo, pyrazolone-azo, pyrrolinone-azo	0–5 °C	Ethanol, DMF, –alkaline	70–89	1–2
Haloacyl	DR3a, DR3b, DR3c, DR24	Azo, anthraquinone, thiazole-azo	0 °C	DCM, DMF	82–93	1–2
Acyl fluoride	DR17a, DR17b	Azo	0 °C	HF–pyridine	N.R.	2
Epoxide	DR23	Azo	60–80 °C	N.R.	73	3
Diazo-reactive-group	DR4	Diazo	0 °C	DCM, acetonitrile	85	2

The seven reactive groups exhibited distinct profiles in terms of reactivity, hydrolytic behavior, and reported synthetic accessibility. Their relative suitability depends on the dye structure and processing conditions rather than on a universally optimal reactive functionality.

Dichlorotriazine was the most frequently represented group, occurring in 19 of 35 derivatives (54%). Its high reactivity toward nucleophilic fiber sites is accompanied by relatively limited hydrolytic stability, requiring careful control of reaction conditions during synthesis and application. Its widespread use is likely associated with the established chemistry and commercial availability of cyanuric chloride. However, its susceptibility to hydrolysis may limit its suitability for prolonged aqueous processing and harsh alkaline conditions.

Vinyl sulfone accounted for seven derivatives (20%) and showed a comparatively balanced profile in the present dataset. The masked β-sulfatoethylsulfone precursor is commonly converted into the active vinyl sulfone species under alkaline conditions, enabling covalent fixation onto cellulose. The reported syntheses generally involved one or two steps, with yields of 70–89%, indicating favorable synthetic accessibility. Nevertheless, the activated vinyl sulfone group remains susceptible to competing hydrolysis under alkaline conditions.

Haloacyl groups represented four derivatives (11%) and were associated with relatively simple synthetic procedures, including one-step preparations with reported yields of 82–93%. Their reactivity and hydrolytic behavior, however, depend on the specific haloacyl structure and should therefore be evaluated on an individual basis.

Acyl fluoride, epoxide, monochlorotriazine, and sulfonamide functionalities were represented by two, one, one, and one derivatives, respectively. Acyl fluoride derivatives may offer high electrophilic reactivity but are generally moisture-sensitive, which complicates their synthesis and handling. The limited number of examples and incomplete yield reporting prevent firm conclusions regarding their overall synthetic or application advantages. The epoxide-containing example required elevated synthesis temperatures of 60–80 °C, while its broader performance remains insufficiently established. Monochlorotriazine is generally less reactive but more hydrolytically stable than dichlorotriazine, whereas the limited data for sulfonamide-containing dyes do not support broader comparative conclusions.

Overall, reactive group selection should consider the balance between reactivity, hydrolytic behavior, synthetic accessibility, fiber type, and processing conditions. Although dichlorotriazine predominates in the examined literature, vinyl sulfone and haloacyl groups offer alternative reactive profiles. However, the practical performance of DR dyes also depends on their compatibility with the dyeing medium and their ability to interact effectively with the target fiber. Accordingly, the following section examines five major waterless dyeing platforms, SC-CO_2_, solvent-assisted systems, reverse micelle methods, ionic liquids/deep eutectic solvents (ILs/DES), and inkjet digital printing—with emphasis on reported experimental evidence, dyeing performance, and remaining challenges.

## 4. Waterless dyeing-based methods

The textile industry, one of the most water-intensive sectors and a major source of freshwater pollution, is undergoing a structural transition driven by increasing global demand and mounting environmental pressures. This transition progresses from optimizing conventional processes to implementing fundamentally transformative technologies.^62^ The initial phase emphasizes low-water or reduced-liquor technologies, which significantly decrease, but do not eliminate, water consumption in traditional dyeing. Representative approaches include foam dyeing, where foam functions as the dye carrier, ultrasonic-assisted dyeing to enhance dye diffusion, low-liquor-ratio machinery, and the development of dyes and auxiliaries requiring minimal rinsing. These strategies provide practical, incremental solutions that reduce environmental impact while remaining compatible with existing industrial infrastructure.^11^ A more advanced stage involves the complete removal of water from the dyeing process. In such systems, the fundamental physicochemical framework of dyeing is redefined, and conventional aqueous dye baths containing large volumes of salts and auxiliaries are eliminated.^63^ Waterless dyeing technologies offer substantial advantages. Water consumption can be reduced to nearly zero in certain systems, making them particularly relevant for water-scarce regions.^64^ Energy demand is lowered due to the elimination or significant reduction of the drying stage.^11^ Furthermore, the absence of saline and alkaline wastewater streams minimizes environmental burdens and reduces treatment costs.^63^ These systems can also enhance process efficiency, shorten cycle times, improve color uniformity, and enable dye recovery, generating economic benefits.^11^

### 4.1. Solvent-assisted dyeing

Solvent-assisted dyeing replaces water with other organic solvents that can be recovered and reused in the dyeing process for improving resource efficiency (Fig. 4).^12,65^ Solvent selection depends on dye chemistry and solvent–fiber compatibility. Water-soluble dyes (*e.g.*, direct, acid, reactive) are typically processed in polar solvents, whereas hydrophobic dyes like disperse dyes prefer nonpolar media. Beyond compatibility, critical factors include toxicity, volatility, flammability, flash point, explosion potential, and solvent recovery/reuse. At the fiber level, solvent–fiber interactions affect surface tension, swelling, dye penetration, and uniformity. An ideal solvent causes no negative impact on fiber properties or fastness. These aspects collectively ensure safety and sustainability.^65,66^ In compatible systems, organic solvents can induce controlled fiber swelling, increase polymer chain mobility, and expand the free volume of the polymer matrix. These effects reduce the activation energy for dye diffusion and improve the dye–fiber partition coefficient, leading to faster dye uptake and shorter processing times compared with aqueous systems. The main advantage of this technique is operation at very low liquor ratios, which significantly reduces water consumption and eliminates the need for large amounts of electrolytes and auxiliaries.^67^

**Fig. 4:**
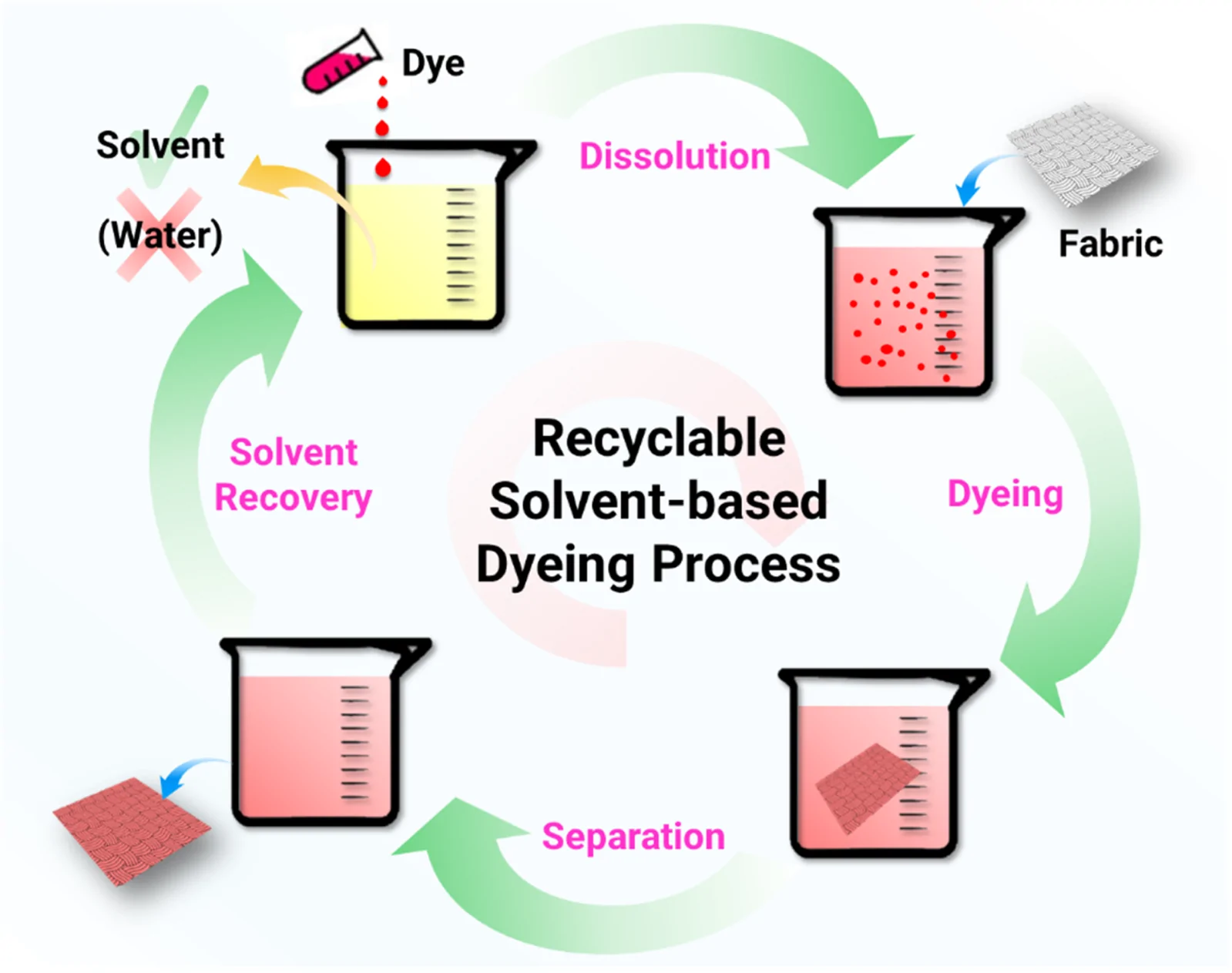
Conceptual diagram of solvent-assisted dyeing with solvent recovery.

A wide range of alternative solvents has been investigated as potential media for solvent-assisted and waterless dyeing processes. These include short-chain alcohols, such as ethanol (EtOH), isopropyl alcohol (IPA), and propanol (PrOH); polar aprotic solvents, including dimethyl carbonate (DMC) and dimethyl sulfoxide (DMSO); and nonpolar or low-polarity organic solvents, such as carbon tetrachloride (CTC) and perchloroethylene (PCE). In addition, amine-based solvents like triethanolamine (TEA), as well as silicone-based fluids, including decamethylcyclopentasiloxane (D5) and linear polydimethylsiloxane (PDMS) fluids, have been explored as alternative dyeing media (Table 7).^65,67–70^ These solvents differ significantly in polarity, viscosity, and solvating capability, which in turn influences dye solubility, fiber swelling behavior, and dye–fiber interactions during the dyeing process. Consequently, the selection of an appropriate solvent plays a critical role in determining dyeing efficiency, color uniformity, and the overall environmental performance of solvent-assisted textile coloration systems. Among these, D5 has received particular attention due to its low surface tension, chemical stability, and recyclability in closed-loop systems.

**Table 7:** Summary of solvent-assisted dyeing of various textile fibers

Fiber	Dye		Medium/solvent	owf%	L : R	Temp./time	K/S	Color fastness (wash/rub/light)	Ref.
Cotton	C.I. Reactive Red 195	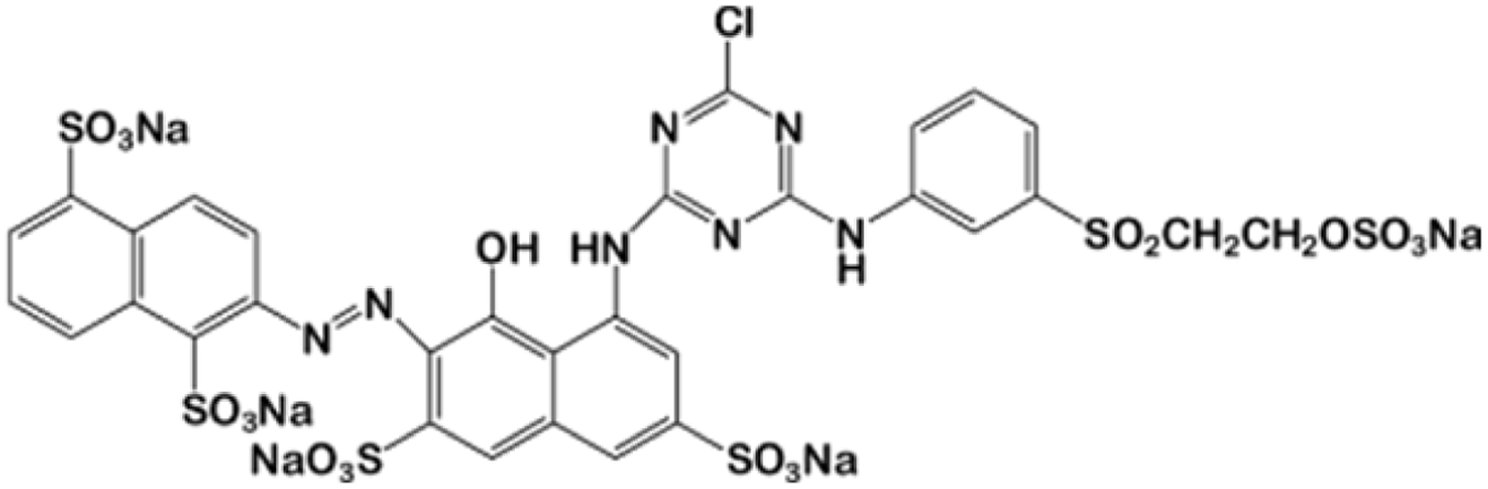	EtOH/CTC/H_2_O	2	1 : 20	60 °C – 20 min	10.70	5/5/N.P.	68
Cotton	C.I. Reactive Yellow 145	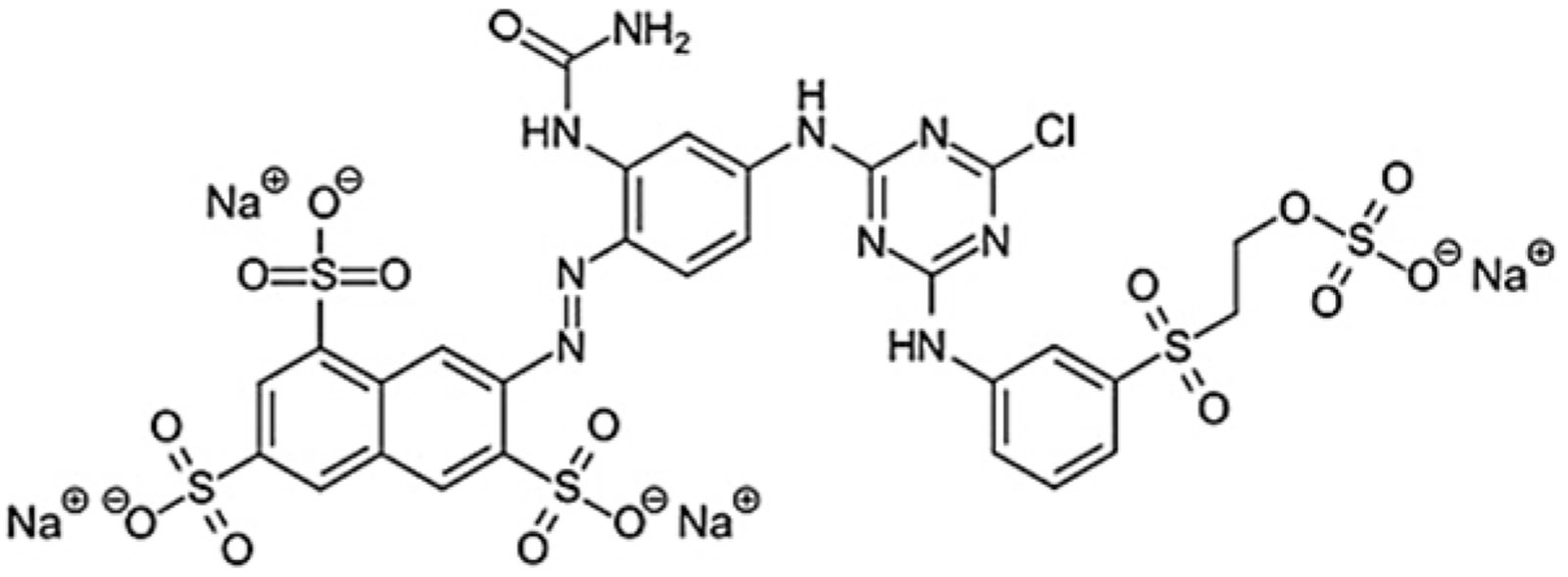	EtOH/CTC/H_2_O	2	1 : 20	60 °C – 20 min	10.45	4–5/4–5/N.P.	68
Cotton	C.I. Reactive Blue 194	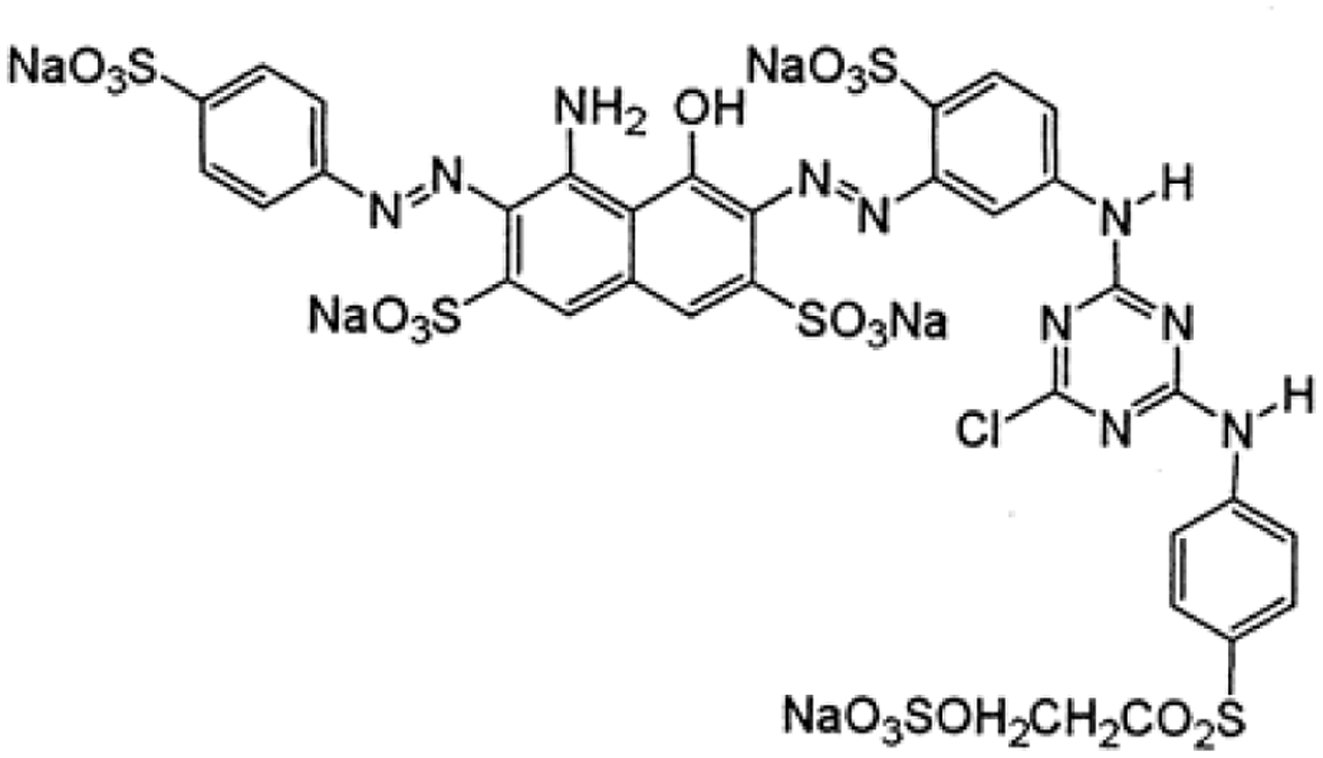	EtOH/CTC/H_2_O	2	1 : 20	60 °C – 20 min	19.26	4–5/5/N.P.	68
Cotton	C.I. Reactive Red 239	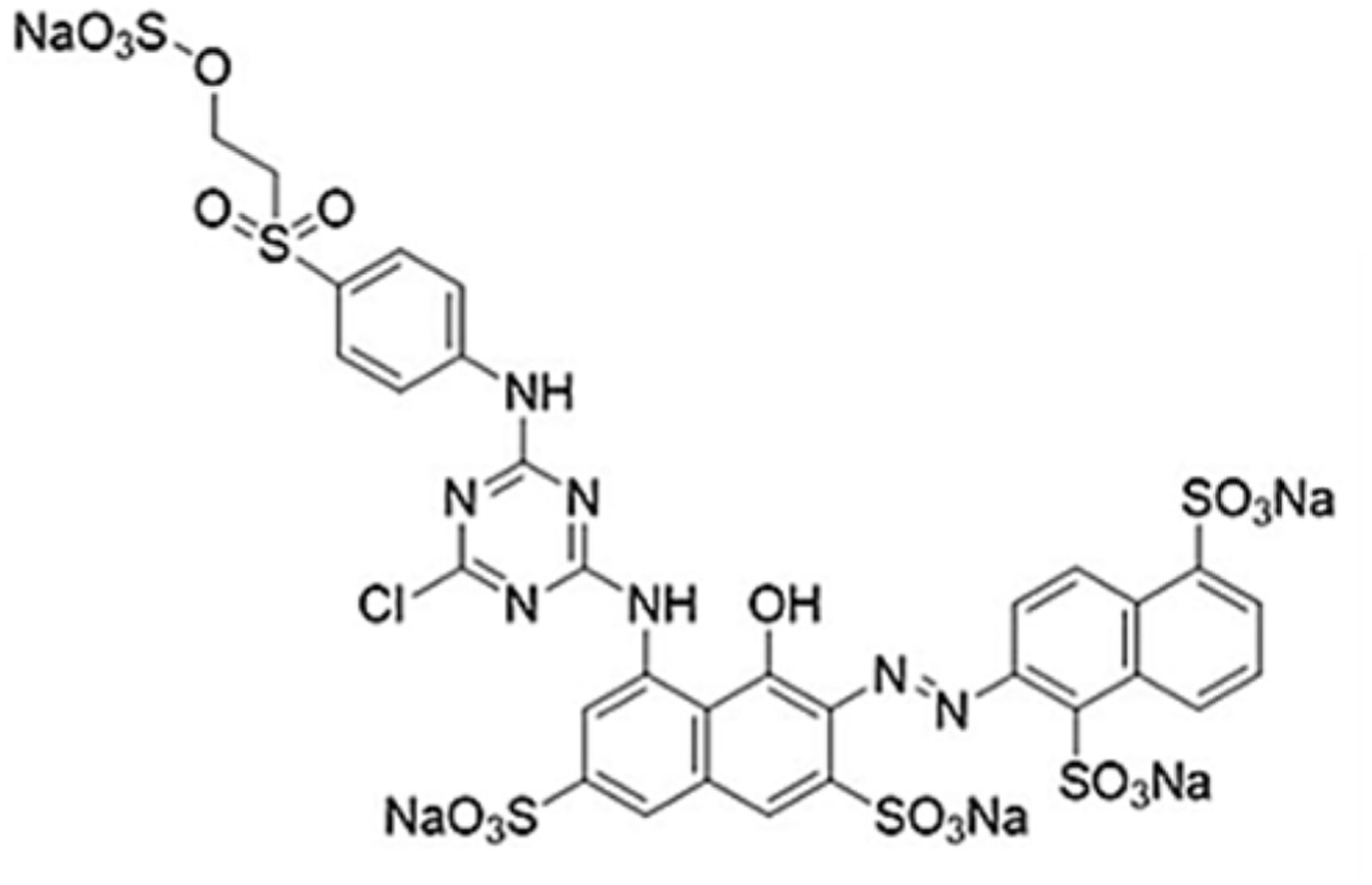	EtOH	1	1 : 20	60 °C – 60 min	6.14	4/N.P./3	65
Cotton	C.I. Reactive Yellow 6	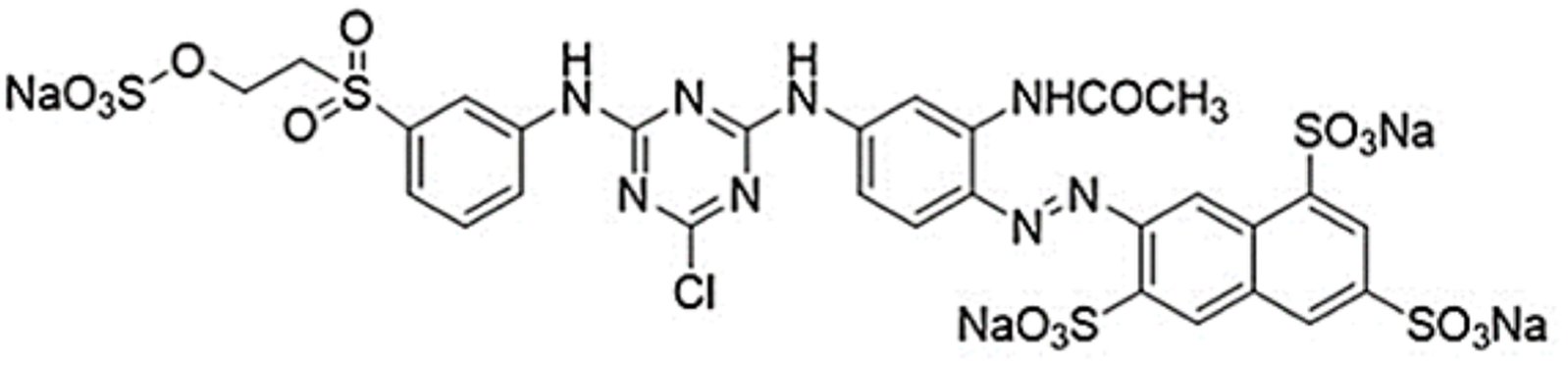	IPA	1	1 : 20	60 °C – 60 min	6.59	4–5/N.P./6	65
Cotton	C.I. Reactive Blue 220	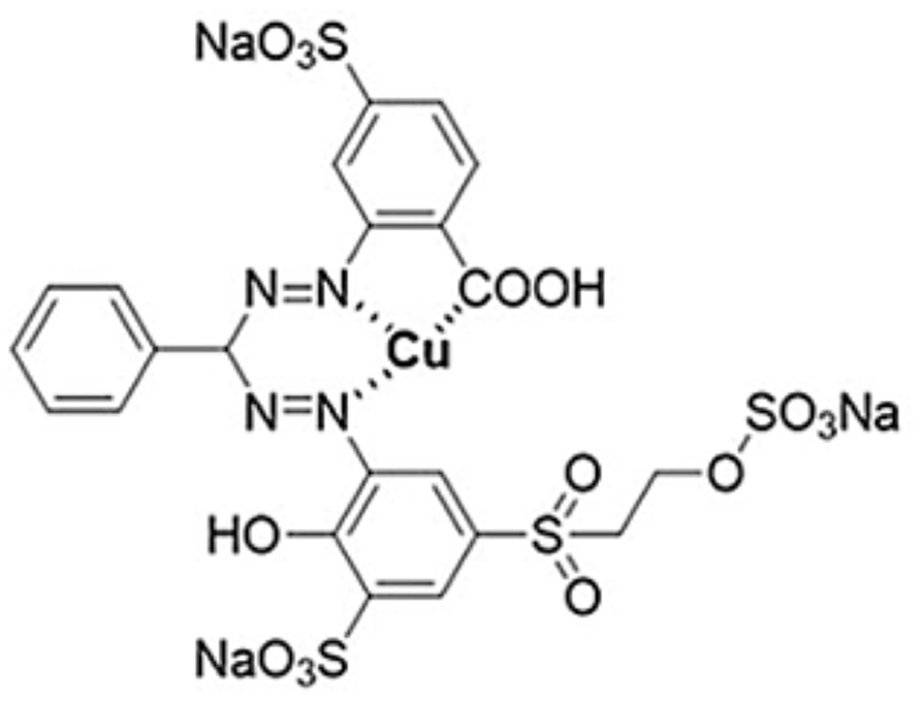	IPA	1	1 : 20	60 °C – 60 min	12.98	4/N.P./6	65
Wool	Lanasol Red CE	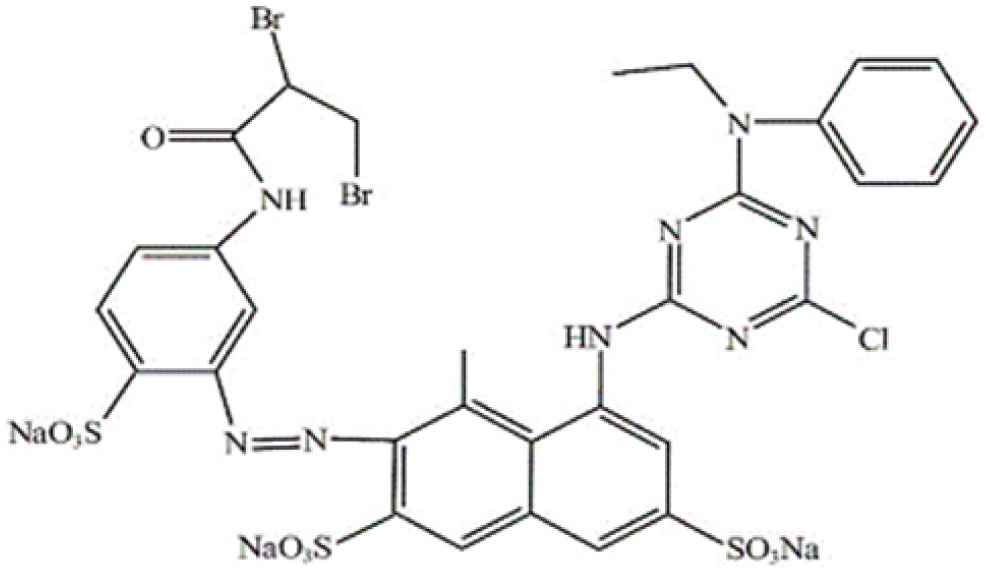	EtOH/CTC/H_2_O	2	1 : 50	30 °C to 80 °C at 2 °C min^−1^	20.43	4–5/5/N.P.	71
Wool	Madder Red	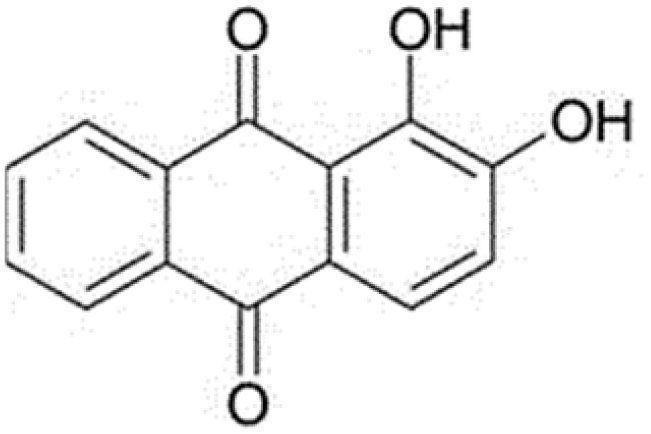	D5	2	1 : 25	90 °C – 15 min	18	5/N.P./N.P.	72
Wool	Gardenia Blue	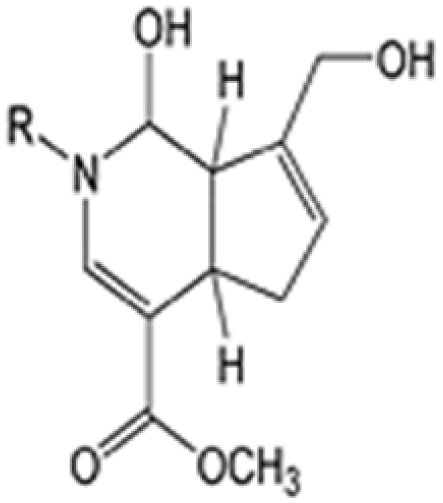	D5	2	1 : 25	90 °C – 15 min	4	5/N.P./N.P.	72
Wool	Gardenia Yellow	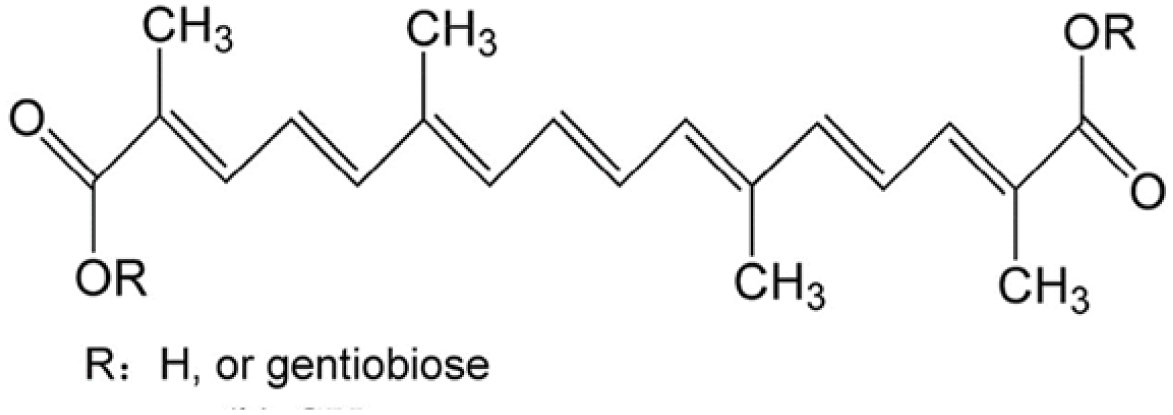	D5	2	1 : 25	90 °C – 15 min	3	5/N.P./N.P.	72
Nylon	C.I. Acid Red 249	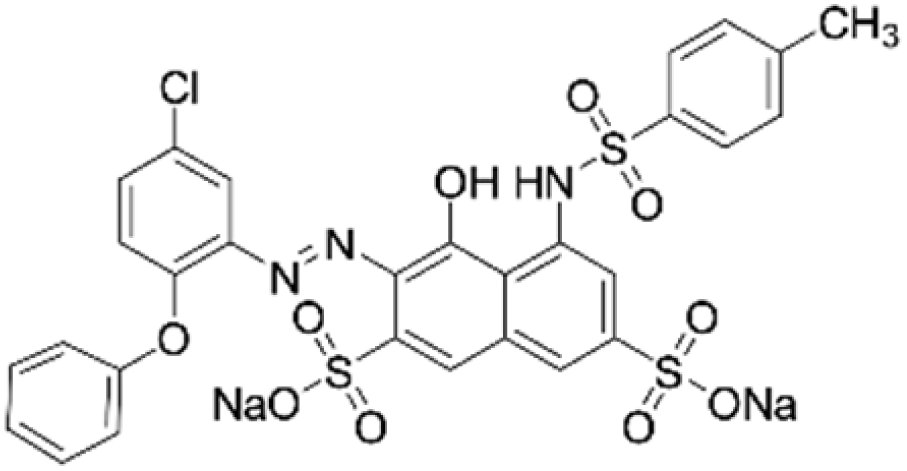	D5	2	1 : 20	98 °C – 1 h	20	4–5/4–5/N.P.	70
Nylon	C.I. Acid Blue 83	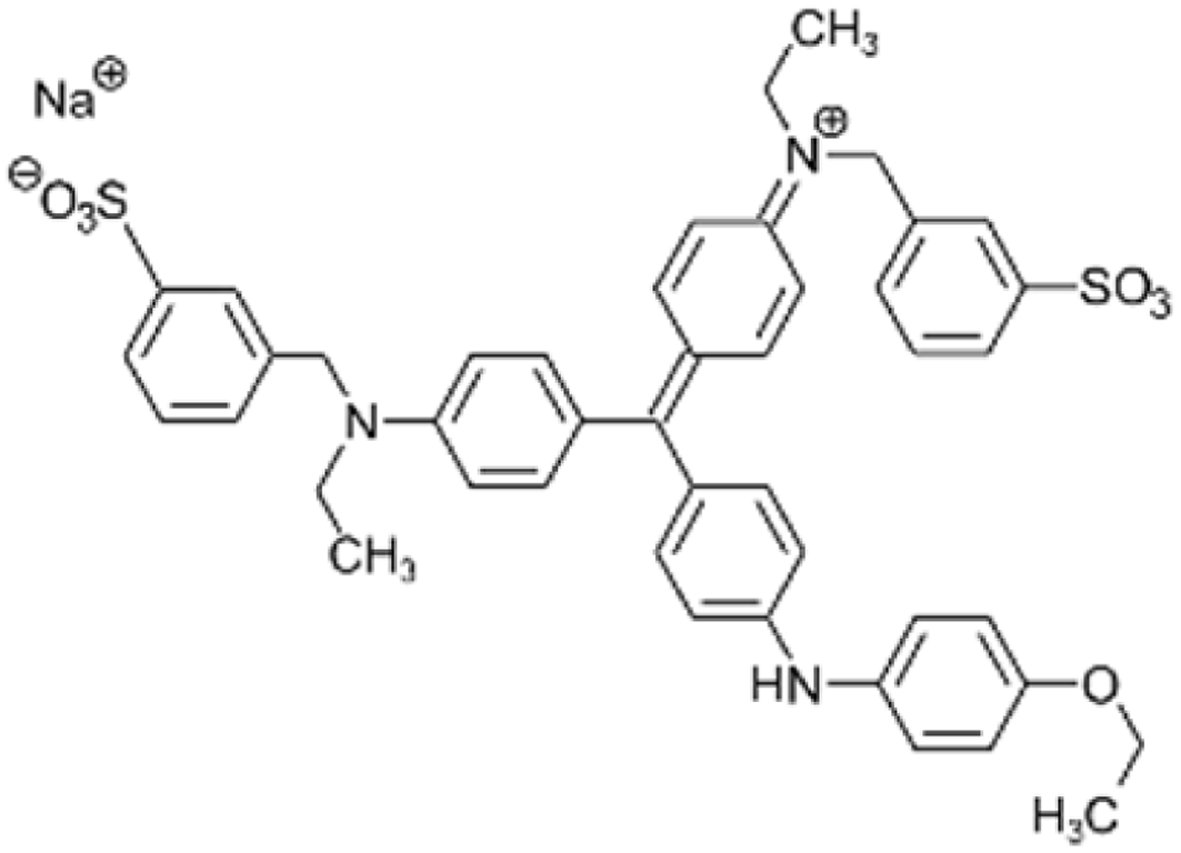	D5	2	1 : 20	98 °C – 1 h	41	4–5/4–5/N.P.	70
Nylon	C.I. Acid Orange 67	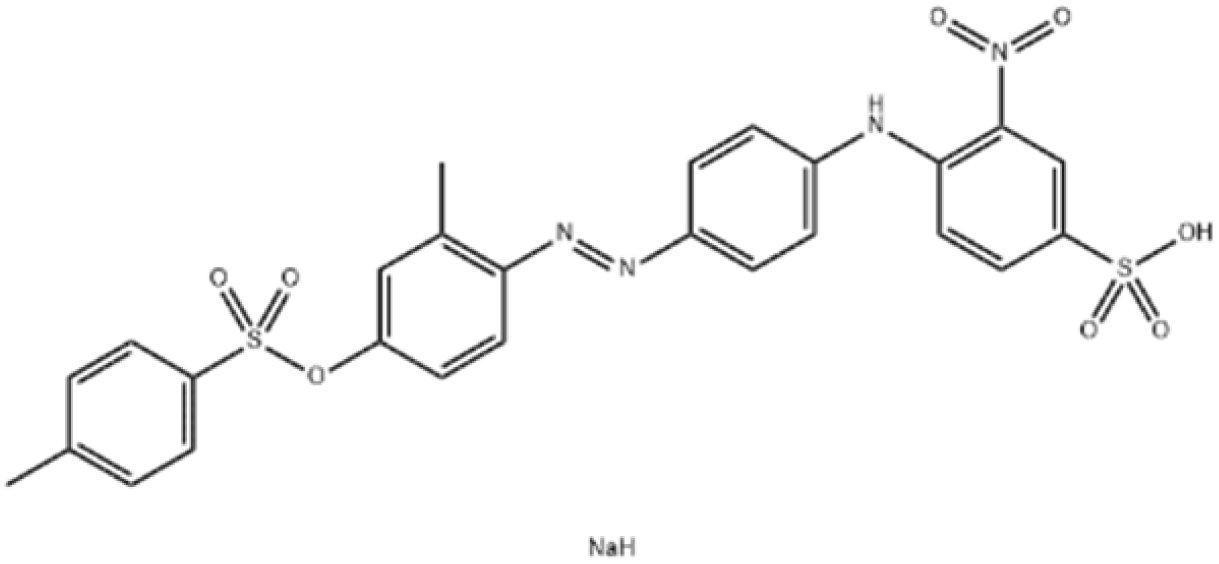	D5	2	1 : 20	98 °C – 1 h	33	4–5/5/N.P.	70
Cotton	C.I. Reactive Blue 19	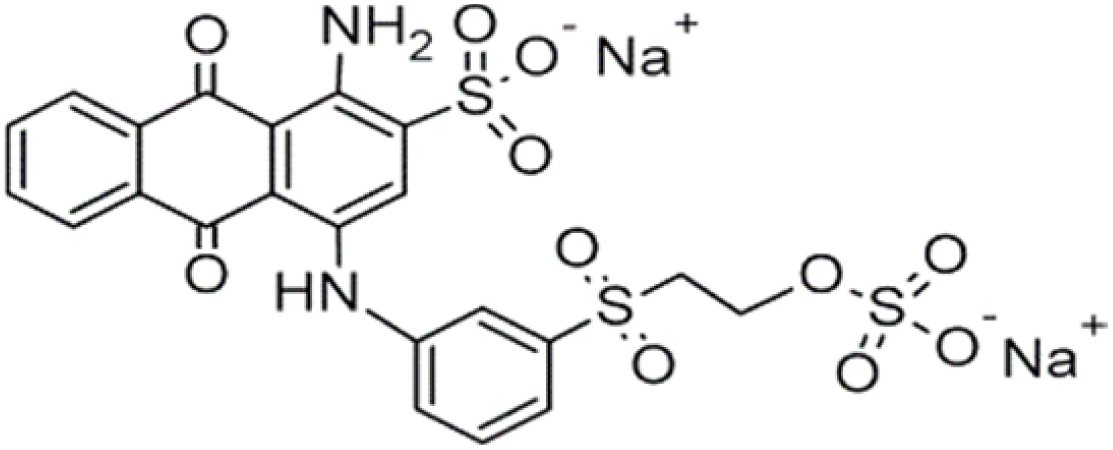	D5	2	1 : 20	25 °C to 80 °C at 2 °C min^−1^; hold 80 °C for 30 min	16	N.P.	73
Polyester	C.I. Disperse Red 167	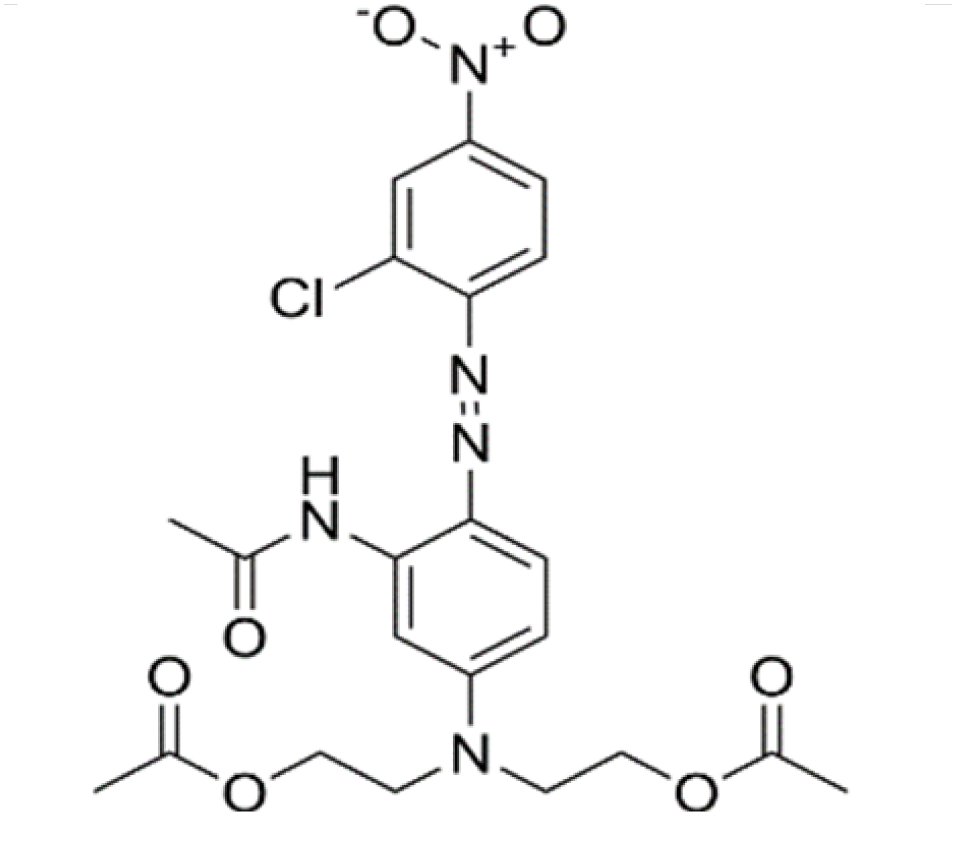	D5	1	1 : 25	30 °C to 125 °C at 3 °C min^−1^; hold 125 °C for 60 min	10.89	N.P./5/N.P.	74

Table 7 provides a summary of dyeing processes using organic solvents with various dyes (other than the DR series). The data indicate that solvent-assisted dyeing generally enhances dye color strength (K/S) across different fibers compared to conventional aqueous methods. Both cotton and synthetic fibers achieve satisfactory color fastness, with solvent systems facilitating more uniform and efficient dye penetration. Overall, the choice of solvent and dyeing conditions plays a key role in optimizing performance.

Because many DR dyes are inherently hydrophobic, they are well suited to nonpolar solvent systems such as D5. Their structure, combining a disperse chromophore with a reactive group, enables effective transport in hydrophobic media and potential covalent fixation on fibers containing nucleophilic sites after dye transfer.^29^ To date, only one study has reported their application in D5-based dyeing.^56^ In this study, cotton fabrics were dyed with DR23 dyes (Scheme 22) in a D5 medium under optimized conditions (dye 2% o.w.f., 130 °C, 80 min), achieving maximum color strength (K/S = 4.2). The dyed fabrics exhibited satisfactory fastness, with wash-, rub-, and light-fastness grades of 4–5, likely due to strong covalent bonding between the reactive groups and the cotton fibers. Dyeing was performed at a D5 to fabric ratio of 20 : 1, with initial heating from 40 °C to 130 °C at a rate of 1 °C min^−1^. These findings demonstrate that waterless dyeing in D5 is a viable approach for DR dyes, enabling efficient fiber fixation and excellent fastness performance.^56^ The presence of reactive functional groups in DR dyes facilitates covalent bonding with cellulosic fibers while preserving sufficient hydrophobic character for diffusion in non-aqueous media, thereby enhancing fixation efficiency and dyeing performance in waterless systems.

Despite the promising performance of solvent-assisted dyeing systems (Table 7), several important limitations remain that currently restrict their widespread industrial adoption. One major challenge is the high cost of many organic solvents, particularly when compared with water, which remains an inexpensive and readily available processing medium. In addition, certain solvents investigated for textile dyeing, such as carbon tetrachloride (CTC) and perchloroethylene (PCE), raise significant environmental and health concerns due to their toxicity. Many organic solvents are flammable, and they may emit volatile organic compounds (VOCs), which require strict handling protocols, closed processing systems, and advanced ventilation to meet occupational and environmental safety standards.

Another critical consideration is the need for efficient solvent recovery and recycling systems. To ensure economic viability and minimize environmental impact, solvent-based dyeing processes must operate in closed-loop configurations with high solvent recovery efficiencies. Without effective recovery, solvent losses can significantly increase operating costs.

Consequently, the true sustainability of solvent-assisted textile dyeing cannot be evaluated solely based on reduced water usage. Instead, the overall environmental and economic performance must be assessed through comprehensive life-cycle analyses (LCA) that consider solvent production, energy consumption, recovery efficiency, and potential emissions throughout the process chain.^75^ Addressing these challenges through improved solvent design, safer chemistries, and efficient recovery technologies will be essential for enabling solvent-based dyeing systems to achieve broader industrial implementation.

### 4.2. Reverse micelle dyeing

The reverse micelle dyeing system is a promising low-water dyeing method and a sustainable alternative to conventional water-based dyeing (Fig. 5).^76^ Initially developed for cotton, it has been extended to other natural fibers. In this solvent-mediated process, dyes are dissolved in a small amount of water, encapsulated within reverse micelles of a surfactant, and dispersed in an organic solvent.^77^ The fiber is dyed as the dye-containing water pools transfer to its surface, while micelles remain in the solvent. Reverse micelles are nanoscale water droplets stabilized by surfactants in nonpolar solvents. The polar heads surround the aqueous dye pools, and hydrophobic tails extend into the solvent.^78^ The water-to-surfactant molar ratio (*W*_0_ = [H_2_O]/[surfactant]) controls pool size and dye solubility; excessive *W*_0_ can destabilize the system.^76^

**Fig. 5:**
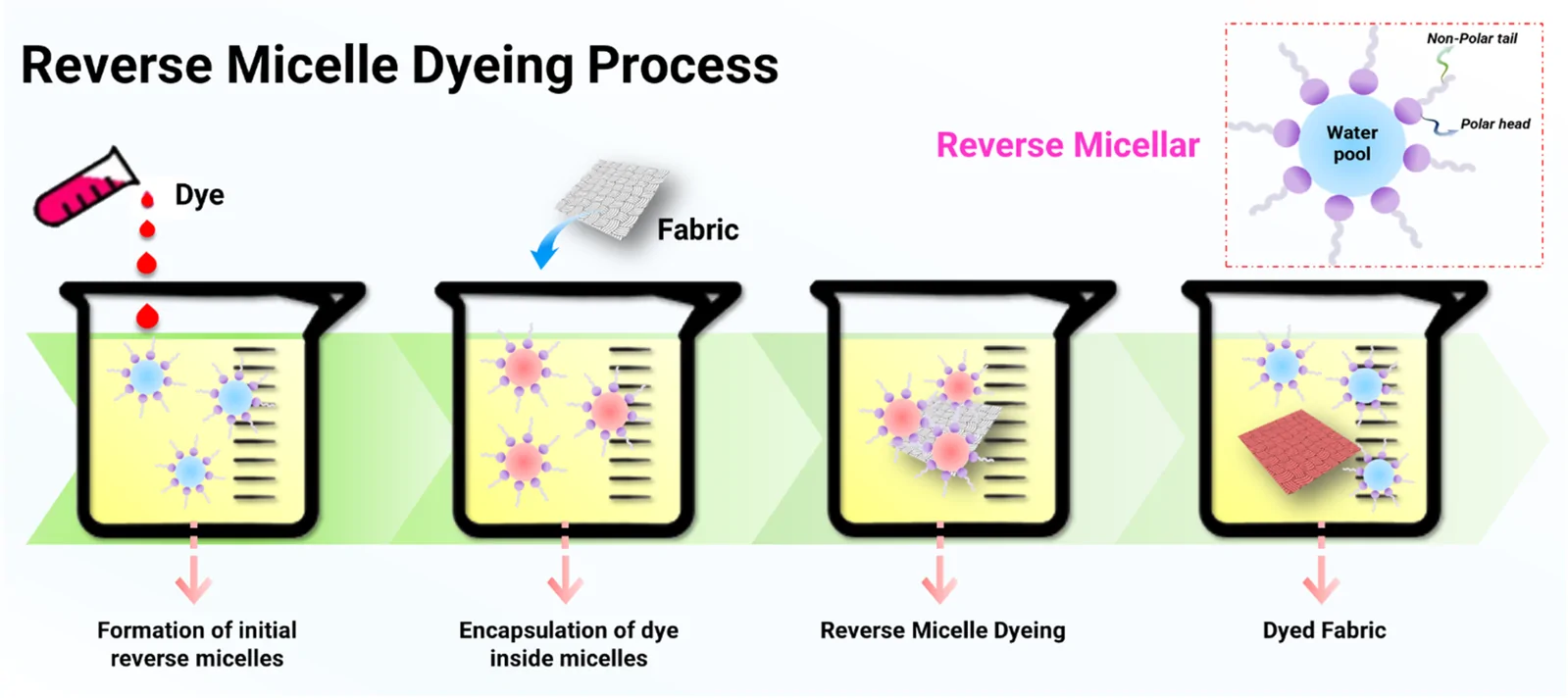
Schematic of the reverse micelle dyeing system.

Surfactant selection plays a critical role in reverse micellar dyeing, as it significantly influences color yield, dye uniformity, and fastness properties by controlling micelle formation, stability, and dye transport. A variety of surfactants have been employed in reverse micellar systems, including anionic surfactants such as sodium bis(2-ethylhexyl) sulfosuccinate (AOT), nonionic surfactants such as MOA-3, Triton X-100 (TX-10), alkyl polyglucosides (APG), and secondary alcohol ethoxylates (SAE), as well as cationic surfactants including octadecyltrimethylammonium chloride (1831) and benzylhexadecyldimethylammonium chloride (BHDC).^79,80^ In addition, biodegradable biosurfactants have been explored as environmentally friendly alternatives.^81^

The solvent phase used in reverse micellar dyeing is equally important for determining dye solubility, micelle stability, and fiber wetting behavior. Various organic solvents have been investigated as the continuous phase, including hydrocarbon solvents such as *n*-heptane, hexane, cyclohexane, and other alkanes, as well as alternative media such as polyethylene glycol and soybean oil. Among these, decamethylcyclopentasiloxane (D5) has received particular attention as a recoverable alternative dyeing medium due to its low toxicity, odorless nature, controlled volatility, and high recyclability (>98%), making it especially suitable for closed-loop, waterless dyeing processes.^82^

Reverse micellar dyeing is primarily suited for water-soluble dyes, including acid, direct, reactive, and basic dyes, which are solubilized within the aqueous cores of reverse micelles dispersed in organic solvents. In this system, the micellar water pools provide a localized environment for dye dissolution and fiber interaction while the surrounding organic phase enhances fiber wetting and dye transport. However, for DR dyes, this approach may not be necessary. Many DR dyes possess partially hydrophobic structures, particularly those lacking sulfonate groups, and can dissolve directly in suitable organic solvents while still containing reactive functionalities such as dichlorotriazine or vinyl sulfone groups capable of covalent bonding with cellulosic fibers. Consequently, DR dyes may enable direct solvent-based dyeing without the need to introduce additional water and surfactants, thereby simplifying the dyeing medium and reducing process complexity. Although reverse micelle systems have been successfully applied to several water-soluble dye classes, including acid, direct, and basic dyes, the inherent solvent compatibility of DR dyes suggests a potentially more straightforward route for waterless dyeing processes (Table 8).

**Table 8:** Dyeing conditions for various fibers using reverse micellar systems^a^

Fiber	Dye		Solvent/emulsifier	owf%	Temp./time	K/S	Ref.
Cotton	Levafix Yellow CA		Nonane/PEG-12 + *n*-octanol (1 : 7 mole)	0.1–3.5	70 °C/40 min (dyeing) + 70 °C/60 min (fixation)	N.P.	79
Cotton	Levafix Red CA		Nonane/PEG-12 + *n*-octanol (1 : 7 mole)	0.1–3.5	70 °C/40 min + 70 °C/60 min	N.P.	79
Cotton	Levafix Blue CA		Nonane/PEG-12 + *n*-octanol (1 : 7 mole)	0.1–3.5	70 °C/40 min + 70 °C/60 min	N.P.	79
Cotton	Levafix Red CA		Heptane/PEG-12 + *n*-octanol (6 : 1, 8 : 1, 9 : 1 co-surfactant : surfactant)	6%	70 °C/105 min	34.35	76
Silk	C.I. Acid Orange 7	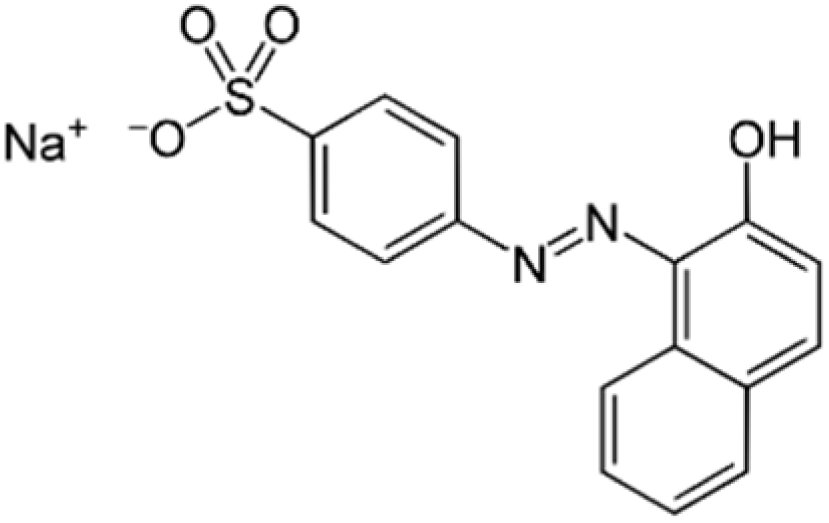	Isooctane/AOT	0–3	40 °C/24 h	3.5	80
Silk	C.I. Acid Blue 83	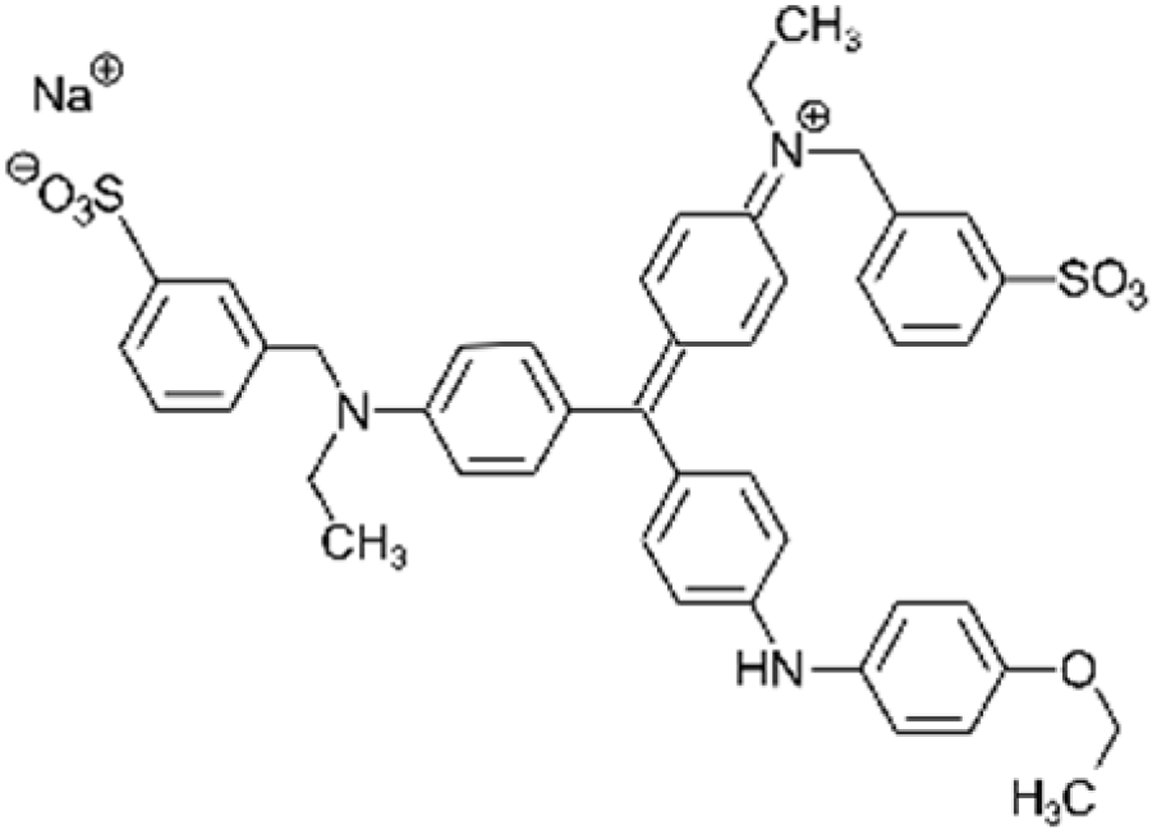	Isooctane/AOT	0–3	40 °C/24 h	12	80
Wool	C.I. Acid Blue 83	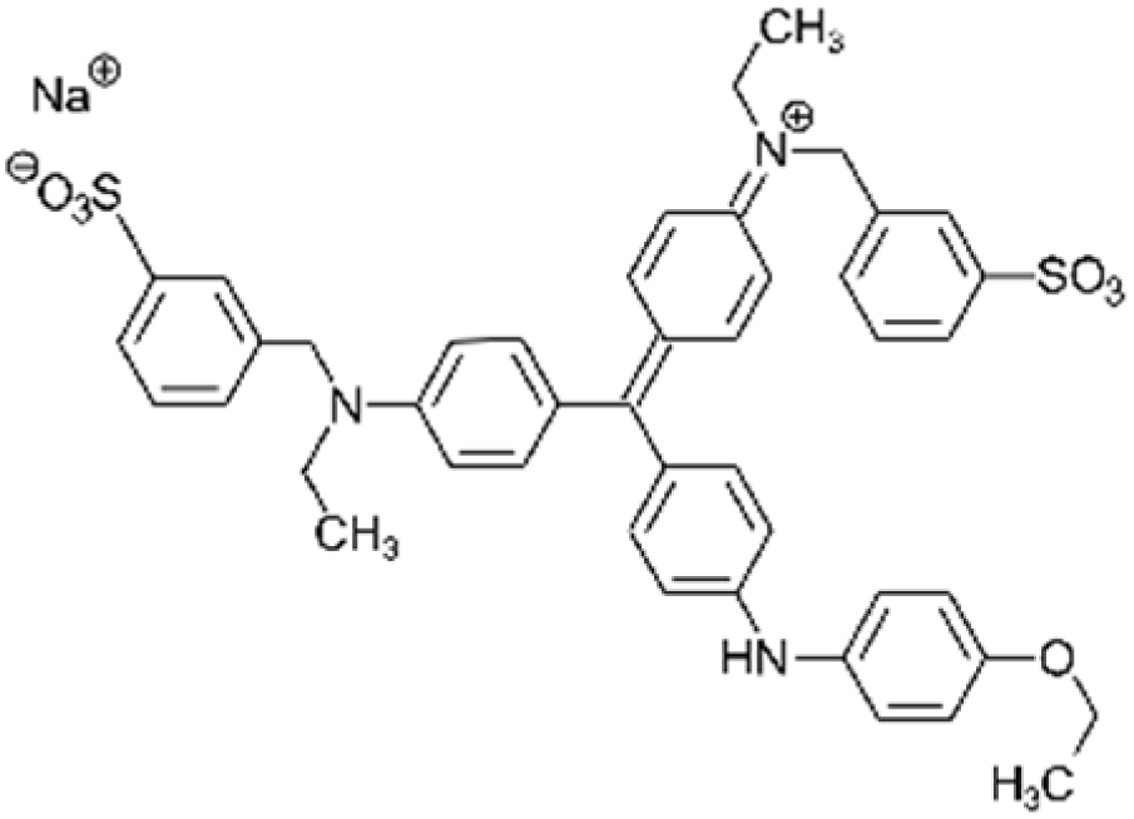	Isooctane/AOT	0–3	40 °C/24 h	7	80

a The chemical structures of Levafix CA dyes are proprietary and not disclosed by the manufacturer (Dystar); however, they are bifunctional reactive dyes widely used in industrial textile dyeing.

It should be noted that some commercially important reactive dyes, such as the Levafix CA series, have proprietary structures that are not publicly disclosed. Nevertheless, they are included in Table 8 because they represent the only reported reactive dyeing of cotton in reverse micellar systems and are widely used in industrial practice.^79^

### 4.3. Dyeing in ionic liquids (ILs) and deep eutectic solvents (DES)

ILs have emerged as promising alternative media for solvent-assisted dyeing systems due to their negligible vapor pressure, high thermal stability, tunable polarity, and strong solvating capability.^83^ These molten salts, composed of organic cations paired with organic or inorganic anions, function as non-aqueous dyeing media capable of dissolving a wide range of dyes while simultaneously interacting with fiber structures.^84^

The dye is initially dissolved or uniformly dispersed in the ILs, forming a homogeneous dye bath.^85,86^ Upon immersion of the textile substrate, the ILs penetrate the amorphous regions of the fiber, promoting structural swelling and increasing molecular mobility within the polymer matrix.^87^ This enhanced free volume facilitates the diffusion of dye molecules into the fiber interior.^88^ Unlike conventional aqueous exhaustion dyeing, mass transfer in IL systems is primarily governed by solvent–polymer interactions rather than water-mediated diffusion.^88^

A notable advantage of IL-assisted dyeing is the potential for efficient solvent recovery and reuse due to their negligible vapor pressure and low volatility. However, several challenges remain for large-scale industrial implementation, including the high viscosity of many ILs, their relatively high cost, and toxicity concerns associated with certain IL formulations.^84,89^

DESs represent a new generation of low-cost solvent systems formed through the complexation of a hydrogen bond acceptor and a hydrogen bond donor.^90^ The resulting eutectic mixture exhibits a melting point significantly lower than that of its individual components, enabling its use as a liquid dyeing medium.^91^ In DES-based dyeing, the eutectic solvent acts as both dye carrier and fiber-swelling agent (Fig. 6).^92^ The extensive hydrogen-bonding network within DES systems enhances dye solubility and facilitates penetration into fiber structures, particularly in cellulosic substrates.^93^ Fiber swelling induced by hydrogen-bond interactions between DES components and hydroxyl groups in cellulose increases dye accessibility and promotes efficient diffusion.^94^ The dyeing mechanism in DES media primarily involves solvent-mediated diffusion followed by physical adsorption or chemical fixation, depending on the dye chemistry. Compared to ILs, DES systems are generally more economical, easier to synthesize, and often exhibit lower toxicity profiles.^95^ Nevertheless, their relatively high viscosity and limited industrial-scale validation remain significant constraints. DES-based dyeing is therefore considered an emerging sustainable alternative within solvent-assisted dyeing technologies, particularly for natural fibers.

**Fig. 6:**
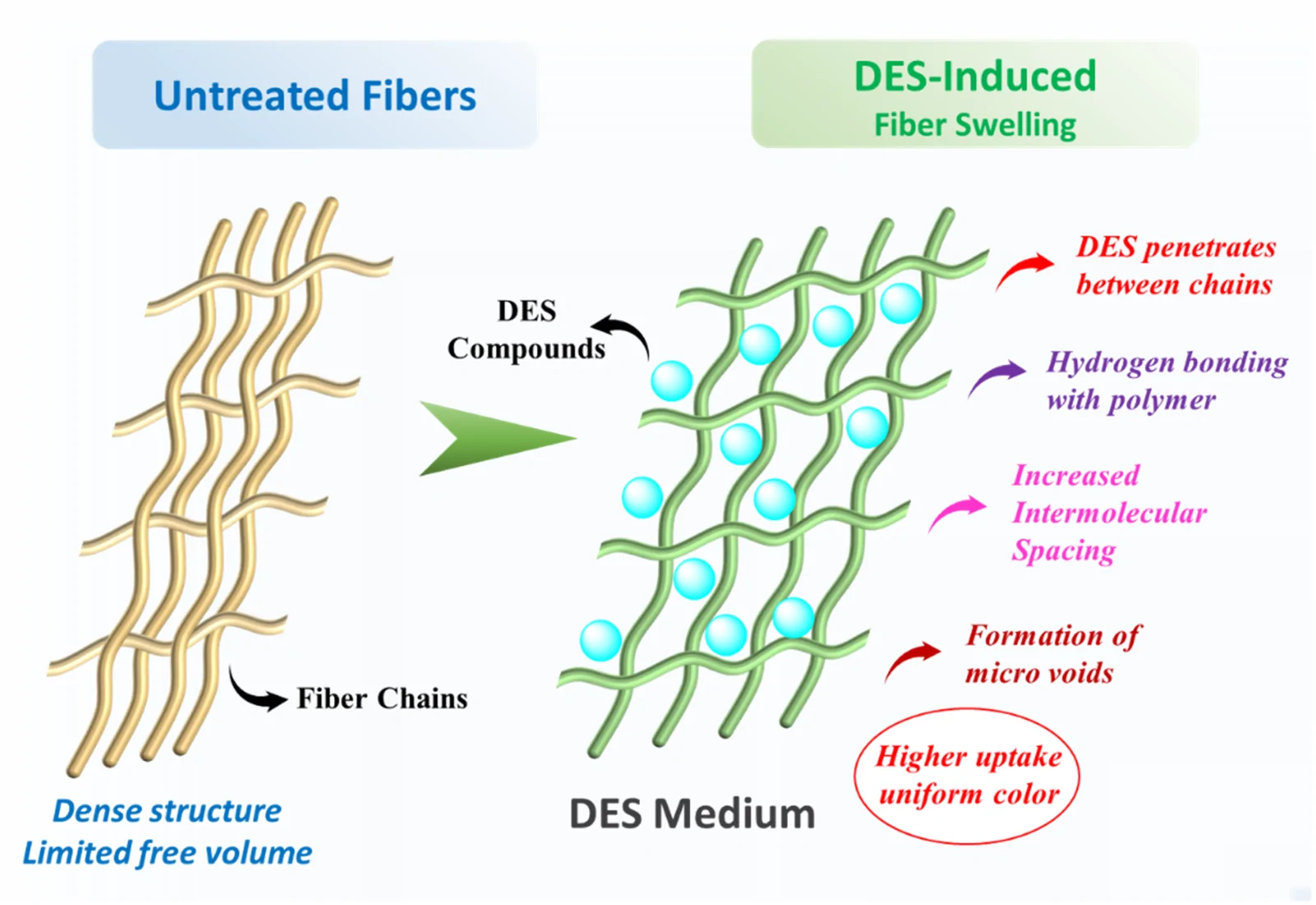
Shows swelling of fibers by DES facilitates dye penetration and fixation.

Table 9 summarizes IL/DES dyeing studies on various textile substrates; none of the listed studies used DR dyes. We propose that ILs and DESs could serve as promising solvent systems for DR dyes by simultaneously facilitating the dissolution and transport of the disperse component, while providing a medium for fiber interaction and covalent fixation of the reactive moieties. Such an approach could potentially enable solvent-assisted dyeing of hybrid dyes, maintaining color performance and fixation while minimizing environmental impact. Future studies are encouraged to explore this unexplored area, focusing on solvent selection, dye–fiber interaction mechanisms, process optimization, and solvent recovery strategies. Investigating DR dyes in IL and DES media could not only expand the scope of sustainable textile processing but also bridge a critical gap in the current literature.

**Table 9:** Summary of IL/DES dyeing of various textile fibers

Fiber	Dye		IL/DES	owf%	L : R	Temp./time	K/S	Color fastness (wash/rub/light)	Ref.
Wool	Madder Red	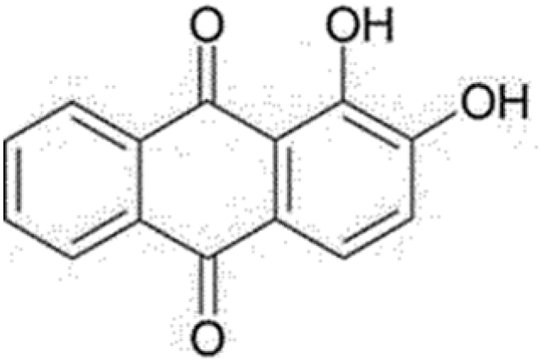	IL	1–2	1 : 40	80 °C – 45 min	N.P.	3–4/N.P./6–7	96
Wool	*Reseda lutea*	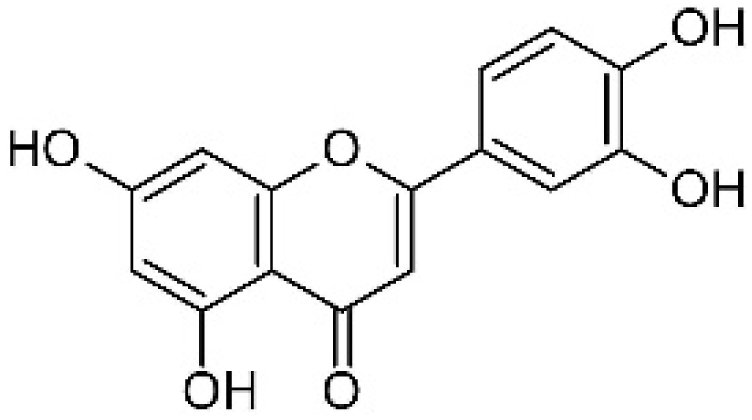	IL	1–2	1 : 40	80 °C – 45 min	N.P.	5/N.P./7–8	96
Wool	C.I. Acid Red 249	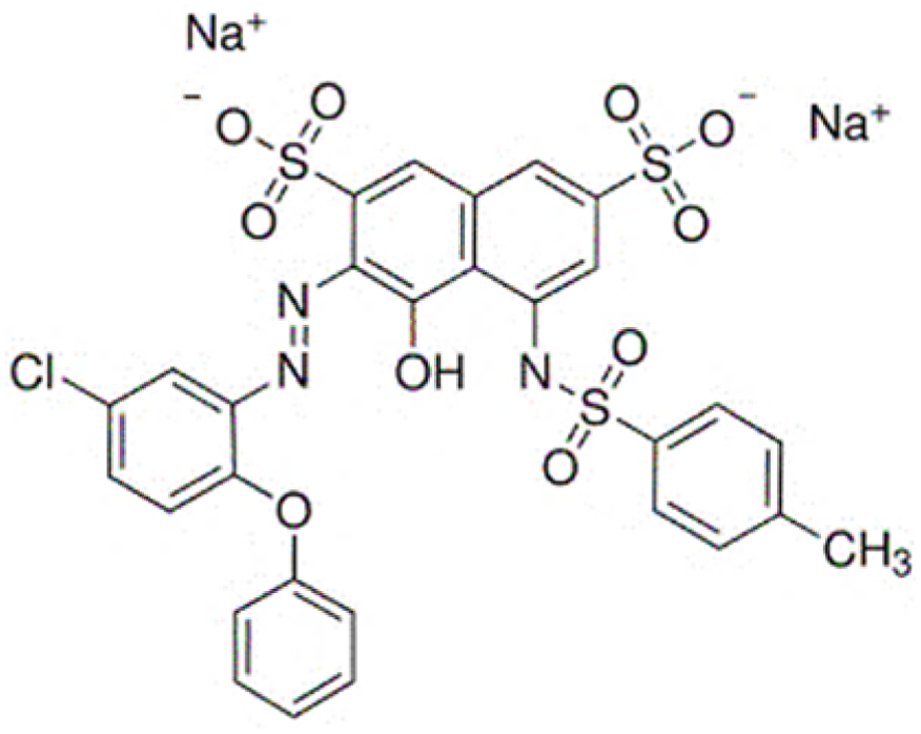	IL	2	1 : 100	100 °C – 120 min	23	4/5/N.P.	97
Cotton	C.I. Reactive Red 238	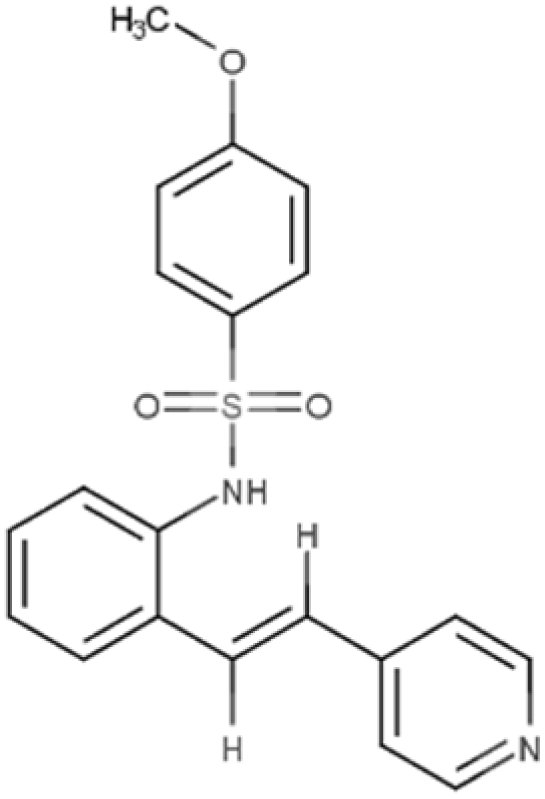	IL	1	N.P.	60 °C – 120 min	4.9	5/4–5/N.P.	98
Wool	C.I. Acid Blue 7	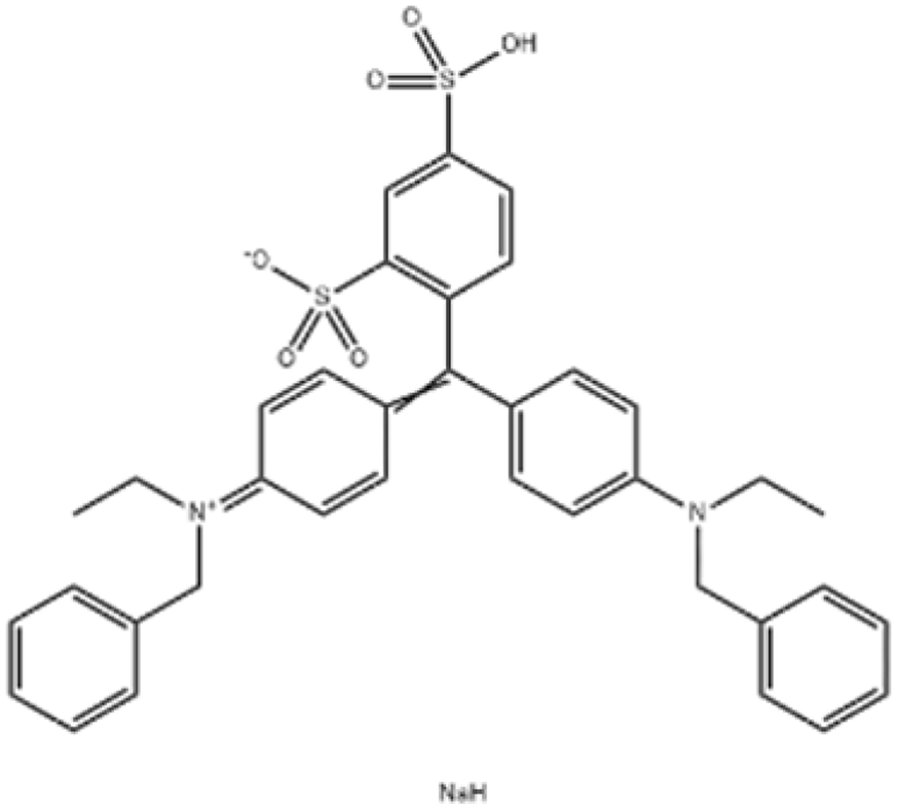	IL	4	1 : 100	100 °C – 120 min	31.1	4/3–4/N.P.	99
Polyester	C.I. Disperse Red 343	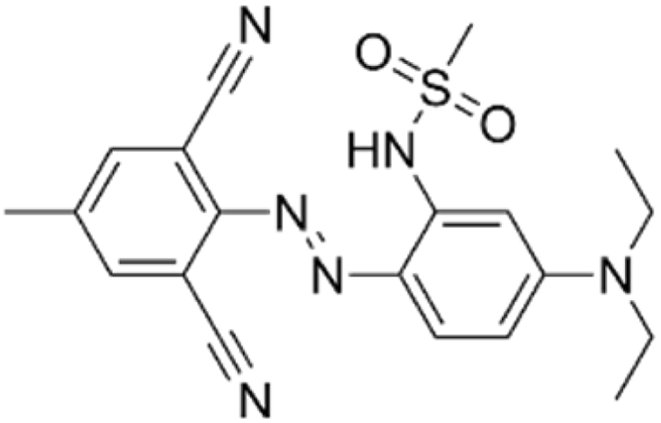	DES	4	1 : 50	130 °C – 30 min	N.P.	4–5/N.P./N.P.	100
Polyester	C.I. Disperse Violet 26	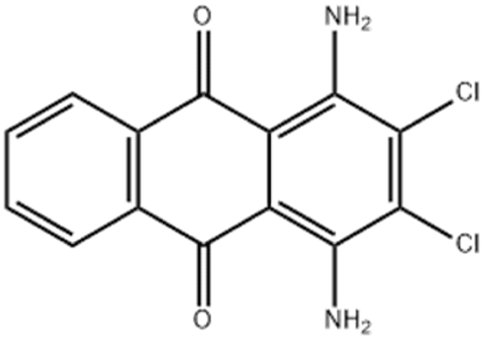	DES	1–5	1 : 50	100 °C – 45 min	22	4–5/N.P./7	101
Polylactic acid	C.I. Disperse Red 167	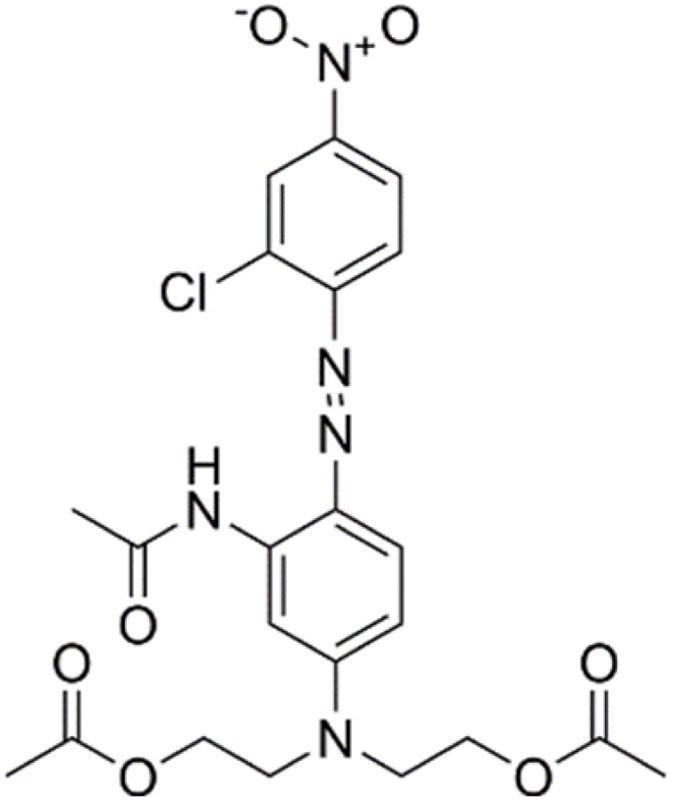	DES	2	1 : 100	90 °C – 30 min	10.8	N.P.	102
Polylactic acid	C.I. Disperse Orange 30	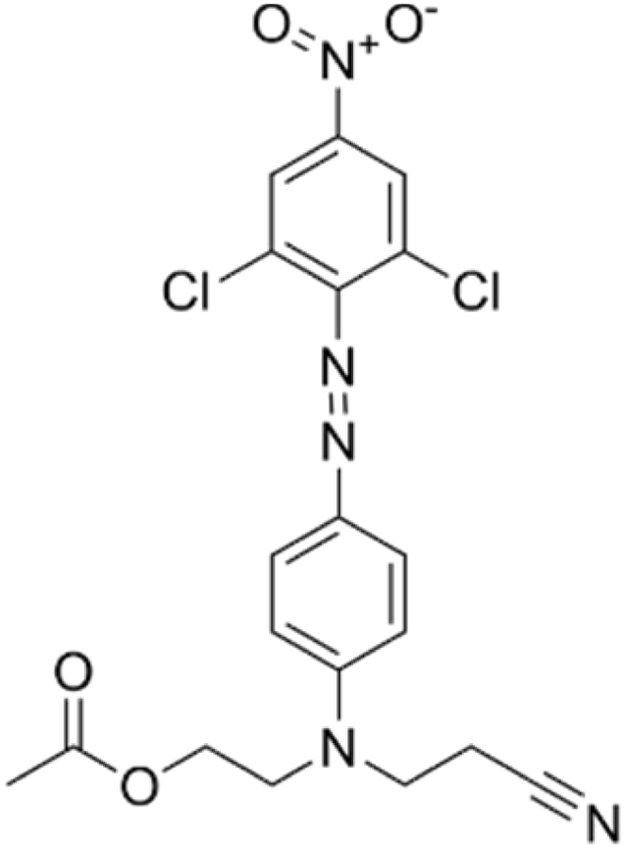	DES	2	1 : 100	90 °C – 30 min	13	N.P.	102
Cellulose nanofibers	C.I. Reactive Red 195	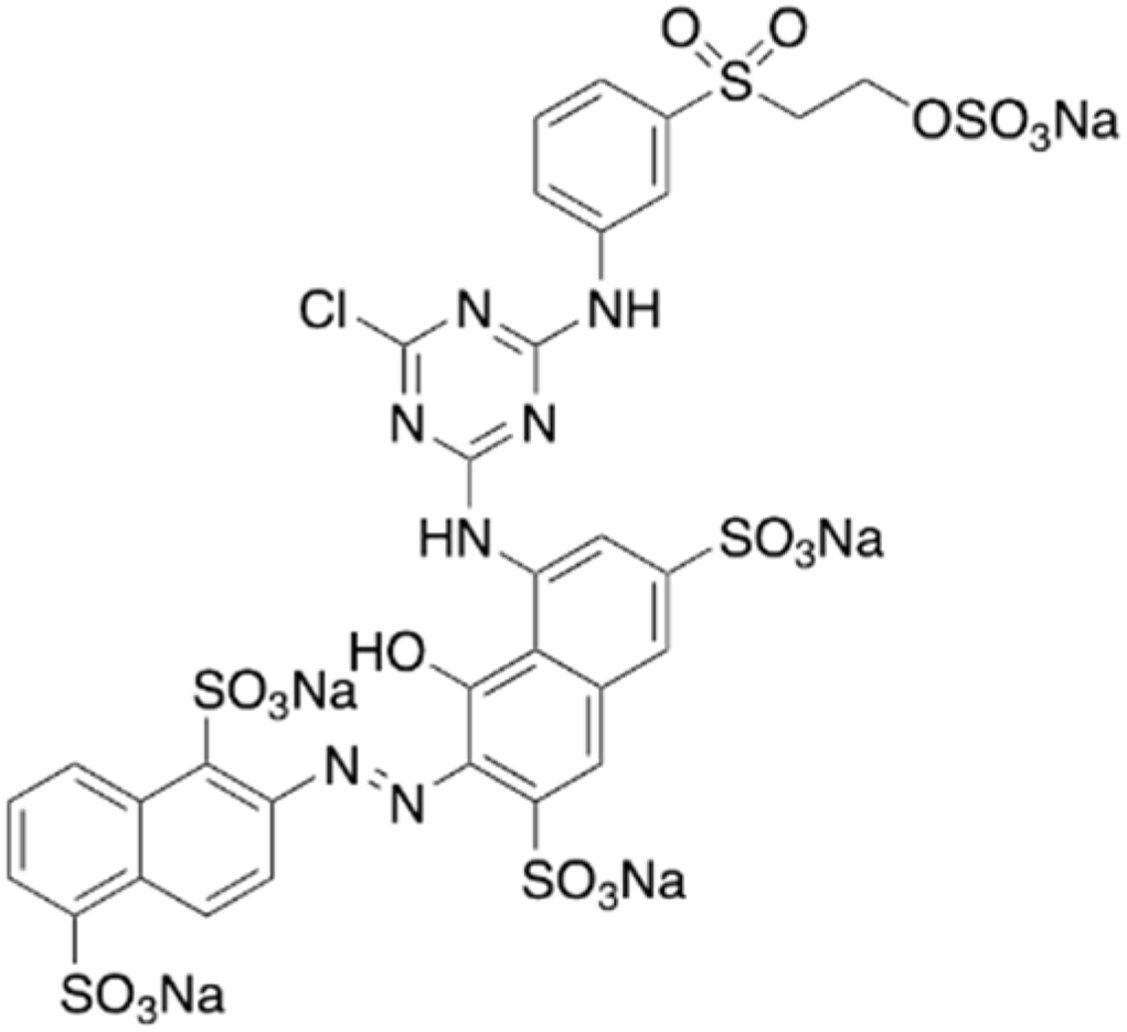	DES	2–5	1 : 100	30–80 °C – 10–70 min	3.2	N.P.	103

### 4.4. Dyeing in supercritical carbon dioxide (SC-CO_2_)

SC-CO_2_ dyeing is an innovative, water-free method for coloring textiles, primarily synthetic fibers like polyester.^104^ This process uses carbon dioxide in a supercritical state, a phase where it exhibits properties of both gases and liquids, as the dyeing medium instead of water.^105^ This technology has gained significant attention as a sustainable alternative to conventional dyeing processes.^105^

SC-CO_2_ is created by heating carbon dioxide above 31 °C and compressing it beyond 74 bar.^105^ In this state, CO_2_ acts as a powerful nonpolar solvent, making it ideal for dyeing hydrophobic (water-repelling) fibers like polyester with disperse dyes, which are also nonpolar.^106^ The SC-CO_2_ fluid facilitates dye uptake by swelling the polymer structure of polyester, thereby increasing the free volume and molecular mobility of the polymer chains. This enables molecular diffusion of dye into the amorphous regions of the fibers.^107^ In addition, the low viscosity of SC-CO_2_ promotes uniform circulation through the yarn and fabric structure, ensuring even dye distribution and reduced energy consumption.^108^

CO_2_ is particularly suitable for this technology due to its unique characteristics: non-flammability, non-toxicity, widespread availability, and relatively low critical point (31 °C and 74 bar).^109^ Above the critical point, CO_2_ exhibits properties between liquids and gases: high density similar to liquids, which enables dissolution of hydrophobic dyes, and low viscosity with a high diffusion coefficient similar to gases, leading to improved mass transfer and reduced dyeing time.^110^ Increased density in the supercritical region reduces intermolecular distances and enhances solvent–solute interactions, while the fluid maintains its gas-like properties such as easy pumpability and high permeability.^111^

The dyeing mechanism in this process consists of three main stages: dissolution of the dye in SC-CO_2_, transfer to the fiber surface, and finally absorption and diffusion into the polymer structure.^112^ SC-CO_2_ swells the structure of many polymers by reducing their glass transition temperature (*T*_g_), thereby increasing permeability; consequently, mass transfer and dye diffusion within the polymer matrix occur more rapidly.^113,114^

From an analytical perspective, the dyeing system comprising dye, SC-CO_2_, and fibers is considered a three-component/three-phase system, consisting of the phase (carbon dioxide), the coloring material, and the fiber-forming polymer.^115^ For modeling simplification, it is typically assumed that the dye is a pure component, and the solubility of CO_2_ and polymer in the dye, as well as polymer solubility in SC-CO_2_, are negligible. However, the presence of other components can significantly influence the dyeing process.^116,117^

In the practical dyeing process, the sample is wrapped around a perforated stainless steel tube and placed inside an autoclave. After the system is sealed, the temperature is increased simultaneously with CO_2_ pumping. At approximately 80–130 °C under constant volume conditions, pressure reaches around 20–30 MPa.^54^ Dyeing time typically extends to 60–180 minutes. Upon completion of dyeing, the pressure is partially reduced to separate and recover the dissolved dye. Subsequently, while the system remains pressurized, pure preheated supercritical CO_2_ is circulated through the vessel to wash off unfixed dye from the fabric surface (clearing step). Finally, the system is depressurized to atmospheric pressure, and the dyed fabric is removed.^105^

The most significant advantage of SC-CO_2_ Dyeing is the complete elimination of dyeing wastewater, thereby removing the need to treat and discharge polluted effluents that typically contain dyes, salts, and auxiliary chemicals.^110^ By eliminating large aqueous dye baths, the process substantially reduces freshwater consumption and simplifies effluent management. Energy consumption can also be reduced by approximately 50%, primarily because conventional dyeing requires heating large volumes of water to elevated temperatures and subsequently drying the wet textiles after dyeing. In SC-CO_2_ dyeing, the process operates without water, eliminating the substantial thermal energy required for water heating and evaporation during drying. Since water has a high specific heat capacity, heating aqueous dye baths represents a major portion of energy use in traditional dyeing processes. In contrast, SC-CO_2_ dyeing requires only the compression and heating of CO_2_ to reach supercritical conditions, which generally demands significantly less energy than heating and evaporating large quantities of water. Furthermore, because textiles emerge from the dyeing process essentially dry, the energy-intensive drying step that normally follows aqueous dyeing is avoided. However, stentering may still be required for heat setting or dimensional control, and any aqueous pretreatment or afterwashing steps would still necessitate drying. Therefore, while SC-CO_2_ dyeing eliminates the drying step directly associated with the dye bath, it does not necessarily eliminate all drying operations in the overall textile finishing sequence. The high diffusivity and low viscosity of supercritical CO_2_ also enhance dye transport within the fiber matrix, enabling shorter dyeing cycles—typically around two hours for polyester–cotton blends.^118^ The process further reduces resource consumption by requiring little or no auxiliary chemicals, such as dispersing agents or surfactants, lowering chemical use by up to 90%. Importantly, more than 90% of the CO_2_ used in the system can be recovered and reused, improving both the environmental and economic sustainability of the process.^119^

Despite the success of SC-CO_2_ dyeing for synthetic fibers, dyeing natural fibers (*e.g.*, cotton, viscose) remains a fundamental challenge.^104^ CO_2_ is a nonpolar solvent and cannot adequately swell polar, hydrophilic fibers such as cotton to facilitate effective dye penetration. Moreover, conventional dyes used for natural fibers, including reactive, direct, and acid dyes, are generally insoluble in SC-CO_2_, preventing their efficient transport in the dyeing medium.^120^ While disperse dyes can dissolve in SC-CO_2_ and diffuse into hydrophobic fibers like polyester, they typically adsorb only physically onto natural fibers without forming chemical bonds, resulting in poor fixation and low color fastness. In this context, DR dyes may offer a potential strategy for natural-fiber dyeing in SC-CO_2_ because their hydrophobic segments can facilitate dissolution and transport within the nonpolar medium, while their reactive groups provide the chemical functionality required for covalent bonding with cellulose under conventional aqueous or alkaline dyeing conditions. However, the formation of such covalent bonds generally requires suitable activation and an alkaline environment to promote the nucleophilic reactivity of cellulose hydroxyl groups. These conditions are not inherently provided by nonpolar SC-CO_2_. Direct evidence for dye–cellulose covalent bonds remains limited and depends on the dye and treatment conditions; therefore, although DR dyes possess reactive groups capable of covalent fixation to cellulose, covalent fixation of DR dyes onto cotton under SC-CO_2_ dyeing conditions has not yet been conclusively demonstrated, and the interactions reported to date should be interpreted with caution rather than categorically classified as physical adsorption.

The results summarized in Table 10 demonstrate that DR dyes can be successfully applied to a wide range of fibers using SC-CO_2_ dyeing, including cotton, polyester, polyester–cotton blends, silk, wool, and nylon. For cotton, which is traditionally difficult to dye in SC-CO_2_ due to its hydrophilic nature, DR dyes produced moderate to high color strength values (K/S ≈ 0.3–30) depending on dye structure and process conditions. Many of these dyes also exhibited good wash and rubbing fastness (generally grades 4–5), indicating effective dye fixation. Similar performance trends were observed for protein fibers such as silk and wool, where moderate K/S values and good fastness were obtained, as well as for nylon, which exhibited particularly high color strengths (K/S up to ∼16) and excellent fastness properties. In blended substrates such as polyester/cotton, DR dyes enable simultaneous interaction with both fiber components, offering the potential for single-step coloration in waterless SC-CO_2_ systems. Overall, these results highlight the ability of DR dyes to overcome the traditional incompatibility between hydrophilic fibers and the nonpolar SC-CO_2_ medium, thereby expanding the applicability of waterless dyeing technologies to a broader range of textile materials.

**Table 10:** Overview of process parameters and dyeing performance of various textile fibers dyed with DR dyes in SC-CO_2_

Fiber	Dye	owf%	Pressure/temp./time	K/S	Color fastness (wash/rub/light)	Ref.
Cotton	DR8	0.5	20 MPa/80 °C/20 min	0.293	4–5/4–5/N.P.	48
	DR5	0.5	25 MPa/120 °C/1 h	3.86	4–5/N.P./4–5	33
	DR29	0.5	20 MPa/100 °C/1 h	15	4/5/N.P.	60
	DR30	0.5	20 MPa/100 °C/1 h	12	3–4/5/N.P.	60
	DR3a	0.5	200 bar/80–120 °C/1–3 h	9.9	3–4/4–5/N.P.	44
	DR3b	0.5	200 bar/80–120 °C/1–3 h	12.5	3/4–5/N.P.	44
	DR3c	0.5	200 bar/80–120 °C/1–3 h	14	2–3/4–5/N.P.	44
	DR7	—	30 MPa/110 °C/1 h	0.67	3/4/N.P.	47
	DR31	0.5	20 MPa/100 °C/1 h	14	4/5/N.P.	61
	DR13	2–3	28 MPa/130 °C/2 h	11.8	5/5/4–5	53
	DR^1^	—	25 MPa/120 °C/2 h	30.21	3/3/N.P.	121
	DR17a	0.5	5–20 MPa/60–120 °C/1–3 h	2.2	3/4–5/N.P.	54
	DR17b	0.5	5–20 MPa/60–120 °C/1–3 h	0.6	3/4/N.P.	54
	DR20	0.5	25 MPa/120 °C/1 h	10.44	4–5/4–5/1	26
	DR21	1–4	25 MPa/100–120 °C/90 min	9.38	5/5/4–5	19
	DR22	1–4	25 MPa/100–120 °C/90 min	6.65	5/5/4–5	19
Polyester/cotton	DR15	2.5	24 MPa/100 °C/1 h	8	4–5/4–5/N.P.	31
Polyester	DR^2^	0.2	278 bar/116 °C/2 h	12.2	N.P.	14
Silk	DR8	0.5	20 MPa/80 °C/20 min	1.108	4–5/4/N.P.	48
	DR^2^	0.2	278 bar/116 °C/2 h	4.93	N.P.	14
Wool	DR8	0.5	20 MPa/80 °C/20 min	1.373	4–5/4/N.P.	48
	DR^3^	2	23 MPa/100 °C/70 min	5.51	4/4–5/N.P.	122
	DR7	—	30 MPa/110 °C/1 h	0.78	3–4/3–4/N.P.	47
	DR^2^	0.2	278 bar/116 °C/2 h	4.71	N.P.	14
	DR14	0.5	20 MPa/80 °C/1 h	3.8	3/4–5/N.P.	18
	DR18a	3–4	25 MPa/110 °C/90 min	8.61	4–5/4/5	39
	DR18b	3–4	25 MPa/110 °C/90 min	6.99	4–5/4/5	39
Nylon	DR24	2	300 bar/130 °C/3 h	16.13	5/5/5	57
	DR18a	3–4	25 MPa/130 °C/90 min	16.63	5/5/5	39
	DR20	0.5	25 MPa/120 °C/1 h	12.17	4–5/4–5/2–3	26
	DR18b	3–4	25 MPa/130 °C/90 min	14.91	5/4–5/5	39
	DR5	0.5	25 MPa/120 °C/1 h	5.91	4/N.P./4	33
DR^1^	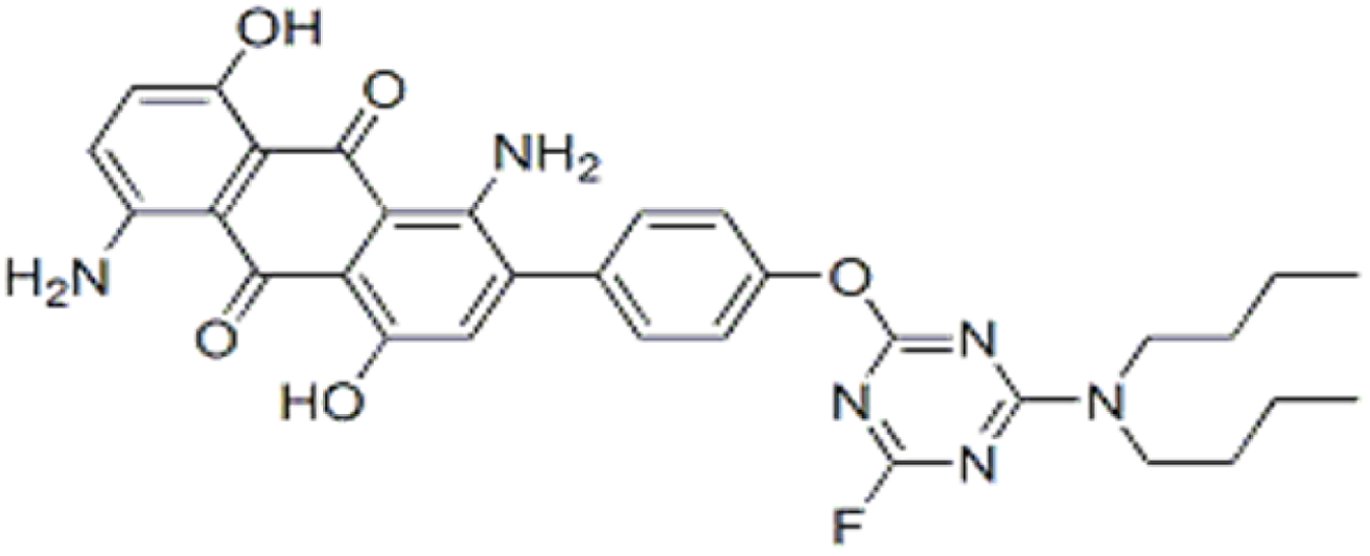					
DR^2^	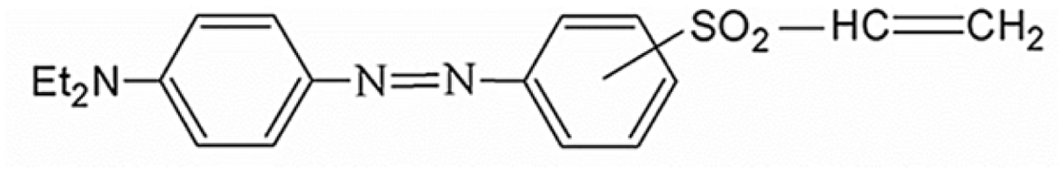					
DR^3^	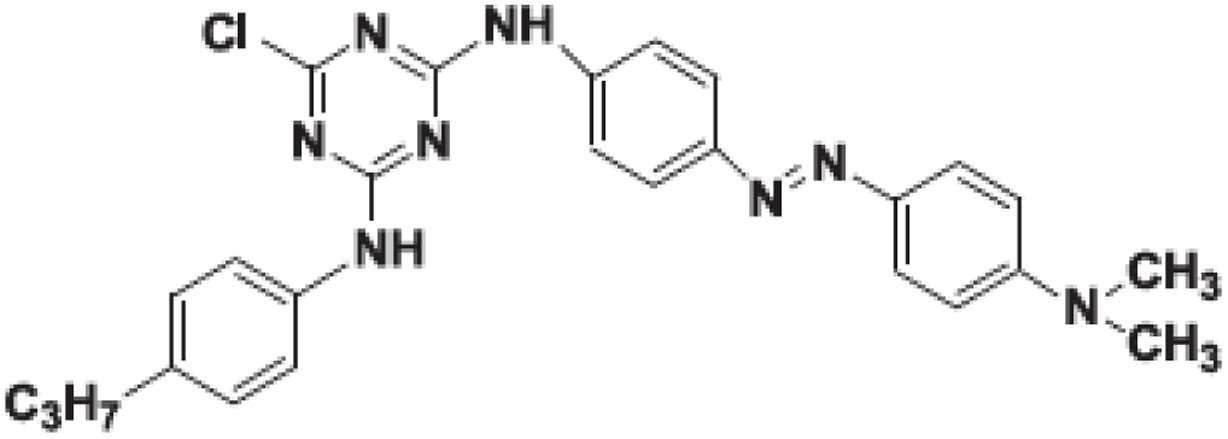					

Despite its environmental benefits and technical potential, the industrial adoption of SC-CO_2_ dyeing faces several challenges. High initial investment in specialized high-pressure equipment and precision control systems limits accessibility, particularly for smaller manufacturers.^67,123,124^ Dye development remains a constraint, as the technology was historically limited to disperse dyes for polyester, and although innovations such as DR dyes and pre-coating methods have emerged, the range of compatible dyes is still narrow, often requiring highly purified dyes and complicating dye bath recovery.^67^ Process-related issues, including minor CO_2_ losses per cycle and the lack of standardized guidelines for dye selection and process optimization, further hinder widespread implementation.^67^ Regarding the attachment mechanism of DR dyes to cotton in SC-CO_2_ medium, a critical limitation must be addressed: under SC-CO_2_ conditions (typically 80–120 °C, 20–30 MPa), the hydroxyl groups of cellulose are not activated for nucleophilic substitution because SC-CO_2_ is a non-polar to weakly polar medium that cannot deprotonate cellulose –OH groups (p*K*_a_ ∼ 13–14) in the absence of alkaline auxiliaries.^125^ To date, no peer-reviewed study has demonstrated direct covalent bond formation between conventional DR-type dyes and cotton in SC-CO_2_ medium. Consequently, for DR dyes in SC-CO_2_, the primary interactions remain physical adsorption (van der Waals forces and hydrogen bonding) rather than covalent attachment. This limitation explains the lower wash fastness reported for natural fibers dyed in SC-CO_2_ compared to conventional aqueous reactive dyeing, where alkaline conditions activate cellulose hydroxyl groups for nucleophilic attack on reactive dyes.

### 4.5. Inkjet digital printing

On the path to transforming the textile industry towards sustainability, it is sometimes necessary to completely rethink traditional paradigms. At the core of this transformation lies inkjet printing technology, which has fundamentally changed the nature of fabric coloration.^126^ Unlike traditional methods that require large amounts of water for preparation, transfer, and fixation, digital inkjet printing reduces water use in the production cycle considerably.^127^ In this technology, specially formulated inks, whether pigment, reactive, acid, or disperse, are sprayed directly onto the fabric through printheads. The fixation mechanism depends on the dye class: reactive dyes form covalent bonds with suitable fibers (*e.g.*, cellulose or protein), acid dyes primarily rely on ionic and noncovalent interactions with polyamide or protein fibers, disperse dyes diffuse into hydrophobic synthetic polymers, and pigments are bound to the fabric surface by a polymer binder film.^128^ After printing, the fabric is finished through a curing process with dry heat or steam, making inkjet printing one of the most sustainable coloration methods available.

Solvent-based inks do not automatically eliminate aqueous pretreatment or afterwashing, and binder-film fixation specifically concerns pigment/binder systems. Cite the actual ink/substrate/process supporting the claimed 99% water reduction.^129^

Beyond the obvious environmental benefits, inkjet fabric printing has brought remarkable achievements to the fields of design and production. This technology has broken the boundaries of creativity, making it possible to realize any design, regardless of its complexity.^130^ In traditional methods, each new design required expensive and time-consuming physical rollers or screens.^127^ Digital printing, by transferring directly from a digital file to fabric, has eliminated these limits entirely.^127^ Designers now enjoy a broad color gamut and fine design detail within the limits of the ink set, substrate, and printing system.

Modern inkjet systems use precisely formulated inks tailored to fiber chemistry, supporting water-efficient printing.^131^ Reactive dyes are widely used for cellulosic fibers (cotton, linen, viscose), forming stable covalent bonds with hydroxyl groups to ensure wash fastness.^132–135^ Disperse dyes, being nonionic and thermally mobile, are commonly used for hydrophobic fibers like polyester, transferring color *via* sublimation without aqueous media.^136–139^ Thus, dye selection in inkjet printing is dictated by fiber chemistry.

The main challenge arises when waterless printing on blended fabrics, such as polyester/cotton. Blended fibers require both covalent bonding and thermal diffusion, creating chemical and process conflicts. Reactive dyes need steaming and washing, while disperse dyes require heat fixation, often increasing energy use and water cycles.

DR dyes offer a potential solution by enabling the simultaneous dyeing of natural and synthetic fibers in a single step, without the need for extensive post-washing. However, progress in this area remains limited. The only reported study on the application of such dyes for digital textile printing explored a DR11 derivative containing a blocked isocyanate group. In this work, the researchers synthesized the dye and evaluated its performance using thermal transfer printing equipment on cotton, PET, and nylon fabrics. When applied to cotton at 210 °C, the synthesized DR11 achieved a K/S of 2.1, surpassing that of commercial dyes and demonstrating improved wash and light fastness. While this study confirms the potential of introducing isocyanate groups into reactive disperse dyes, the results are based on thermal transfer experiments and do not constitute a demonstration of a fully formulated inkjet-ready DR formulation. These findings highlight the potential of hybrid DR dye systems for more sustainable, waterless digital printing processes, but further research is required to validate their performance in an actual inkjet printing context.

Future developments in inkjet printing will focus on creating hybrid ink systems capable of interacting with multiple fiber chemistries while preserving the environmental benefits of waterless processing. Advancing such technologies will be essential for printing blended textiles and expanding sustainable digital manufacturing. Despite current challenges, including high ink costs and relatively slower production speeds, continued innovation is expected to strengthen the role of digital printing as a key technology for the future of sustainable textile production.^127^

## 5. Comparative assessment, challenges, limitations, and future directions

### 5.1. DR dyes

The development of DR dyes represents a significant advancement in textile coloration chemistry, particularly in the context of sustainable and waterless dyeing technologies. The global textile market is dominated by polyester and cellulosic fibers, especially cotton and regenerated cellulose, which together account for the majority of textile production. However, these fibers require fundamentally different dye classes: polyester is typically dyed with disperse dyes, while cotton and other cellulosic fibers rely on reactive dyes. This difference in dye chemistry creates a major processing challenge for blended textiles such as polyester–cotton, which usually require sequential dyeing processes involving separate dye baths, increased water consumption, higher energy use, and greater chemical inputs. As a result, there is a strong motivation to develop dye systems capable of simultaneously interacting with both hydrophobic and hydrophilic fibers while reducing environmental impact.

DR dyes address this challenge by combining the hydrophobic characteristics of disperse dyes with the covalent bonding capability of reactive dyes within a single molecular structure. This dual functionality enables DR dyes to diffuse into hydrophobic synthetic fibers while also forming chemical bonds with nucleophilic sites on cellulosic substrates. DR dyes are suitable for solvent-assisted dyeing systems, such as supercritical CO_2_ and solvent-based processes, supporting the development of waterless textile coloration technologies.

Despite these advantages, several technical and practical challenges remain. One of the primary difficulties lies in achieving an optimal balance between hydrophobicity and reactivity within the dye molecule. Excessive hydrophobicity may enhance solubility in nonpolar media but reduce reactivity toward cellulosic fibers, whereas highly reactive functional groups may require aqueous conditions for activation or may undergo hydrolysis during processing. Additionally, the diffusion of DR dyes into highly crystalline fibers such as cotton can be limited, particularly under waterless conditions where fiber swelling is reduced. Process parameters such as temperature, pressure, and solvent selection therefore play a critical role in determining dye uptake and fixation efficiency.

Another limitation relates to dye stability and fixation efficiency. Certain reactive groups, including dichlorotriazine and vinyl sulfone moieties, may exhibit limited reactivity in non-aqueous environments or may require carefully controlled conditions to achieve effective covalent bonding. In some cases, incomplete fixation may still lead to dye loss during washing, reducing overall process efficiency.

Another important challenge relates to the commercialization and regulatory approval of DR dyes. Unlike conventional dye classes that are already widely produced and registered by chemical manufacturers, DR dyes are relatively new compounds and are not yet established in large-scale industrial production. Bringing new dye molecules to the market requires extensive registration, safety evaluation, and regulatory certification to comply with stringent chemical regulations and environmental standards. These processes involve significant costs associated with toxicological testing, environmental impact assessments, and compliance with international regulatory frameworks, such as REACH and other chemical safety regulations. Consequently, the development and commercialization of DR dyes require substantial financial investment and long development timelines, which may discourage manufacturers from adopting these new colorants unless clear technical and economic advantages over existing dyes can be demonstrated.

Industrial implementation also faces process-related and economic challenges. Waterless dyeing technologies, such as supercritical CO_2_ systems, require specialized high-pressure equipment and careful solvent management, which can increase capital costs. Similarly, solvent-based dyeing systems require efficient solvent recovery and recycling infrastructure to ensure economic viability and environmental sustainability. Without such systems, the environmental advantages of waterless dyeing may be diminished.

Looking forward, several research directions are likely to play a key role in advancing DR dye technology. First, further efforts in molecular design are needed to optimize the balance between hydrophobic transport properties and reactive functionality, enabling efficient dye diffusion and fixation in both aqueous and non-aqueous media. Second, the development of DR dyes tailored specifically for waterless dyeing platforms, such as supercritical CO_2_ or silicone-based solvents, could significantly expand their applicability to cotton and blended textiles. Third, improved understanding of dye–fiber interactions and reaction mechanisms under non-aqueous conditions will help guide the design of more effective dye structures. Finally, integration of DR dye technology with green solvents, closed-loop processing systems, and life-cycle assessment frameworks will be essential for demonstrating the true sustainability benefits of these colorants.

### 5.2. Waterless dyeing technologies

The pursuit of sustainable textile processing has led to the development of several alternative dyeing technologies aimed at eliminating or drastically reducing water consumption. This section provides a comparative evaluation of the key waterless dyeing methods discussed in this paper, focusing on their principles, applicability, advantages, and limitations. The comparison is structured around critical parameters, including water consumption, energy efficiency, dye fixation rate, suitability for different fiber types, and commercial maturity. Table 11 presents a comparative summary of these waterless dyeing technologies across key performance indicators. Table 12 presents the market size, industrial adoption level, and leading companies for five waterless textile dyeing technologies, highlighting the degree of commercialization and current industrial players.

**Table 11:** Comparison of waterless and low-water coloration technologies: technical, environmental, and economic aspects^67,96,140–146^

Technology	Water reduction	Energy consumption	Fiber suitability	Environmental impact	Economic impact
SC-CO_2_ dyeing	>95%	Moderate-high	Synthetic (primary), natural (emerging)	Zero wastewater, little or no auxiliaries, CO_2_ reused, 99% solvent recycled, no effluent	Very high capital investment ($1–3M per vessel), expensive specialized dyes, limited commercial disperse dyes available
Inkjet digital	30–50%	Low-moderate	All fibers	Precise chemical application, reduced wastewater, lower carbon footprint, minimal sample waste	Lower inventory costs, fast sampling reduces time-to-market, premium pricing for customization, and reduced labor costs
ILs/DES	70–90%	No significant reduction	Natural fibers	Eliminates water but introduces novel solvents; 5–10% solvent loss mixed with washing effluent; some ILs toxic and non-biodegradable; DES more biodegradable	High solvent costs, recovery energy-intensive, economies of scale not yet achieved
Reverse micelle	80–90%	Marginal reduction	All fibers	Reduces water but uses organic solvents and surfactants; ∼5% solvent loss; VOC emissions possible; solvents often harmful/flammable	Surfactant costs add 15–25% to the chemical budget, and complex recovery systems increase capital and operating costs
Solvent-assisted	85–95%	Moderate	All fibers	If more than 95% of the solvent is recovered, wastewater production is minimized; but most solvents are harmful and flammable; VOC risk; ∼5–10% solvent loss	Moderate capital for enclosed equipment; economic viability tied to solvent recovery efficiency (>99% required)
Conventional (baseline)	0% (150–200 L kg^−1^)	High	All fibers	Massive wastewater, high thermal energy for drying, and effluent treatment costs are externalized	Low capital entry but increasing water/wastewater regulation costs; vulnerable to water scarcity pricing

**Table 12:** Waterless dyeing methods: market & industry status^12,67,147,148^

Technique	Market size/forecast	Industrial/commercial adoption	Leading companies/developers
SC-CO_2_ dyeing	Identified as the most commercially advanced waterless dyeing technology in market forecasts, frequently highlighted as the leading segment of the waterless dyeing technology market	Industrial adoption underway; systems have been built and used for polyester dyeing in real production settings, with closed-loop CO_2_ recovery and recycling	DyeCoo® Textile Systems (Netherlands); industrial systems installed for polyester dyeing
			Deven Supercriticals (India); industrial SC-CO_2_ implementations
			Qarboon (France); SC-CO_2_ dyeing equipment developer
Inkjet digital printing	USD 5.8 billion (2024) global digital textile printing market; projected ≈ USD 11.6 billion by 2030 at ∼12.7% CAGR	Fully commercial & widely adopted in textiles	Kornit Digital Ltd; major industrial inkjet textile printers
			Epson, Mimaki, Roland DG; industrial textile printers are used globally
ILs/DES dyeing	There is no specific market value report	Not industrialized; mainly at the research, pilot study, or small-scale trial level	N.P.
Reverse micelle dyeing	There is no specific market value report	Not industrialized; mainly at the research, pilot study, or small-scale trial level	N.P.
Solvent-assisted dyeing	There is no specific market value report	Not industrialized; mainly at the research, pilot study, or small-scale trial level	N.P.

Based on the comparative evaluation of waterless dyeing technologies and their interactions with DR dyes, several conclusions can be drawn regarding their industrial applicability and potential for sustainable textile coloration.

Inkjet printing is commercially established, but the applicability of DR dyes remains at an early experimental stage. Industrial implementation requires validation of formulations, fixation, fastness, and post-treatment needs. Furthermore, inkjet printing requires minimal post-treatment washing, offers economic feasibility through reduced inventory and accelerated sampling, and is immediately implementable in industrial settings.

In contrast, SC-CO_2_ dyeing demonstrates significant long-term potential, particularly for polyester applications with conventional disperse dyes. Although DR dyes can facilitate the dyeing of natural fibers in SC-CO_2_ systems, challenges remain with respect to wash fastness and the limited commercial availability of suitable dyes. High capital investment requirements further restrict the widespread adoption of this method, confining its current use to specialized applications where the elimination of water is critical.

Reverse micelle and solvent-assisted dyeing techniques offer promising solutions at the laboratory scale, particularly for blended fabrics, due to their compatibility with both hydrophobic and hydrophilic fibers. D5-based solvent systems show potential for DR applications; however, their economic viability requires highly efficient recovery systems, and they have not yet reached industrial feasibility for commercial production.

ILs and DES-assisted dyeing remain predominantly research-oriented. Although potentially suitable for transporting DR dyes into natural fibers, practical implementation is hindered by challenges in solvent removal, recovery, and high costs, as well as potential toxicity concerns, particularly with ILs, which limit industrial adoption.

Although waterless dyeing technologies offer substantial environmental advantages, no comprehensive LCA has specifically evaluated DR dyes. Therefore, the sustainability potential of DR dyes currently relies on reported process parameters and LCA data from related platforms rather than dye-specific cradle-to-gate assessments.

For SC-CO_2_ dyeing, Johannesson identified electricity consumption and CO_2_ losses as major environmental hotspots, emphasizing the importance of energy optimization, CO_2_ recovery, and low-carbon electricity.^149^ Industrial assessments of DyeCoo technology reported greenhouse gas emission reductions of 58% compared with conventional aqueous dyeing, while optimized scenarios involving renewable electricity and 99% CO_2_ recovery reduced the carbon footprint from 3.91 to 1.60 kg CO_2_-eq. per kg dyed polyester.^119,150,151^

For ionic liquid dyeing, [Bmim]Cl-based PET dyeing achieved reductions of up to 71.88% in global warming potential and 88.93% in water consumption, with further improvements following solvent recovery and reuse.^86^ D5 solvent dyeing reduced water consumption by 61.30–79.95% and greenhouse gas emissions by 43.70%, although increased ecotoxicity and abiotic depletion potential were reported when solvent losses were insufficiently controlled.^152^ For inkjet printing, reported water savings of 30–50% remain primarily process-level estimates rather than comprehensive LCA results.^127^

Despite these findings, no LCA study has specifically evaluated DR dyes in waterless dyeing platforms. This represents a significant research gap, particularly because the environmental performance of DR dyes depends not only on the reduction of water and auxiliary chemicals during dyeing, but also on the impacts associated with dye synthesis, solvent compatibility, energy consumption, recovery efficiency, and end-of-life behavior. Future studies should therefore: (i) quantify the environmental footprint of DR dye synthesis, including reagent toxicity, solvent consumption, and process energy demand; (ii) assess the energy and water savings associated with DR dye application in SC-CO_2_, D5, and IL/DES-based systems; (iii) investigate end-of-life aspects, including dye leaching, persistence, and recyclability; and (iv) compare DR dyeing systems with conventional reactive and disperse dyeing on a consistent functional-unit basis. Accordingly, the sustainability potential of DR dyes can currently be discussed only on a qualitative and indicative basis, rather than through definitive, dye-specific comparative LCA results.

## 6. Conclusion

The development of DR dyes represents a significant step forward in addressing the long-standing challenge of coloring blended textiles, particularly polyester–cotton fabrics, which currently require multi-stage aqueous processes with high environmental costs. By integrating hydrophobic segments for synthetic fiber penetration with electrophilic groups designed for covalent attachment to cellulosic substrates under alkaline or aqueous conditions, DR dyes offer a conceptually elegant solution that could eliminate the need for sequential dyeing protocols. Nevertheless, as this review has systematically documented, the experimental reality lags considerably behind the theoretical promise. Among the five waterless platforms examined, only SC-CO_2_ has received substantial research attention, with numerous studies confirming the applicability of various DR dye architectures to cotton, polyester, and protein-based fibers, although covalent fixation on cotton in SC-CO_2_ has not yet been demonstrated, and interactions remain predominantly physical adsorption. Solvent-assisted dyeing in D5 has been explored in a single investigation, while inkjet printing, reverse micelle systems, and ILs/DES media remain either minimally tested or completely uncharted territory for DR dyes. This pronounced asymmetry carries important implications. For researchers, the priority should shift toward systematic performance validation in platforms where data are absent, particularly ILs/DES and reverse micelle systems. For industry, adoption of DR dyes in SC-CO_2_ and inkjet technologies will require dedicated formulation work and fixation optimization to achieve performance parity with established dye classes. From a broader perspective, no universal waterless solution currently exists. SC-CO_2_ excels for polyester but struggles with cellulose fixation; inkjet offers design flexibility but at lower throughput; solvent systems face recovery and cost barriers; and ILs/DES remain technologically immature. Looking ahead, the future of DR dyes hinges on several priorities: (i) design of reactive groups with enhanced fixation efficiency in non-aqueous media; (ii) development of solvent-compatible formulations that maintain stability and color strength; (iii) integration of closed-loop recovery systems for expensive solvents and CO_2_; and (iv) rigorous life cycle assessments to quantify environmental footprints. Crucially, without dedicated experimental effort in currently unexplored platforms, the full potential of DR dyes will remain unrealized. Their promise is genuine, but translation into practical impact demands sustained investment and a willingness to confront the limitations exposed by this review. In this sense, DR dyes embody not a finished achievement, but rather a compelling direction for sustainable textile chemistry, one that must be earned through empirical rigor, not merely asserted through conceptual appeal.

## Author contributions

Mousa Sadeghi-Kiakhani: conceptualization, funding acquisition, investigation, writing – original draft, writing – review & editing. Mohammad-Mahdi Norouzi: writing – original draft, data collection, writing – review & editing, visualization. Elaheh Hashemi: writing – review & editing. Ali R. Tehrani-Bagha: conceptualization, writing – review & editing.

## Conflicts of interest

The authors declare that they have no known competing financial interests or personal relationships that could have appeared to influence the work reported in this paper.

## Data Availability

No new experimental data were generated in this review. The information supporting its conclusions is drawn from the publications cited in the article.
